# Depletion of lamins B1 and B2 promotes chromatin mobility and induces differential gene expression by a mesoscale-motion-dependent mechanism

**DOI:** 10.1186/s13059-024-03212-y

**Published:** 2024-03-22

**Authors:** Emily M. Pujadas Liwag, Xiaolong Wei, Nicolas Acosta, Lucas M. Carter, Jiekun Yang, Luay M. Almassalha, Surbhi Jain, Ali Daneshkhah, Suhas S. P. Rao, Fidan Seker-Polat, Kyle L. MacQuarrie, Joe Ibarra, Vasundhara Agrawal, Erez Lieberman Aiden, Masato T. Kanemaki, Vadim Backman, Mazhar Adli

**Affiliations:** 1 Department of Biomedical Engineering, Northwestern University, Evanston, IL 60208 USA; 2 IBIS Interdisciplinary Biological Sciences Graduate Program, Northwestern University, Evanston, USA; 3 Center for Physical Genomics and Engineering, Northwestern University, Evanston, IL 60208 USA; 4 Department of Surgery, University of Virginia School of Medicine, Charlottesville, VA 22903 USA; 5 Computer Science and Artificial Intelligence Laboratory, Massachusetts Institute of Technology, Cambridge, MA 02139 USA; 6 Broad Institute of MIT and Harvard, Cambridge, MA 02142 USA; 7 Department of Gastroenterology and Hepatology, Northwestern Memorial Hospital, Chicago, IL 60611 USA; 8 The Center for Genome Architecture, Baylor College of Medicine, Houston, TX 77030 USA; 9 School of Medicine, Stanford University, Stanford, CA 94305 USA; 10 Department of Molecular and Human Genetics, Baylor College of Medicine, Houston, TX 77030 USA; 11 Feinberg School of Medicine, Robert Lurie Comprehensive Cancer Center, Department of Obstetrics and Gynecology, Northwestern University, Chicago, IL 60611 USA; 12 Feinberg School of Medicine, Robert Lurie Comprehensive Cancer Center, Department of Pediatrics, Northwestern University, Chicago, IL 60611 USA; 13 Stanley Manne Children’s Research Institute, Ann & Robert H. Lurie Children’s Hospital of Chicago, Chicago, IL USA; 14 Department of Chromosome Science, National Institute of Genetics, Mishima, Shizuoka 411-8540 Japan; 15 Graduate Institute for Advanced Studies, SOKENDAI, Mishima, Shizuoka, 411-8540 Japan; 16 Department of Biological Science, The University of Tokyo, Tokyo, 113-0033 Japan; 17 Center for Theoretical Biological Physics, Rice University, Houston, TX 77030 USA; 18 Departments of Computer Science and Computational and Applied Mathematics, Rice University, Houston, TX 77030 USA

**Keywords:** Nuclear lamina, Auxin-inducible degron system, 3D chromatin organization, Lamin-associated domains, Topologically associated domains, Partial Wave Spectroscopic Microscopy, CRISPR-Sirius; in situ Hi-C

## Abstract

**Background:**

B-type lamins are critical nuclear envelope proteins that interact with the three-dimensional genomic architecture. However, identifying the direct roles of B-lamins on dynamic genome organization has been challenging as their joint depletion severely impacts cell viability. To overcome this, we engineered mammalian cells to rapidly and completely degrade endogenous B-type lamins using Auxin-inducible degron technology.

**Results:**

Using live-cell Dual Partial Wave Spectroscopic (Dual-PWS) microscopy, Stochastic Optical Reconstruction Microscopy (STORM), in situ Hi-C, CRISPR-Sirius, and fluorescence in situ hybridization (FISH), we demonstrate that lamin B1 and lamin B2 are critical structural components of the nuclear periphery that create a repressive compartment for peripheral-associated genes. Lamin B1 and lamin B2 depletion minimally alters higher-order chromatin folding but disrupts cell morphology, significantly increases chromatin mobility, redistributes both constitutive and facultative heterochromatin, and induces differential gene expression both within and near lamin-associated domain (LAD) boundaries. Critically, we demonstrate that chromatin territories expand as upregulated genes within LADs radially shift inwards. Our results indicate that the mechanism of action of B-type lamins comes from their role in constraining chromatin motion and spatial positioning of gene-specific loci, heterochromatin, and chromatin domains.

**Conclusions:**

Our findings suggest that, while B-type lamin degradation does not significantly change genome topology, it has major implications for three-dimensional chromatin conformation at the single-cell level both at the lamina-associated periphery and the non-LAD-associated nuclear interior with concomitant genome-wide transcriptional changes. This raises intriguing questions about the individual and overlapping roles of lamin B1 and lamin B2 in cellular function and disease.

**Supplementary Information:**

The online version contains supplementary material available at 10.1186/s13059-024-03212-y.

## Background

Chromatin is the complex macromolecular polymer-assembly formed by folding the genome and its associated proteins within the confines of the cell nucleus. Numerous studies indicate that the interplay of physical, chemical, and molecular principles leads to the emergence of the major organizational features of the genome, including polymer–polymer interactions, chromatin loop extrusion [1, 2], phase separation [3, 4], the physical interaction of chromatin with stable architectural elements of the nucleus (i.e., nuclear envelope) [5–7], and chromatin dynamics [8, 9]. Our understanding is limited, however, about the mechanisms governing chromatin folding due to the tethering that occurs at the nuclear periphery.

It is widely believed that the nuclear lamina is the structural limit on the spatial distribution of 3D genome organization in the nucleus [10]. Lamin proteins are structural components of the nuclear lamina that interact with both the cytoskeleton and the genome [11]. Lamins interact with chromatin either indirectly or directly through chromatin-binding proteins [12]. Mechanistically, these lamin proteins are also involved in several critical processes, including transcription, DNA repair, and replication. As a result, lamin dysregulation is associated with more than 15 diseases, termed laminopathies [11, 13, 14]. The four main lamin isoforms found in mammals are lamin A/C, lamin B1, and lamin B2 [11]. Although lamin B1, lamin B2, and lamin A/C form overlapping networks, B-type lamins localize primarily to the nuclear periphery adjacent to the inner nuclear membrane, while A-type lamins can extend into the nucleoplasm [6, 15]. In contrast to A-type lamins, B-type lamins remain tightly associated with the nuclear membrane and have been mapped using DamID to reveal the existence of dynamic and functional euchromatin lamin B1 domains [16]. Prior work identified that inhibition of lamin A/C and lamin B receptor (LBR) alters A/B compartment segregation, specifically chromatin region localization [17, 18]. Differential inhibition of lamin B1 or B2, in contrast, has been shown to have variable effects on compartmentalization. These varied effects are hypothesized to be due to the persistence of the conjugate B-protein [6, 14, 19]. As B-type lamins have similar domain structures, lamin B1 and lamin B2 were conjectured to have redundant functions [20]. It has also been postulated that silencing B-type lamin expression leads to a dramatic increase of lamin A mobility in the nucleoplasm to maintain chromatin organization and transcription [21]. These varied hypotheses illustrate a need to uncover how lamins B1 and B2 contribute to normal cellular physiology together and individually.

Mechanistically, B-type lamins are thought to regulate chromatin structure and gene transcription by physical attachment of chromatin to constrain chromatin dynamics and structure [6]. This mechanism has been evidenced by the recruitment of genes to the nuclear lamina within regions termed lamin-associated domains (LADs), associated with transcriptional repression, B compartments, and heterochromatin nucleosome markers (e.g., H3K9me2/3) [16, 22–24]. Paradoxically, transcriptionally rich territories exist adjacent, and interspersed within, these sites of transcriptional repression, suggesting that the nuclear lamina forms a complex environment for transcriptional regulation [25]. Physically, LADs are variably sized, with domains spanning from 100 kb to 10 Mb and a median size of 0.5 to 1 Mb from ChIP-seq analysis of lamin A/C and lamin B1 [24]. However, the reported relative sizes of LADs can also vary between molecular and imaging techniques or cell type. For example, while imaging studies in mouse and human cells report LADs to be 10 kb to 10 Mb in size, ChIP-seq studies in *Drosophila* cells report LADs to vary between 7 and 700 kb [23, 26, 27].

Despite the broad role of B-type lamins in physiology, the essentiality and redundancy of these proteins pose a formidable challenge to understanding their distinct role in regulating cell function. To overcome this limitation, we implemented the auxin-inducible degron system to degrade both B-type lamins simultaneously (see “ Materials and methods”). Utilizing this engineered cell line and based on prior studies of lamin A/C compared to lamin B receptor in nuclear inversion [17, 18], we tested the hypothesis that the mechanism of action of B-type lamins is due to their role in tethering the genome to the nuclear periphery. In the predominant model of lamin B1/B2 at present, these lamins act by the differential constraint of inner nuclear matrix to regulate chromatin higher-order structure and dynamics [5, 6, 16, 23]. Based on these prior studies, we hypothesized that simultaneous LMNB1 and LMNB2 inhibition would result in (1) increased chromatin chain fluctuations, (2) internal translocation of heterochromatin domains, (3) dissociation of genes from the nuclear periphery, and (4) that the genes normally located on the nuclear periphery would be differentially expressed due to the transformation in mesoscale (100 kb–1 Mb -; ~ 50–200 nm) [28–30] chromatin folding.

Surprisingly, although we observed an increase in chromatin density fluctuations, heterochromatic translocation, and gene loci internalization, mesoscale chromatin folding was minimally altered. Our findings suggest that substantial upregulation and downregulation of genes both within and outside of LADs is a direct consequence of these chromatin dynamics. By integrating chromatin imaging and gene expression data across several length scales, we propose that B-type lamins synergistically contribute to chromatin conformation (i.e., domains defined spatially at the single-cell level) independent of nuclear-scale chromatin regulation (i.e., contact-based domains in a cell ensemble).

## Results

### Addition of the mAID tag to B-type lamins does not impact proper localization of lamin A/C

**Fig. 1:**
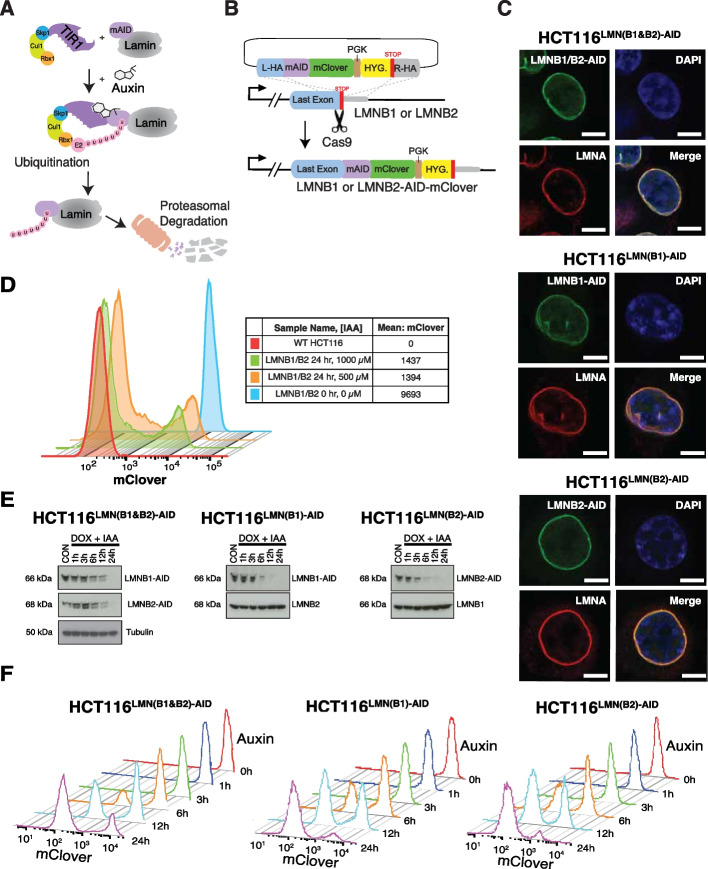
Auxin treatment allows for B-type lamin degradation without affecting Lamin A localization. **A** Schematic illustration showing the AID system. Auxin treatment promotes the interaction between OsTIR1 and the degron tag (mAID), which is fused to the target protein. This results in rapid degradation of the target protein(s) upon proteasomal mediated poly-ubiquitination. **B** Schematic illustration of creating the cell lines. Each gene of interest was targeted for degradation by co-transfecting progenitor cells with the donor template plasmid with Cas9 and sgRNAs targeting the STOP codon of the sequence. **C** Immunostaining of AID-tagged lamin proteins in relation to LMNA. Green: LMNB1/B2-AID, LMNB1-AID, and LMNB2-AID. Red: LMNA. Blue: DAPI staining. The maximum intensity projections of nuclear *Z* stacks are shown. Scale bars = 10 μm. Data are representative of two independent biological replicates (*N* = 2). **D** Flow cytometric analysis to determine the optimal auxin concentration ([IAA]) for maximal degradation of LMNB1 and LMNB2 in fixed HCT116^LMN(B1&B2)−AID^ cells. At least 20,000 events were recorded during the experiment. **E** Western blot analysis shows drastically reduced AID-tagged B-type lamins within 24 h of auxin (IAA) treatment. Doxycycline (DOX) was added 24 h prior to IAA to induce OsTIR1 expression. Tubulin was used as a loading control. Data are representative of three independent biological replicates (*N* = 3). **F** Flow cytometric analysis to determine the optimal auxin treatment time for maximal degradation of LMNB1 and LMNB2 in fixed HCT116^LMN(B1&B2)−AID^, HCT116^LMN(B1)−AID^, and HCT116^LMN(B2)−AID^ cells. At least 20,000 events were recorded during the experiment

Prior attempts at simultaneous elimination of lamin B1 and lamin B2 have not been successful due to their critical roles in cellular viability and other important cellular processes [13]. To overcome this problem, we utilized the auxin-inducible degron system, which allowed simultaneous inhibition of B-type lamins at short timescales [31, 32]. In brief, the auxin-dependent degradation pathway found in plants can be introduced into non-plant eukaryotic species to induce rapid depletion of a protein of interest upon exposure to the phytohormone, auxin. This is achieved by fusing a destabilizing domain (degron) to the protein of interest (Fig. 1A, B). In cells expressing the F-box protein from *Oryza Sativa* (OsTIR1), the addition of auxin results in OsTIR1 forming a functional SCF (Skp1-Cullin-F-box) ubiquitin ligase. Proteins fused with the 7-kDa degron termed mini-AID (mAID) derived from the IAA17 protein of *Arabidopsis thaliana* are rapidly degraded. Importantly, this degradation system is reversible and tunable, allowing for greater control of target protein degradation. To achieve this in B-type lamins, we CRISPR-engineered HCT116 colorectal carcinoma epithelial cells and knocked in the mini auxin-inducible degron and mClover at the end of endogenous lamin B1 (LMNB1) and lamin B2 (LMNB2) gene loci (Fig. 1B; Additional file 1: Table S1-3) [33]. For simplicity, we will hereon refer to these engineered cell lines as HCT116^LMNB1−AID^, HCT116^LMNB2−AID^, and HCT116^LMN(B1&B2)−AID^.

To confirm that addition of the mAID tag does not alter the functionality of the tagged lamin proteins, we first used immunofluorescence to ensure the continued association of lamin B1/B2 with other proteins that localize to the same compartment as the untagged proteins (i.e., lamin A/C). Several studies have demonstrated that subcellular localization is essential for proper protein function and biological processes and that mis-location is strongly associated with human disease [34, 35]. Protein function is determined by subcellular localization due to preferential chemical environments and interaction partners [36]. As expected, our results indicate that LMNB1-mAID-mClover, LMNB2-mAID-mClover, and LMNB1/B2-mAID-mClover proteins localize properly to the nuclear lamina in cells (Fig. 1C). We observed a strong overlap between the mClover signal (hence LMNB1-AID and/ or LMNB2-AID) and Lamin A/C immunostaining signal. This result suggests that addition of the mAID-mClover tag does not alter proper localization or protein function of LMNB1 and LMNB2. To further test cellular functionality, we compared the growth rates of the parental cell line (WT HCT116; WT refers to wild type) and one of the tagged cell lines (HCT116^LMN(B1&B2)−AID^) with and without the addition of doxycycline to induce OsTIR1, as well as with and without the addition of both doxycycline and auxin to degrade B-type lamins (Additional file 2: Fig. S1A). HCT116^LMN(B1&B2)−AID^ treated with both doxycycline and auxin had a nearly identical growth rate as the parental cell line but slowed after about 35 h of treatment. Doxycycline treatment alone resulted in a slightly lower growth rate than that of the parental cell line after about 30 h of treatment as well. Therefore, although the addition of the mAID tag does not alter proper localization of lamins, our findings suggest that B-type lamins play a role in cell proliferation.

### Auxin treatment results in rapid and reversible depletion of targeted B-type lamins

We next investigated the kinetics of mAID-tagged lamin degradation to confirm the suitability of this approach for reversible inhibition of B-type lamins. A major advantage of the AID system is that conditional depletion of target proteins has less off-target effects than traditional perturbation methods such as RNA interference [31]. This allows examination of resulting changes in chromatin structure and transcription at the most relevant time scales, in which these changes are directly associated with target protein degradation. Further, reversal of target degradation can be achieved by simply removing auxin from cell culture media. We first evaluated the impact of auxin treatment on B-type lamin degradation. To induce the expression of OsTIR1, we added 2 mg/mL doxycycline to cell media 24 h prior to auxin treatment. Western blots of global protein levels and fluorescent-activated cell sorting (FACS) indicated that 1000 µM auxin treatment resulted in rapid degradation of AID-tagged lamins in HCT116^LMNB1−AID^, HCT116^LMNB2−AID^, and HCT116^LMN(B1&B2)−AID^ cells (Fig. 1D–F). Based on western blot results, nearly 80% of LMNB1-mAID and LMNB2-mAID was degraded within the first 6 h of auxin treatment. Simultaneous depletion of both lamins was achieved by 24 h of treatment, although flow cytometry results indicated that low levels of LMNB1-mAID and LMNB2-mAID remained in the HCT116^LMN(B1&B2)−AID^ cells. From these results, we confirmed the utility of this mAID system as a means for substantial degradation of endogenous LMNB1 and LMNB2 proteins to subsequently investigate their role in chromatin folding, dynamics, and cellular function.

### Acute depletion of B-type lamins alters cell cycling and nuclear morphology

**Fig. 2:**
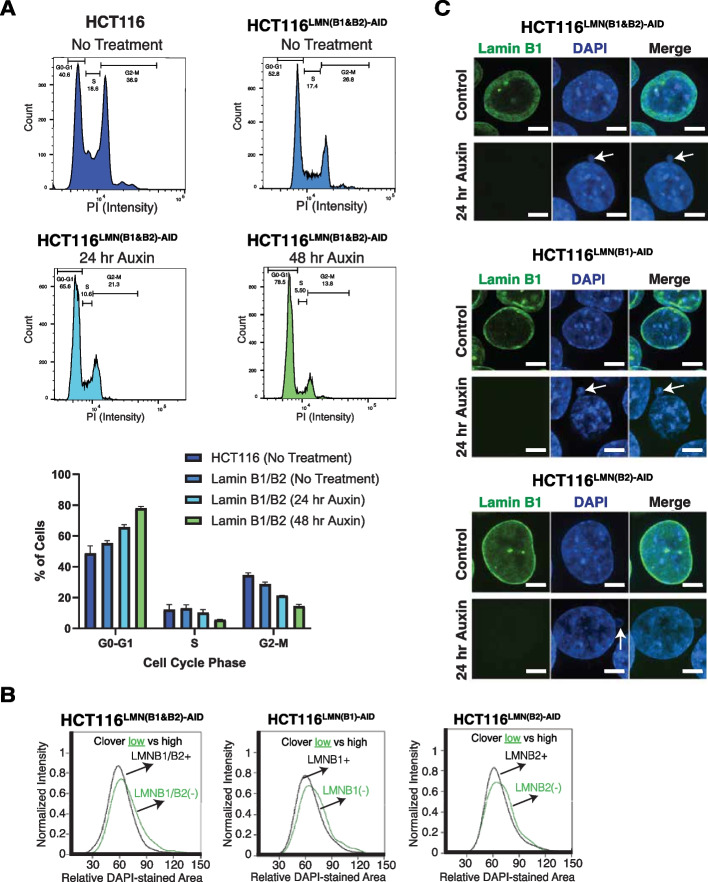
Loss of B-type lamins induces cell cycle arrest and altered nuclear morphology. **A** Propidium iodine staining in HCT116 and HCT116.^LMN(B1&B2)−AID^ cells to assess the percentages of cells in G1, S, and G2/M phase. Data are representative of three independent biological replicates (*N* = 3). **B** Flow cytometric analysis to measure relative DAPI-stained nuclear area from data collected with the ImageStreamX after 8 h of auxin treatment. Black: auxin-treated cells containing high mClover intensity. Green: auxin-treated cells with low or no mClover intensity. At least 20,000 events were recorded during the experiment. **C** Immunofluorescence shows the presence of nuclear blebbing in B-type lamin-deficient cells. Scale bar = 5 μm. Data are representative of three independent biological replicates (*N* = 3)

To further investigate the role of B-type lamins in retaining cell function and assess the impacts of a slowed growth rate, we applied flow cytometry-based cell cycle analysis using propidium iodide staining (Fig. 2A). Our results indicate that the untreated HCT116^LMN(B1&B2)−AID^ cells were stalled in the G0–G1 phase in comparison to the untreated parental cells. The HCT116^LMN(B1&B2)−AID^ cells became arrested at the G0–G1 phase even more so when B-type lamins were degraded at 24 and 72 h of auxin treatment. Subsequently, there were less HCT116^LMN(B1&B2)−AID^ cells within the S phase and G2-M phase at 48 h of auxin treatment. As the nuclear envelope is thought to function in the confinement of the genome, we next analyzed the effect of B-type lamin inhibition on nuclear morphology. As expected, we observed morphological alterations when either of the lamins was depleted. To quantify these effects, we used ImageStreamX, a flow cytometry-based high-throughput microscopy imaging platform. At the 8-h auxin treatment time point for all three cell lines, the population of cells included both mClover-positive (intact lamins) and mClover-negative (degraded lamins) cells. Using this flow cytometry-based analysis of DAPI-stained nuclear area, we found that mClover-negative cells had slightly enlarged nuclear area in all three tagged cell lines (Fig. 2B). We verified this small effect size with fixed-cell immunofluorescence imaging by comparing the untreated and 24-h auxin-treated cells, and also found that the mAID-tagged cells appeared to be smaller than the parental cells (Additional file 2: Fig. S1B). Additionally, we noticed a notable increase in the presence of nuclear deformations, such as nuclear blebbing (Fig. 2C). To evaluate if acute depletion of B-type lamins transformed nuclear morphology due to secondary effects on cell viability, we used FACS to analyze cell death in HCT116^LMNB1−AID^, HCT116^LMNB2−AID^, and HCT116^LMN(B1&B2)−AID^ cells. Using fluorescently labeled Annexin V (Annexin V^APC^) and DAPI staining to measure overall apoptotic and necrotic cell death [37], we confirmed that the reduction of both B-type lamins did not induce notable apoptosis or necrosis at 12, 24, or 48 h of auxin treatment in comparison to the untreated conditions (Additional file 2: Fig. S1C) As such, this indicated that the alterations in nuclear morphology and slowed cell cycle progression were direct consequences of B-type lamin removal and that subsequent effects explored were not secondarily due to processes associated with cellular death.

### Acute depletion of B-type lamins induces minimal changes in mesoscale organization at the level of TADs and chromatin contact scaling

Owing to the macroscopic alterations in cell cycling and nuclear morphology observed above as well as prior work showing the role of lamin A/C and LBR in mesoscale chromatin organization [17, 18], we hypothesized that the degradation of both B-type lamins would produce global alterations in chromatin folding, topologically associated domains (TADs), loops, and contact scaling. To test this, we performed in situ Hi-C [25], which can capture structural changes in the kilobase-level DNA-DNA contacts [2], in the AID cell lines (HCT116^LMNB1−AID^, HCT116^LMNB2−AID^, and HCT116^LMN(B1&B2)−AID^ cells). For each cell type, we generated more than a 1 billion contacts from cells before (~ 1,354,831,021) and after 24 h of auxin treatment (~ 1,210,053,449). At least two biological Hi-C replicates for all three cell lines were obtained.

**Fig. 3:**
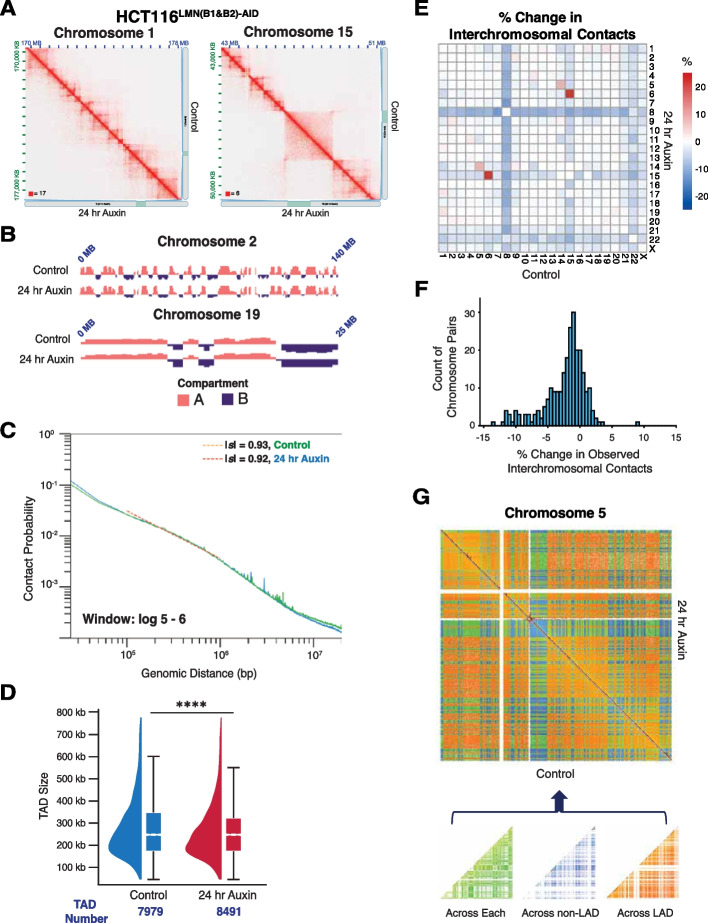
Mesoscale chromatin structure is overall preserved upon B-type lamin degradation. **A** Representative normalized Hi-C trans-interaction matrices for chromosomes 1 and 15 in the control and 24-h auxin treatment conditions are shown for HCT116^LMN(B1&B2)−AID^ cells. 15 kb resolution. **B** The eigenvectors for chromosomes 2 and 19 located at the nuclear periphery and interior, respectively, are shown. Pink: A compartment. Purple: B compartment. Eigenvectors computed by Juicer are the first eigenvector of the correlation matrix of the binned Hi-C contacts. **C** Contact probability scaling for HCT116^LMN(B1&B2)−AID^ cells are shown for the control and 24-h auxin treatment conditions. Absolute values of *s* are indicated. **D** Raindrop plot comparing TAD sizes for the control and 24-h treatment conditions as revealed by TopDom. The number of TADs for each condition is indicated below the plot. Error bars: SEM. Significance was calculated by paired *t* test (**** < 0.0001). **E** Normalized Hi-C trans-interaction matrix demonstrating proximity of chromosome territories. The heatmap scale indicates the % change in interchromosomal contacts before and after 24-h auxin treatment in HCT116^LMN(B1&B2)−AID^ cells. **F** Histogram demonstrating % change in interchromosomal contacts before and after 24-h auxin treatment in HCT116.^LMN(B1&B2)−AID^ cells. **G** The Hi-C trans-interaction matrix demonstrates contacts across LAD segments, non-LAD segments and contacts across both segments for chromosome 5. The schematic under the matrix shows the partitioning of contacts across LAD segments and non-LAD segments for a representative chromosome. For **A–G**: Data was pooled from two independent biological replicates (*N* = 2)

Multiple methods have been proposed for quantifying the mesoscale organization of the genome based on Hi-C data, including (1) analysis of A/B compartment switching [38], (2) eigenvector decomposition [25], (3) measurement of contact scaling *(|s|)* [39], and (4) evaluation of TAD stability [40–42]. Using Hi-C data, the relationship between chromatin packing behavior and genome connectivity can be conceptualized by the contact probability scaling exponent (*s*). The probability (*P*) of contact between two monomers separated by length (*N*) along a linear chromatin chain follows a power-law scaling relationship: *P µ N*^*−s*^ [39]. Notably, upon reduction of either or both lamin B1 and lamin B2, we observed minimal changes in mesoscale chromatin structure (Fig. 3A; Additional file 2: Fig. S2A-C). Degradation of both B-type lamins at 24 h also did not result in major weakening or switching of chromosomal A/B compartments based on Eigenvectors (Fig. 3B) nor change the frequency of contacts as measured by *|s|* (Fig. 3C). The frequency and size of TADs also remained relatively the same, although chromosomes 9 and 21 had more substantial changes in TAD sizes (Fig. 3D; Additional file 2: Fig. S2D). For example, using TopDom [43] analysis, a domain-calling algorithm, we found that the mean TAD size slightly decreased (control ~ 348 kb versus 24 h Auxin ~ 326 kb) while the number of TADs only modestly increased upon 24 h of auxin treatment (control 7979; 24 h Auxin 8491) (Fig. 3D). This is in major contrast to the findings observed in LBR depletion where compartment switching and TADs were markedly transformed in thymocytes lacking LBR compared to WT controls [17]. We did however find that B-type lamin perturbation led to a decrease in trans-chromosomal interactions, with chromosome-specific differences (Fig. 3E, F). While the number of contacts increased between a few chromosomes, the mean contact frequency between most chromosomes was substantially reduced, suggesting the redistribution and expansion of chromosome territories (Fig. 3F; Additional file 2: Fig. S2E).

Given these findings, we hypothesized that the limited changes in overall mesoscale chromatin structure upon joint lamin B1 and lamin B2 inhibition could be limited to local alterations in TADs and contact scaling within LADs. To perform this segmental analysis, we utilized publicly available DNA adenine methyltransferase identification (DamID) data for Lamin B1 in HCT-116 cells [44, 45]. We computationally segmented the genome into contacts that spanned both LADs and non-LADs (across each), across only LADs, or across only non-LADs (Fig. 3G). Visually, we did not detect any changes in the Hi-C contact maps within segments that were spanning only LADs, only non-LADs, or spanning each. To quantify the effect of B-type lamin depletion on chromatin organization, we next calculated the effect of their depletion on scaling within these different segments. As prior work has shown that LADs are associated with dense B compartments [23, 24], we hypothesized that contact scaling would decay slower within LADs compared to non-LAD segments.

**Fig. 4:**
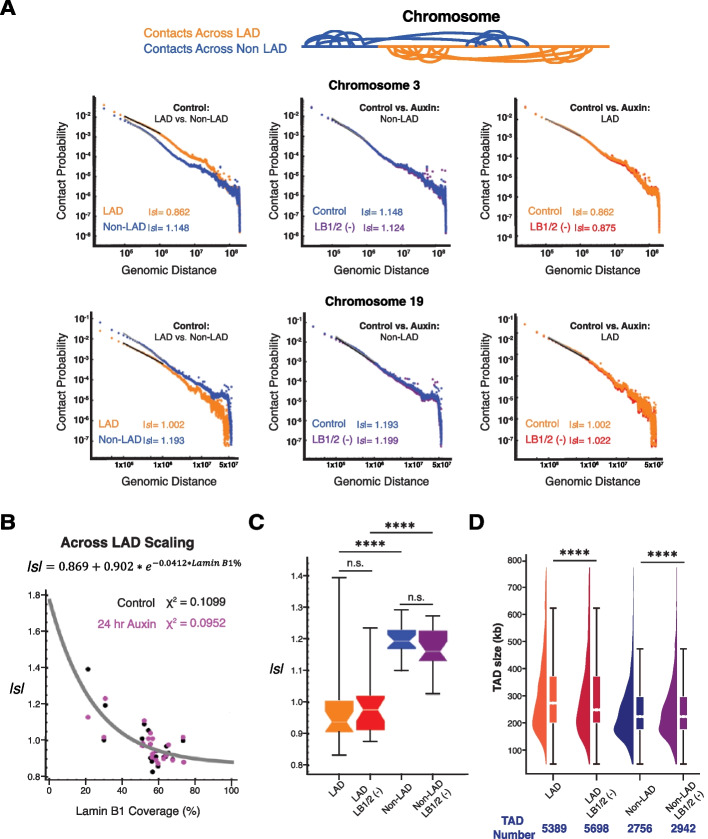
Loss of B-type lamins has a negligible impact on genome connectivity. **A** Contact scaling for chromosomes 3 and 19 located at the nuclear periphery and nuclear interior, respectively, is shown. The left plots indicate contact scaling for each chromosome in the untreated condition to compare LAD and non-LAD segments. The middle and right plots indicate contact scaling for each chromosome to compare the control (untreated) and 24-h auxin treatment conditions in HCT116.^LMN(B1&B2)−AID^ cells. Absolute values of *s* are indicated. **B** Scatter plot of the inverse relationship between lamin B1 coverage and *|s|* in LAD segments. The exponential distribution (gray curve) is fitted to the merged control (black) and 24-h auxin treatment condition (purple) samples. **C** Contact scaling within LADs and non-LADs for both the control (untreated) and 24-h auxin treatment conditions. Error bars: SEM. The line within each box represents the median; the outer edges of the box are the 25th and 75th percentiles and the whiskers extend to the minimum and maximum values. Significance of the differences in observed frequencies was calculated by unpaired *t* test (n.s. = not significant; **** < 0.0001). **D** Raindrop plot comparing TAD sizes for the control and 24-h treatment conditions as revealed by TopDom. The number of TADs for each condition/ segment is indicated below the plot. Error bars: SEM. Significance was calculated by paired *t* test (**** < 0.0001). For **A–D**, data was pooled from two independent biological replicates (*N* = 2)

Consistent with this hypothesis, in the control conditions, we observed a lower *|s|* in both a chromosome associated with the periphery (chromosome 3; *|s|* 0.862 vs 1.148 for LAD vs non-LAD) and in the center (chromosome 19, *|s|* 1.002 vs 1.193 for LAD vs non-LAD) in the LAD segments compared to the non-LAD control (Fig. 4A). However, despite these differences at baseline, there was no substantial change in contact probability scaling between the control and 24-h auxin conditions either within LAD domains or within non-LAD domains for either the peripherally associated or centrally associated chromosome.

Further, analysis of each chromosome demonstrated an inverse relationship between *|s|* within LADs and the percent coverage of the chromosome in contact with lamin B1 (Fig. 4B). However, despite the large difference in *|s|* at baseline (Fig. 4A) and inverse relationship between *|s|* and lamin B1 coverage, only minor changes in *|s|* were observed upon B-type lamin depletion with a larger magnitude of effect observed within the non-LAD portions (Fig. 4C). As expected, we observed a lower *|s|* (calculated between 10^5^–10^6^ bp) within LADs compared to non-LADs in untreated cells (Fig. 4C). This is because a chromatin polymer with a slow rate of decay of chromatin volume concentration as a function of distance from the center of a domain (i.e., chromatin within a LAD) should be associated with a higher contact frequency among distant loci [39]. This translates into a lower contact probability scaling *|s|.* As expected, there was no relationship between *|s|* within non-LADs and the chromosomal coverage of lamin B1 (Fig. S2F). This finding was similarly extended into analysis of TADs. Analysis of TAD size and number confirmed that the mean size of TADs within LAD regions is higher than that of TADs outside of LAD regions (LADs control ~ 371 kb versus 24 h Auxin ~ 419 kb; non-LADs control ~ 241 kb versus 24 h Auxin ~ 254 kb) (Fig. 4D). Overall, these findings indicated that although genomic segments that associate with B-type lamins have distinct organization compared to non-LAD segments at the level of their polymeric interactions and TAD structures, the disruption of lamin B1/B2 surprisingly did not meaningfully transform these properties. In the context that low *|s|*, larger TADs would be similar to denser/ highly packing chromatin such as heterochromatin, this raised two interesting scenarios: (1) B-type lamins produce heterochromatin-like structures that, once formed, are not-reversed on its loss or (2) that heterochromatin-like structures preferentially interact with B-lamins but are not exclusively maintained by them. We tested these competing hypotheses with a combination of chromosomal paint, live-cell Dual-PWS microscopy, CRISPR-Sirius, FISH, super-resolution microscopy, and RNA sequencing (RNA-seq) in the following sections.

### Lamin B1 and B2 depletion promotes chromatin spatial re-localization at the nuclear periphery

**Fig. 5:**
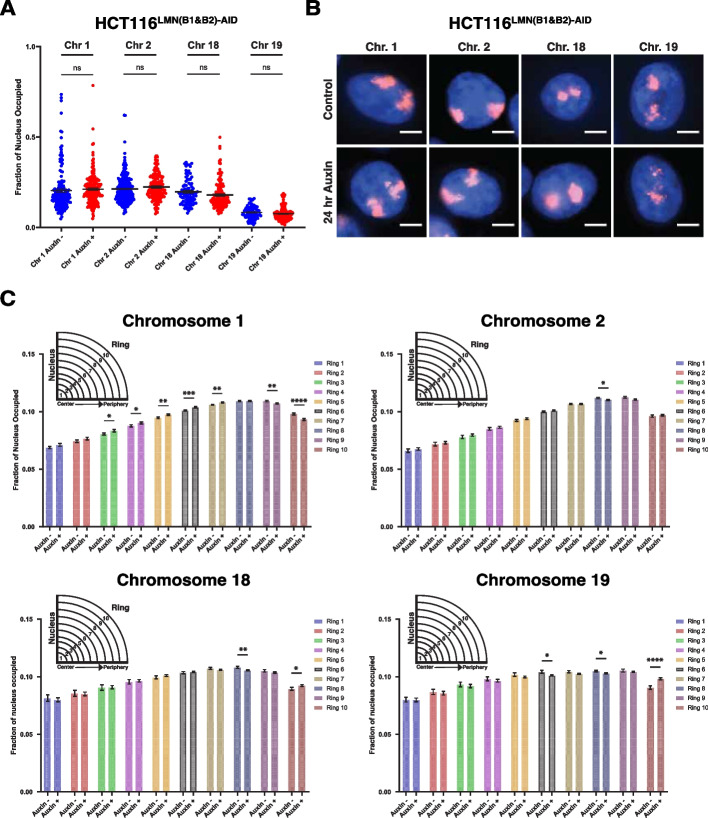
Reduced levels of B-type lamins increases chromatin mobility at the nuclear periphery. **A** Scatterplot of nuclear fraction occupied by chromosomes 1, 2, 18, and 19 before and after 24-h auxin treatment. Error bars: SEM. Data was compiled from three independent biological replicates (*N* = 3). Significance was calculated by unpaired *t* test (n.s. = not significant). (Chr. 1 (Control *n* = 165; Auxin *n* = 195), Chr. 2 (Control *n* = 203; Auxin *n* = 174), Chr. 18 (Control *n* = 89; Auxin *n* = 148), Chr. 19 (Control *n* = 120; Auxin *n* = 83)). The violin plots extend from the minimum to the maximum value. The line in the middle of each plot is the median value of the distribution, and the lines above and below are the third and first quartiles, respectively. Each dot represents one cell. **B** Representative immunofluorescence images of HCT116.^LMN(B1&B2)−AID^ cells visualized through the hybridization of chromosome paints. Data are representative of two independent biological replicates (*N* = 2). **C** Graphing of the average fraction and SEM of each chromosome fluorescent signal present in each one of ten concentric nuclear rings (ring 1: most central, ring 10: most peripheral). Data was compiled from two independent biological replicates (*N* = 2). Significance was calculated by unpaired *t* test (* < 0.05; ** < 0.01; *** < 0.001; **** < 0.0001). (Chr. 1 (Control *n* = 165; Auxin *n* = 195), Chr. 2 (Control *n* = 203; Auxin *n* = 174), Chr. 18 (Control *n* = 89; Auxin *n* = 148), Chr. 19 (Control *n* = 120; Auxin *n* = 83))

To investigate the observed mechanism behind minimal changes in higher-order chromatin folding structure, we performed two biological replicates of chromosome painting for chromosomes 1, 2, 18, and 19. We hypothesized that if B-type lamin depletion induced a shift in chromatin spatial localization at the chromosome level, then scenario (2) was more likely as it indicated an active interaction between the lamins and position maintenance. Chromosomes 1 and 2 were chosen to represent chromosomes localized relatively near the nuclear periphery while chromosomes 18 and 19 were chosen to represent chromosomes with preferential localization at the nuclear interior. We first measured the fraction of nuclei covered by each chromosome (Fig. 5A, B). Our results indicate that there is no significant change in chromosomal area for each of the chromosomes.

To further explore the mechanistic role of B-type lamins in stabilization of chromosomal positioning along the nuclear periphery, we measured the distribution of each chromosome by partitioning each nucleus into 10 bins of equal radius. To account for differences in area for each ring, we normalized these values using the equation 2k-1, where *k* is the ring number. Our results verified that chromosomes 1 and 2 are preferentially localized to the nuclear periphery, whereas chromosomes 18 and 19 are localized more internally and spread more equally among the rings (Fig. 5B, C). Importantly, chromosome 1 had a clear decrease in occupancy at the nuclear periphery upon B-type lamin removal (i.e., rings 9–10), with a resulting increase in this occupancy towards the nuclear interior (i.e., rings 3–7) (Fig. 5C; Additional file 2: Fig. S3A). Chromosome 2 occupancy was slightly reduced at ring 8, while the occupancy of chromosomes 18 and 19 remained primarily at the nuclear interior despite an increase at ring 10. This increased fraction of internal chromosomes appearing in the outmost ring could suggest a more accessible chromatin state at the nuclear periphery.

To determine if there was a difference in the chromosome occupancy of nuclei between the control and auxin conditions, we then compiled all 10 rings (Additional file 2: Fig. S3B). Our results indicated no significant difference in the means between conditions. We also calculated the coefficient of variation (CoV) for each condition and chromosome, which is obtained by dividing the standard deviation by the mean and is expressed as a percentage. Our results demonstrated a lower sample variability in the auxin-treated condition for all samples, indicating that when B-type lamins are disrupted, chromosomes tend to have a more homogenous spread throughout the nuclear area. Overall, this physical expansion of chromosome territories paired with the observed minimal changes at the nuclear scale further suggests that B-type lamins regulate chromatin subcellular localization using a motion-dependent mechanism.

### Disruption of B-type lamins alters chromatin dynamics but has a minimal effect on higher-order structure in live cells

Although we did not observe major alterations to chromatin folding by in situ Hi-C, the re-localization of chromosomes at the nuclear periphery led us to hypothesize that disruption of B-type lamins could impart a differential effect on cellular function by changing chromatin dynamics in live cells. To test this hypothesis, we used dual-mode live-cell Partial Wave Spectroscopic (PWS) microscopy to detect both chromatin structural changes and chromatin mobility variations. PWS microscopy provides label-free measurements of nanoscale structural changes with a sensitivity to structures between ~ 20 and 200 nm in live cells without the use of cytotoxic labels [46–48]. Briefly, in dual-mode PWS microscopy, variations in nanoscopic-macromolecular structure are measured by analyzing the spectral dependence in light scattering from the packing of chromatin into higher-order structures, such as chromatin packing domains, while temporal variations in scattering allow measurement of chromatin mobility [39, 49–51]. These domains are characterized by polymeric fractal-like behavior, high chromatin packing density, and a radial decrease in mass density from the center to the periphery [29, 52]. As the primary macromolecular assembly within the nucleus is chromatin, this method has been shown to detect changes in chromatin folding comparable to Hi-C and electron microscopy [39]. Given that higher-order chromatin structure is determined by the polymeric folding across length scales between 10 and 200 nm and is approximately a power-law (quantified by *|s|* and is observed in multiple polymer models), this distribution in packing corresponds to the distribution of the refractive index quantified by mass scaling (chromatin packing scaling,* D*) [52]. This scaling parameter is calculated for each pixel within the region of interest (i.e., nucleus) within a given coherence volume, which is determined by the depth of field longitudinally and the axial plane for each pixel. Therefore, spatial variations of macromolecular density and motion that can occur upon perturbation, such as degradation of B-type lamins, can be measured using PWS.

**Fig. 6:**
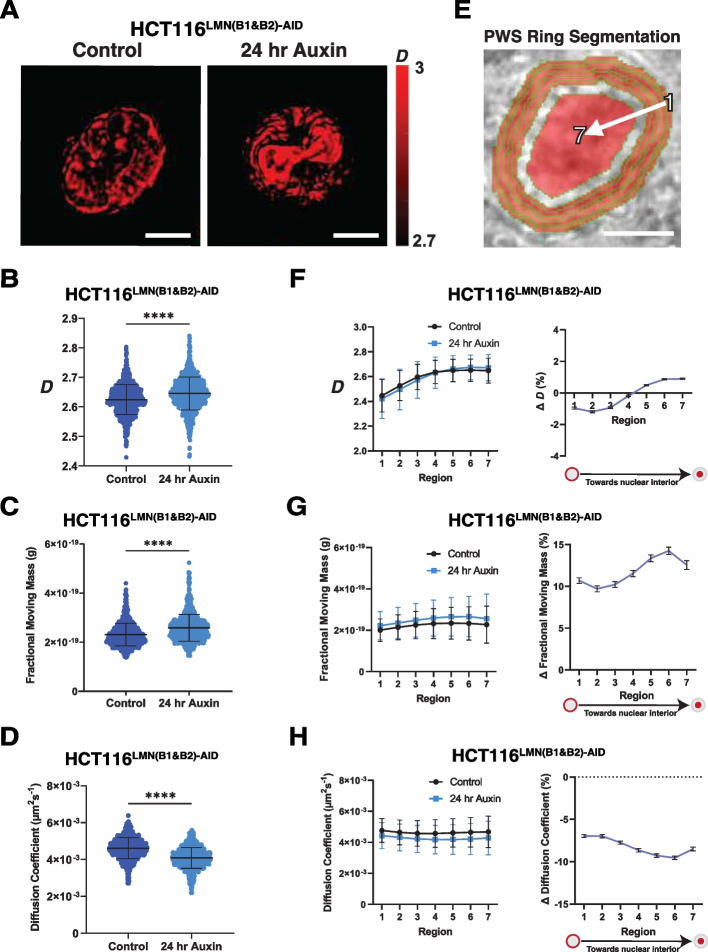
Dual-PWS reveals differential chromatin packing scaling and dynamics upon B-type lamin degradation. **A** Representative PWS images of HCT116^LMN(B1&B2)−AID^ cells before and after 24-h auxin treatment. Scale bar = 5 μm. Data are representative of three independent biological replicates (*N* = 3). **B** Violin plots for chromatin packing scaling (*D*) in HCT116^LMN(B1&B2)−AID^ cells before and after 24-h auxin treatment. **C** Violin plots for fractional moving mass in HCT116^LMN(B1&B2)−AID^ cells before and after 24-h auxin treatment. **D** Violin plots for diffusion coefficient in HCT116^LMN(B1&B2)−AID^ cells before and after 24-h auxin treatment. **E** Segmentation for PWS regional analysis. Region 1 is the nuclear periphery while region 7 is the nuclear interior. Scale bar = 5 μm. **F** Regional PWS measurements and percent changes of *D* in HCT116^LMN(B1&B2)−AID^ cells before and after 24-h auxin treatment. Error bars: SD. **G** Regional PWS measurements and percent changes of fractional moving mass in HCT116^LMN(B1&B2)−AID^ cells before and after 24-h auxin treatment. Error bars: SD. **H** Regional PWS measurements and percent changes of the diffusion coefficient in HCT116^LMN(B1&B2)−AID^ cells before and after 24-h auxin treatment. Error bars: SD. For **B–H**: Data was compiled from three independent biological replicates (*N* = 3). Significance was calculated by unpaired *t* test with Welch’s correction applied (**** < 0.0001). (Control *n* = 953; Auxin *n* = 869). The truncated violin plots in **B–D** extend from the minimum to the maximum value. The line in the middle of each plot is the median value of the distribution, and the lines above and below are the third and first quartiles, respectively. Each dot represents one cell

As *|s|* and *D* are inversely related based on prior experimental studies and polymer modeling [39, 51], we hypothesized that that nuclear average *D* would increase slightly after inhibition of lamin B1 and lamin B2 due to the small decrease in *|s|* observed within non-LAD portions of the genome [39]. Upon the addition of auxin to HCT116^LMN(B1&B2)−AID^ cells, the average *D* increased as expected from 2.624 to 2.645 (Fig. 6A, B) with larger but still modest changes observed within HCT116^LMNB1−AID^ (2.563 to 2.628) and HCT116^LMNB2−AID^ (2.627 to 2.706) (Additional file 2: Fig. S4A-B). To confirm this result, we verified a strong anti-correlation between the change in *D* and mClover signal with a 24-h auxin treatment time course and confirmed that OsTIR1 expression induced by doxycycline treatment does not alter *D* (Additional file 2: Fig. S4C-D). Overall, the small increase in* D* and decrease in *|s|* upon 24 h of auxin treatment suggest that the regulatory role of B-type lamins may be independent of mesoscale chromatin folding. We hypothesized that altered chromatin dynamics could be the driving force behind conformational changes (i.e., redistribution of spatially defined domains at the single-cell level).

Utilizing the temporal capabilities of dual-PWS, we then evaluated the influence of B-type lamin degradation of chromatin dynamics [49, 50]. Although dual-PWS lacks molecular specificity, variations in temporal interference from macromolecular movement allow quantification of the fractional moving mass (the magnitude of chromatin evolving in time) and the diffusion coefficient without labels. Fractional moving mass, calculated from the product of the mass of the moving macromolecular cluster and the volume fraction of moving mass, quantifies the physical and dynamic properties chromatin, as not all motion within the cell is specifically diffusive [49]. Unexpectedly, although minor changes in higher-order chromatin structure were observed upon lamin B1/B2 depletion, there were much larger changes in the fractional moving mass on treatment with auxin in HCT116^LMN(B1&B2)−AID^ cells (18.4% increase) (Fig. 6C). Similar changes were observed in HCT116^LMNB1−AID^ and HCT116^LMNB2−AID^ cells (Additional file 2: Fig. S4E). These results suggest that upon lamin B1 and lamin B2 degradation, there are decreased constraints on chromatin occurring within the nucleus [49]. Indeed, we further observed that inhibition of lamin B1 and lamin B2 resulted in a decrease in the ensemble diffusion coefficient of 11.4% compared to the untreated control (Fig. 6D), with similar findings in HCT116^LMNB1−AID^ and HCT116^LMNB2−AID^ (Additional file 2: Fig. S4F). Taken together, our temporal analysis indicates that B-type lamin degradation results in overall greater magnitude of chromatin chain fluctuations with a slower rate of motion. Such a phenotype would be observed with the dissociation of large structures from a confined state in a crowded environment. Although Dual-PWS is not a molecularly specific technique, this result could be explained by the weakening of chromatin and nuclear interactions at the nuclear periphery. These findings therefore provide further evidence that increased chromatin mobility due to a loss of B-type lamins at the periphery induces conformational changes, even in the nuclear interior.

### Lamins B1 and B2 mechanistically determine chromatin mobility and heterochromatin localization throughout the nucleus

Based on our observations above on the slight differential effect in *|s|* in LAD vs non-LADs, the internal shift of peripheral chromosomes, and the increased average chromatin mobility throughout the nuclear area upon reduced B-type lamin expression, we next tested the contribution of B-type lamins on the stabilization of chromatin domains to the nuclear periphery. To distinguish between whole-nuclei and regional chromatin structure, we segmented the nucleus into regions of equal size spanning the nuclear edge and the center of the nuclear interior (Fig. 6E). This spatial analysis involved segmenting the nucleus into seven non-overlapping ribbons to evaluate variations in the location of chromatin packing domains, with 6 ribbons at the nuclear periphery and a large ribbon at the nuclear center. Structurally, *D* near the nuclear periphery was lower than that of the nuclear interior, with minimal differences observed between the periphery and the interior during lamin B1 lamin B2 inhibition (Fig. 6F), and similar trends observed in lamin B1 and B2 inhibition separately (Additional file 2: Fig. S4G). Further, *D* was strongly correlated with the distance from the periphery (Pearson correlation coefficient = 0.95). This differential spatial response of the change in *D* could potentially be explained by the movement of chromatin domains between the periphery and nuclear interior. Unexpectedly, although fractional moving mass was increased and diffusion was decreased throughout the nucleus, the differences between the nuclear periphery and the center in HCT116^LMN(B1&B2)−AID^ cells was minimal (Fig. 6G, H), with comparable results observed in HCT116^LMNB1−AID^ and HCT116^LMNB2−AID^ (Additional file 2: Fig. S4H-I).

**Fig. 7:**
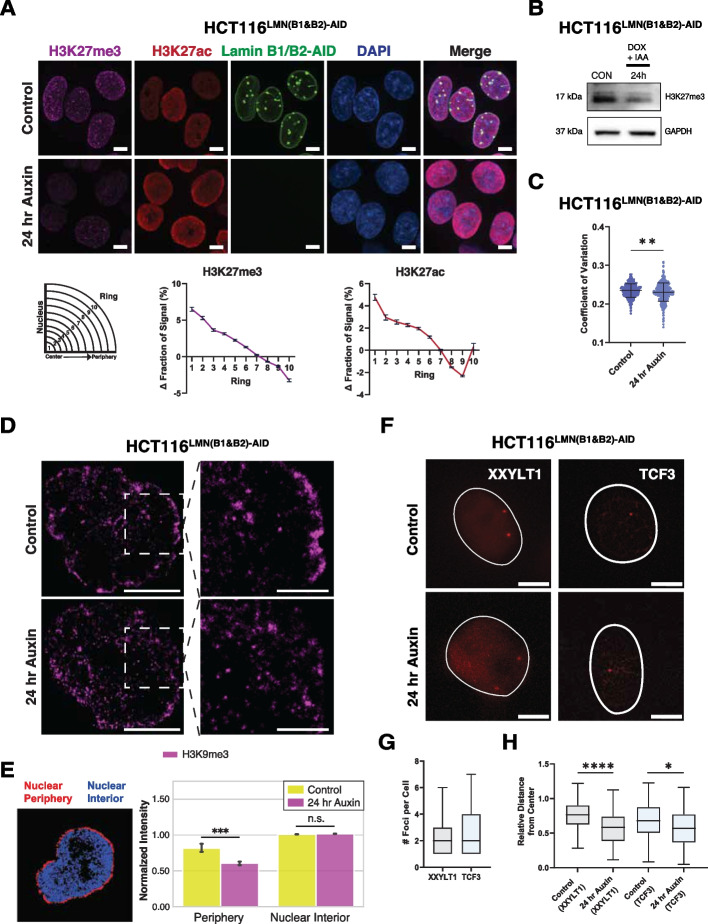
Chromatin decompaction promotes redistribution of heterochromatin and gene loci upon auxin treatment. **A** Immunostaining of H3K27me3 and H3K27ac in HCT116^LMN(B1&B2)−AID^ cells before and after 24-h auxin treatment. Graphing of the average fraction and SEM of fluorescent signal present in each one of ten concentric nuclear rings is shown below. Magenta: H3K27me3. Red: H3K27ac. Green: Lamin B1/B2-AID. Blue: DAPI staining. Maximum intensity projections of nuclear Z stacks and % changes between concentric rings are shown. Scale bars = 5 μm. Error bars: SD. Data are representative of two independent biological replicates (*N* = 2). (H3K27me3 (Control *n* = 455; Auxin *n* = 510), H3K27ac (Control *n* = 448; Auxin *n* = 447)). **B** Western blot analysis of H3K27me3 in HCT116^LMN(B1&B2)−AID^ cells. Doxycycline (DOX) was added 24 h prior to IAA to induce OsTIR1 expression. GAPDH was used as a loading control. Data are representative of three independent biological replicates (*N* = 3). **C** Coefficient of variation plot for HCT116^LMN(B1&B2)−AID^ cells showing chromatin decompaction upon 24 h of auxin treatment. Data was compiled from three independent biological replicates (*N* = 3). Significance was calculated by unpaired *t* test (**** < 0.0001). (Control *n* = 798; Auxin *n* = 845). Violin plots extend from the minimum to the maximum value. The line in the middle of each plot is the median value of the distribution, and the lines above and below are the third and first quartiles, respectively. Each dot represents one cell. **D** STORM images of HCT116^LMN(B1&B2)−AID^ cells before and after 24-h auxin treatment with zoomed-in views of the nuclear periphery. Magenta: H3K9me3. Scale bars = 5 µm, Scale bars (zoom) = 2 µm. **E** Quantification of Normalized STORM Intensity (NSI) with a representative segmentation for the nuclear periphery (red) and nuclear interior (blue). Error bars: SD. Significance was calculated by unpaired *t* test (*** < 0.001). (Control *n* = 5; Auxin *n* = 5). **F** Representative CRISPR-Sirius images of gene loci *XXYLT1* and *TCF3* in live HCT116^LMN(B1&B2)−AID^ cells. Scale bar = 5 μm. Data are representative of two independent biological replicates (*N* = 2). **G** Boxplot showing the number of foci per cell for XXYLT1 and TCF3 loci (XXYLT1 *n* = 42; *TCF3 n* = 25). **H** Box plot showing distances of *XXYLT1* and *TCF3* foci to the nuclear center. Significance was calculated by Mann–Whitney test (* < 0.05; **** < 0.0001) (*XXYLT1* (Control *n* = 115; Auxin *n* = 65), *TCF3* (Control *n* = 116; Auxin *n* = 32)). For **G,H**, data was compiled from two independent biological replicates (*N* = 2). The line within each box represents the mean; the outer edges of the box are the 25th and 75th percentiles and the whiskers extend to the minimum and maximum values

Overall, these findings further suggest a mechanistic role of lamin B1 and lamin B2 in the confinement of chromatin mobility throughout the cell nucleus. Our interpretation of these results is that when lamin B1 and lamin B2 are degraded, chromatin domains slowly move away from the nuclear periphery with a resulting shift towards the nuclear interior. We reasoned that if LMNB1 and LMNB2 depletion leads to the detachment of chromatin domains from the nuclear periphery, as indicated by our dual-mode live-cell regional PWS results, this internal shift would be accompanied by heterochromatin redistribution. Using immunofluorescence, we found a global reduction in H3K27me3 and increase in H3K27ac upon 24 h of auxin treatment in HCT116^LMN(B1&B2)−AID^ cells, with comparable results observed in HCT116^LMNB1−AID^ and HCT116^LMNB2−AID^ (Additional file 2: Fig. S5A-C). Next, we confirmed an internal shift of H3K27me3 from the nuclear periphery using the same segmentation and area normalization for chromosome paint (Fig. 7A; Additional file 2: Fig. S5D). We also noticed that while H3K27ac increased slightly at the nuclear periphery in the outermost ring, in line with chromosomes 18 and 19 from chromosome painting experiments, there was an internal shift as well. To confirm that global histone methylation was reduced, we used western blot analysis to measure the amount of H3K27me3 in triplicate (Fig. 7B; Additional file 2: Fig. S5E; Additional file 3). This analysis revealed a ~ 41% decrease in H3K27me3 upon 24 h of auxin treatment to HCT116^LMN(B1&B2)−AID^ cells. To assess if the observed changes in chromatin dynamics could be further explained by chromatin decompaction, we used spinning disk confocal microscopy to measure the CoV in DAPI-stained HCT116^LMN(B1&B2)−AID^ cells. Previously used to assess the degree of heterogeneity of DNA signal across the nucleus [53], the CoV is calculated as the standard deviation of the DAPI intensity values divided by the mean value of nuclear pixel intensity for each nucleus. Our results demonstrated that upon auxin treatment, chromatin compaction slightly decreased in HCT116^LMNB1−AID^ (1.11%), HCT116^LMNB2−AID^ (2.31%), and HCT116^LMN(B1&B2)−AID^ (2.86%) cells (Fig. 7C, Additional file 2: S5F-G). Therefore, the removal of B-type lamins results in destabilization, re-localization, and a global reduction of heterochromatin that is in part mediated by chromatin decompaction.

To confirm the reduction of heterochromatin-associated domains specifically at the nuclear periphery, we utilized single-molecule localization microscopy (SMLM) to measure the intensity of H3K9me3 in HCT116^LMN(B1&B2)−AID^ cells (Fig. 7D). This powerful imaging technique dramatically improves spatial resolution in comparison to standard, diffraction-limited techniques and provides molecular-specific information [54]. By utilizing STORM, we quantified H3K9me3 intensity normalized to the total nuclear intensity. As LADs are typically enriched in H3K9me3, we selected this marker to represent LAD distribution specifically at the nuclear periphery [16, 24]. We first partitioned each reconstructed image into two regions spanning the nuclear periphery and interior (Additional file 2: Fig. S6A) to quantify the Normalized STORM Intensity (NSI) as a reporter for the proportion of H3K9me3 signal within this region relative to the entire nucleus. We found that upon 24 h of auxin treatment, H3K9me3 NSI was significantly reduced at the nuclear periphery by 25% in comparison a 0.9% increase in NSI at the nuclear interior (Fig. 7D, E). To compare the effect of heterochromatin degradation on spatial positioning and chromatin mobility, we treated HCT116 cells with GSK343, a potent EZH2 inhibitor that specifically prevents H3K27 methylation. In contrast to B-type lamin depletion, we found that upon 24 h of GSK343 treatment, H3K9me3 NSI was significantly increased at the nuclear periphery by 44% in comparison to a 0.7% decrease in NSI at the nuclear interior (Additional file 2: Fig. S6B-C). Consequently, live-cell Dual-PWS measurements confirmed a reduction in both *D* and chromatin mobility (Additional file 2: Fig. S6D). These results demonstrate that reduced B-type lamins and the removal of heterochromatic marks do not result in the same phenotype, indicative of separate contributions to proper genome organization despite their preferential interaction with each other. In line with scenario (2), these results point to a B-type lamin-dependent role in restricting chromatin mobility and maintaining the spatial positioning of heterochromatin, while histone methylation maintains a repressive chromatin environment in coordination with other cellular processes that could oppose the spreading of heterochromatic domains. Taken together, these results indicate that although mesoscale organization is maintained, lamin B1 and B2 reduction induces heterochromatin destabilization from the nuclear lamina, suggesting the detachment of LADs from the inner nuclear matrix as well.

### B-type lamin degradation shifts the preferential localization of genomic loci in live cells

Owing to the differential effect of LB1/B2 depletion on chromatin mobility and spatial distributions observed in HCT116^LMN(B1&B2)−AID^, we next tested if re-localization of peripheral chromosomes and heterochromatin was paired with gene-re-localization from the nuclear periphery. Specifically, although Hi-C demonstrated muted changes in *|s|*, compartments, and TADs, it remained possible that heterochromatin cores were moving independently of the relocation of genes in 3D space. To test this, we utilized CRISPR-Sirius, a DNA imaging system for imaging of chromosome-specific loci with a high signal-to-noise ratio in live cells [55] to measure the spatial distribution of genes in live cells upon lamin B1 and lamin B2 depletion. Since depletion of both lamins B1 and B2 resulted in nuclear wide changes in mobility and heterochromatin localization while mesoscale structures were maintained, we hypothesized that gene loci would similarly relocate in response in HCT116^LMN(B1&B2)−AID^ upon auxin treatment.

To assess the effect of B-type lamin degradation on chromosome-specific loci in live HCT116^LMN(B1&B2)−AID^ cells, we labeled genomic regions on chromosomes 3 and 19 using CRISPR-Sirius [55]. Chromosomes 3 and 19 were chosen to represent chromosomes that are localized relatively near the nuclear periphery and the nuclear interior, respectively [55–60]. Specifically, we chose one intronic gene region on Chromosome 3 (*XXYLT1*) containing ~ 333 endogenous tandem repeats, and one intronic gene region on Chromosome 19 (*TCF3*) containing ~ 36 endogenous tandem repeats as a reference to the nuclear interior. To measure the spatial distance and dynamics of the *XXYLT1* gene loci, we labeled them with CRISPR-Sirius-8XMS2 and the MS2 capsid protein MCP-HaloTag, which contains a nuclear localization sequence [55]. We visualized 2–3 pairs of loci in individual cells and quantified the average distance between the nuclear center and either the *XXYLT1* or *TCF3* gene loci, normalized to the nuclear radius (Fig. 7F–H). Our results demonstrated that the average distance between the nuclear center and *XXYLT1* significantly decreased upon lamin degradation, indicating that the targeted loci moved towards the nuclear interior. In conjunction with redistribution of heterochromatin and nuclear wide changes in chromatin mobility, the average distance between the nuclear center and *TCF3* also decreased, although to a less significant degree. These changes in subnuclear localization preferences indicate that B-type lamins constrain chromatin dynamics and gene positioning, especially at the nuclear periphery.

### Acute B-type lamin depletion alters gene expression within both LADs and Non-LADs

**Fig. 8:**
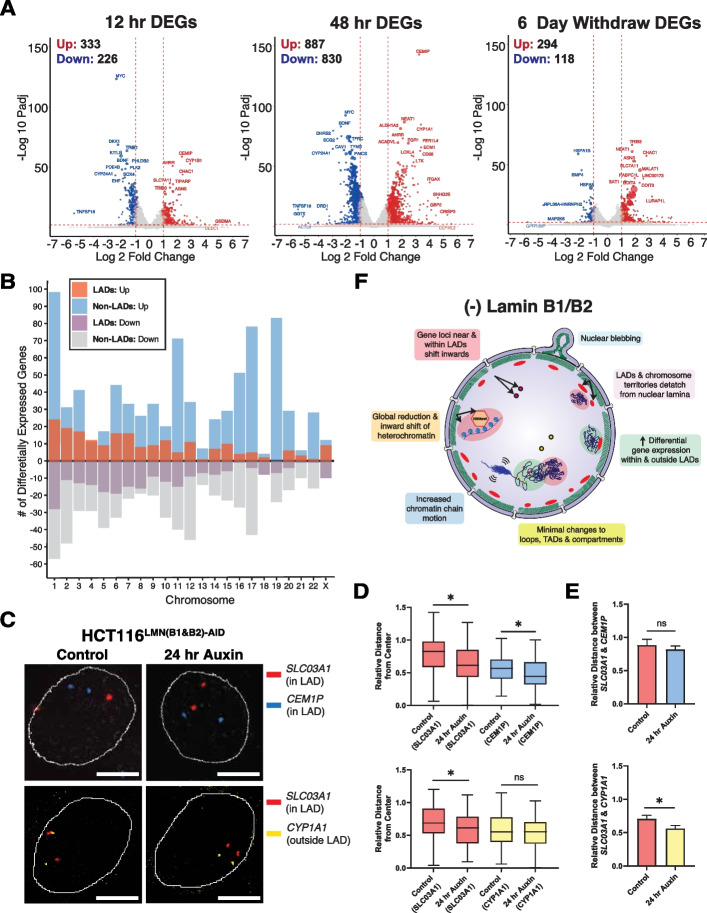
B-type lamin loss induces differential gene expression within and outside of LADs. **A** Volcano plots of DEGs after 12 h of auxin treatment, 48 h of auxin treatment, and 6 days of auxin withdrawal in HCT116^LMN(B1&B2)−AID^ cells (adjusted *P* value < 0.01 and absolute log fold change > 1). **B** Bar plot of DEGs within or outside of LADs for each chromosome across all treatment time points. **C** Representative FISH images of gene loci within or outside of LADs in HCT116^LMN(B1&B2)−AID^ cells. Scale bar = 5 μm. Data are representative of two independent biological replicates (*N* = 2). **D** Box plots of relative distances of *SLC03A1*, *CEM1P*, and *CYP1A* foci to the nuclear center (Control *SLC03A1* (top) *n* = 50; Auxin *SLC03A1* (top); *n* = 70; Control *CEM1P n* = 66; Auxin *CEM1P n* = 70; Control *SLC03A1* (bottom) *n* = 116; Auxin *SLC03A1* (bottom) *n* = 110; Control *CYP1A1 n* = 115; Auxin *CYP1A1 n* = 111)). The line within each box represents the mean; the outer edges of the box are the 25th and 75th percentiles and the whiskers extend to the minimum and maximum values. **E** Bar plots showing the relative distances between *SLC03A1* and *CEM1P* foci and *SLC03A1 and CYP1A1* foci (Mean + SEM). (*SLC03A1* & *CEM1P* (Control *n* = 39; Auxin *n* = 57), *SLC03A1* & *CYP1A1* (Control *n* = 78; Auxin *n* = 57)). For **D, E**, data was compiled from two independent biological replicates (*N* = 2). Significance was calculated by Mann–Whitney test (* < 0.05). **F** Proposed model of the effect of Lamin B1 and B2 degradation on chromatin organization. Loss of B-type lamins induces nuclear blebbing and stalled cell cycle progression, indicating their structural and functional importance as components of the nuclear periphery. Although mid-range chromatin folding (e.g., TAD # and size, A/B compartmentalization, and contact probabilities in and outside LADs) is preserved, loss of these proteins promotes increased chromatin mobility along with an inward shift of chromosome territories (e.g., Chr. 1 and 2), heterochromatin-associated domains (e.g., LADs), and gene loci (red and yellow circles), especially at the nuclear periphery. The resulting genome-wide transcriptional changes both within and outside of LADs may be a direct consequence of heterochromatin redistribution and/or gene repositioning in relation to LADs upon the loss of structural constraints at the nuclear periphery

Although the changes in mesoscale chromatin structure were muted, we hypothesized that alterations in chromatin dynamics and gene positioning could still result in profound changes in gene expression. To test this, we utilized RNA-seq to measure gene expression changes between untreated cells (0-h auxin) to 12-h auxin-treated cells, 48-h auxin-treated cells, and cells that were auxin-treated for 48 h followed by a 6-day wash off (Fig. 8A). To identify broad transcriptomic phenotypes following LMNB1/B2 degradation, we analyzed differentially expressed genes (DEGs) at each timepoint (Fig. 8A). B-type lamin depletion led to widespread differential expression: 887 genes were upregulated, and 830 genes were downregulated after 48 h of auxin treatment, when the effect of lamin depletion was most penetrant (adjusted *P* value < 0.01 and absolute log fold change > 1). Notably, the replenishment of lamins in the 6-day auxin withdraw condition resulted in 412 genes remaining altered, with the majority upregulated. To summarize, these findings indicate that B-type lamins have a distinct mechanistic role in transcriptional regulation that is independent of mechanisms responsible for maintaining higher-order chromatin folding.

Given the global changes in nuclear organization observed, we hypothesized that depletion of B-type lamins would result in differential gene expression within both LAD and non-LAD territories. We anticipated that genes within LADs would experience upregulation once freed from the heterochromatic nuclear periphery. Likewise, as mesoscale structure was preserved, we anticipated that there would be limited association between gene expression changes and TAD structure. Using the same publicly available Lamin B1 DamID data from HCT116 cells, we again segmented the genome into regions within LADs and regions outside LADs [44]. After 48 h of lamin depletion, we identified nearly 22% of enriched genes within LADs (adjusted *P* value < 0.01 and absolute log fold change > 1) (Fig. 8B; Additional file 2: Fig. S7A-B; Additional file 4). Anomalously, lamin B1 and lamin B2 depletion predominantly generated the highest number DEGs outside of LAD boundaries for all three timepoints, with the greatest number at 48 h of auxin treatment (374 genes within LADs, 1331 genes outside of LADs at 48 h) (Additional file 2: Fig. S7A, B; Additional file 4). Further, the log fold change of DEGs outside of LADs was larger in magnitude than within LADs, indicating that they were disproportionally influenced by loss of B-type lamins (Additional file 2: Fig. S7C; Additional file 4). Unexpectedly, in LAD regions, there was no clear trend towards upregulation or downregulation in gene expression. In non-LAD regions however, there appeared to be more upregulated genes (Fig. 8B; Additional file 2: Fig. S7D; Additional file 4). Based on the subtle reduction in size and increase in the number of TADs from our Hi-C experiments, we reasoned that some of the DEGs localized to LAD and non-LAD regions could potentially overlap with those within TADs. We found that at the 48-h auxin time point, the majority of DEGs both within and outside of LADs were also classified as being within TADs (Additional file 2: Fig. S7E). This overlap between LADs and inactive TAD regions, as defined by Hi-C, could help explain how chromatin structure and transcriptional changes at the nuclear periphery influence wider genome organization throughout the nucleus.

### B-type lamin degradation induces spatial reorganization within LADs to promote differential gene expression

To understand the potential mechanism behind differential gene expression upon B-type lamin degradation, we utilized fluorescence in situ hybridization (FISH) to label genomic loci that were differentially expressed. Specifically, we identified *SLC03A1* and *CEM1P*, both genes on chromosome 15 that were upregulated, within a few megabase pairs apart, and within LADs based on the public DamID data. We also performed FISH with *CYP1A1*, a gene on the same chromosome that was downregulated and located outside of a LAD based on the DamID data. First, we visualized pairs of each locus and confirmed that the two genes within LADs shifted towards the nuclear interior upon auxin treatment, consistent with *XXYLT1* in the CRISPR-Sirius experiments (Fig. 8C, D). We then measured their relative distances between each other before and after B-type lamin degradation. We found that the relative distance between *SLC03A1* and *CEM1P* within nearby LADs did not change, but the distance between *SLC03A1* and *CYP1A1* outside of a LAD decreased (Fig. 8C, E). We interpret this mobility of gene loci as LADs disassociating from the nuclear periphery and moving into the interior nuclear space as cohesive units. This cohesive internal shift of gene loci is in line with the reduction of H3K9me3 intensity at the nuclear periphery demonstrated in SMLM experiments. Based on these results, LAD redistribution may be one of the mechanisms that causes differential gene expression after lamin degradation, although genes within LADs were not entirely upregulated (Additional file 2: Fig. S8A). Our data support a model in which B-type lamin degradation does not significantly alter genome topology, but induces significantly increased chromatin mobility (e.g., re-localization of peripherally associated chromosomes, gene loci, and heterochromatin) at the nuclear scale to facilitate differential gene expression within and around LADs (Fig. 8D). With respect to the properties of DEGs within LADs and non-LADs, we performed a gene ontology analysis using Metascape and the Disease Gene Network Database. Notably, DEGs within LADs were enriched for a handful of cancers and laminopathies such as scoliosis (Additional file 2: Fig. S8B-C). Together, these data suggest that LADs maintain transcriptionally quiet states for many genes and upon depletion of B-type lamins, unevenly upregulate genes leading to altered transcriptional states that contribute to laminopathies and cancers.

To further explore how gene expression changes relate to the observed changes in chromatin dynamics, we examined the DEGs after 48 h of auxin treatment that were specific to chromosomes tested in chromosome paint and FISH experiments. Chromosome pairs 1 and 18, 2 and 19, and 15 and 22 all had more DEGs outside of LADs that were also more upregulated than downregulated (Additional file 2: Fig. S9A). We also found that the interchromosomal contact density between all pairs tested was reduced both within and outside of LADs, with specific chromosome pairs exhibiting more significant changes than others (i.e., pairs 6 and 15 and 5 and 14 both within LADs) (Additional file 2: Fig. S9B). Finally, we analyzed the transcriptional divergence of genes within and outside of LADs. Transcriptional divergence is a measure of the change in expression between minimally expressed transcripts and highly expressed transcripts. Using the Gini equation, a common metric borrowed from mainstream economics for measuring equality across a population, we calculated the Gini coefficients for each timepoint to quantify transcriptional divergence [61]. Upon 48 h of auxin treatment, the Gini coefficient for all genes decreased modestly from ~ 0.87 to 0.85. Transcriptional divergence was generally higher within LADs versus non-LADs (Additional file 2: Fig. S9C) [62]. Given that DEGs within LADs were associated with laminopathies and structural disorders while DEGs in non-LADs were primarily associated with malignancy, these results indicate distinct mechanistic consequences of gene positioning.

## Discussion

Uncovering the complex relationship between 3D genome organization and gene expression is one of the most intriguing and unresolved challenges in modern biology. Here, we applied high-resolution temporal and spatial analyses of the chromatin landscape to propose a potential mechanism underlying chromatin folding upon targeted degradation of B-type lamins. Using this approach, we identified the distinct contributions of heterochromatin- and lamina-mediated interactions that led to altered nuclear morphology and cellular functionality, heterogeneous fluid-like movements of chromatin, and stochastic repositioning of chromosomes, gene loci, and LADs at the periphery to promote differential gene expression. It should be noted, however, that the residual B-type lamins after auxin treatment and changes in cell cycling may limit the extent of the phenotypes assayed in this study.

The minimal changes seen here in genome topology reinforce that TADs may not have a massive impact on gene expression, which remains an issue of open debate due to apparent discrepancies between different approaches that are potentially explained by lack of compatibility between enhancers and promoters [63–65]. However, it is important to consider that genome topology and chromatin structure (i.e., 3D chromatin conformation) are not the same, as it is well understood that chromatin conformation is directly involved in global transcriptional regulation [39, 52, 66]. Critically, our findings indicate that the functional consequences of B-type lamin degradation do not depend on changes to the mesoscale chromatin structure (i.e., TADs, contact scaling, or compartments), but rather on alterations in chromatin dynamics and the spatial distribution of the genome. B-type lamins regulate spatial chromatin organization as we observed that their degradation leads to the movement of gene-specific loci towards the nuclear interior and increased density fluctuations in the chromatin polymer. This could possibly be explained by a transition from relatively large, anchored LADs to smaller, but still substantial domains enriched in heterochromatin. Chromatin packing scaling may increase due to a larger volume fraction of domains upon B-type lamin disruption. This may be verified by electron microscopy, which has previously been used to indicate that the mobility of peripheral LADs is largely confined to the layer of densely staining heterochromatin [67]. Conversely, it has been proposed that LADs may not play a direct gene silencing, but rather that the association of genomic loci with the nuclear lamina may reinforce transcriptional states [19]. This could help explain why DEGs within LADs were not primarily upregulated when LADs shift away from the nuclear lamina, but those outside of LAD boundaries were. However, recently proposed heteropolymer models such as the Minimal Chromatin Model (MiChroM) [68] and ChIP-seq assays across several human cell types [69] are in line with our experimental data and therefore support the explanation of chromatin dynamics in establishing higher-order chromatin organization and differential gene expression. Chromatin dynamics may change because in the presence of B-type lamins, LADs are attached to the nuclear periphery and most of the captured motion by live-cell dual-PWS is due to the non-LAD polymeric structures, which are small and therefore have a larger diffusion coefficient. When B-type lamins are degraded, however, the new non-attached LADs begin to shift away which leads to a substantial increase in the fractional moving mass. As demonstrated by measuring TAD sizes that were associated with LADs from the DamID data, these domains are larger and physically move more slowly throughout the nuclear area which leads to a lower diffusion coefficient. Therefore, our dual-PWS measurements are consistent with those obtained by SMLM imaging, which demonstrated the physical breakdown of H3K9me3 components at the nuclear periphery.

In recent Hi-C studies comparing conventional and inverted nuclei that form from LBR depletion or Lamin A/C depletion, relatively few changes in compartment and chromatin connectivity are observed [17]. In line with our experimental data, these studies also found that while loss of attraction between heterochromatin and the nuclear lamina maintained genome compartmentalization, it resulted in spatial repositioning of individual A and B compartments. Similar to phenotypes resulting from cellular senescence, in which loss of lamin B1 is a biomarker, B-type lamin reduction led to increased nuclear volume, slower cell cycle progression, and nuclear blebbing [14, 70, 71]. Like B-type lamin degradation, cellular senescence also involves cell-type-dependent transcriptional reprogramming that coincides with an inward shift of heterochromatin and LADs from the nuclear periphery [72]. Although recent Hi-C and high-resolution capture Hi-C (cHi-C) studies in IMR90 human diploid fibroblasts reported no differences in the distribution of A/B compartments as shown in this work, it was also reported that oncogenic RAS-induced senescence extensively reduced cohesin binding and re-organized loops [73]. The overall maintenance of loops, TADs, and compartments upon B-type lamin degradation indicates that genome topology is robust to perturbation of chromatin elasticity [42]. These modest effects on higher-order chromatin structure could be due to multiple mechanisms, including (1) redundancy of LMNA proteins after reduced expression of LMNB1 and LMNB2, (2) a distinct role for LBR in chromatin organization, or (3) the cellular memory of chromatin states as an epigenetic mechanism of transcriptional control due to association with B-type lamin domains that is not lost on their disruption.

Loss of A-type lamins have been reported to increase chromatin dynamics in the nuclear interior by inducing a dramatic transition from slow anomalous diffusion to fast and normal diffusion [74], and when co-depleted with LBR, leads to a reduction of heterochromatin tethered to the nuclear periphery, similar to the observed inverted nuclear architecture of rods [17, 18]. Similar to B-type lamins, inner nuclear membrane proteins such as LBR, emerin, and various isoforms of lamina-associated polypeptides 1 (LAP1) and LAP2 have also been reported to bind lamins and tether repressive chromatin at the nuclear periphery [12, 18, 75], and their dysregulation has been linked to severe genetic disorders [13, 14, 76]. Aberrant localization or expression of A-type lamins, B-type lamins, and inner nuclear membrane proteins have also been related to cancer development and metastasis, largely associated with disrupted nuclear morphology and DNA damage [14, 62, 77]. It is possible that these functional consequences arise from nuclear-scale shifts in chromatin dynamics and gene repositing within LADs. The joint degradation of B-type lamins in chromatin resulted in differential gene expression patterns both within and outside of LADs. As such, our work provides further evidence that LADs function not solely through the effect on transcriptional repression but are involved in the maintenance of transcriptionally active genomic sites.

Our findings demonstrate the importance of exploring relationships between imaging and gene expression data on a nuclear scale. It also highlights a need for the development of advanced methods to investigate chromatin dynamics as well as an evolution in chromatin modeling to better understand how the temporal evolution of chromatin can alter gene transcription without alterations in mesoscale structure. While Hi-C methods enable the reconstruction of 3D chromosome and genome structures, one of the key limitations of this technique is that that long-range frequencies can be noisy and unreliable, often depending on experimental factors such as the length of restriction fragments, GC content, and read depth [78]. Further, this is a fixed-cell technique and thus does not allow for millisecond resolution of chromatin structure and dynamics. Consequently, Hi-C and derivative techniques are incapable of detecting the complexities of dynamic chromatin reorganization alone. Thus, incorporating multi-modal imaging techniques in addition to Hi-C can provide a better method for characterizing nanoscale structural alterations in chromatin packing in real time. Future work pairing super-resolution microscopy in combination with PWS microscopy and high-resolution electron microscopy, involving labeling for markers specific to heterochromatin, could help elucidate the nanoscopic changes in chromatin structure during lamin B disruption [30, 39]. Such an approach can also aid in providing further understanding of the mechanisms that maintain the phenotypes involving degraded lamins or inverted nuclei.

## Conclusion

Our investigation focuses on understanding the mechanisms by which the rapid inhibition of both B-type lamins simultaneously would alter genome organization, gene expression, and cell function. Our results in mammalian cells indicate a direct link between transcription changes mediated by B-type lamins near LADs that is not due to higher-order chromatin structure transformation. Transcriptionally, expression in both LAD and non-LAD territories are transformed, with distinct disease associations from the expression patterns depending on the genomic territory (LADs associated with structural deformities and non-LADs with malignancies). Further, our results demonstrate that upon B-type lamin degradation, chromatin conformation and chromatin connectivity show subtle changes, although chromatin territories expand. However, the greatest alterations in chromatin conformation and connectivity occur closest to the nuclear periphery in comparison to the nuclear interior. Consequently, these findings demonstrate a distinct mechanistic role of B-type lamins in chromatin organization and cellular function. Our results therefore warrant further investigation of the dynamic relationship between the nuclear lamina and chromatin organization.

## Materials and methods

### HEK293T cell culture

HEK293T cells (ATCC, #CRL-1573) were grown in Dulbecco’s Modified Eagle’s Medium (DMEM) supplemented with 10% FBS (#16000–044, Thermo Fisher Scientific, Waltham, MA) and penicillin–streptomycin (100 μg/ml; #15140–122, Thermo Fisher Scientific, Waltham, MA). All cells were cultured under recommended conditions at 37°C and 5% CO_2_. All cells in this study were maintained between passage 5 and 20. Cells were allowed at least 24 h to re-adhere and recover from trypsin-induced detachment. All cells were tested for mycoplasma contamination (ATCC, #30-1012K) before starting experiments, and they have given negative results.

### HCT116 cell culture

HCT116 cells (ATCC, #CCL-247) were grown in McCoy’s 5A Modified Medium (#16600–082, Thermo Fisher Scientific, Waltham, MA) supplemented with 10% FBS (#16000–044, Thermo Fisher Scientific, Waltham, MA) and penicillin–streptomycin (100 μg/ml; #15140–122, Thermo Fisher Scientific, Waltham, MA). All cells were cultured under recommended conditions at 37°C and 5% CO_2_. All cells in this study were maintained between passage 5 and 20. Cells were allowed at least 24 h to re-adhere and recover from trypsin-induced detachment. All imaging was performed when the surface confluence of the dish was between 40–70%. All cells were tested for mycoplasma contamination (ATCC, #30-1012K) before starting perturbation experiments, and they have given negative results.

## Method details

### Transfection and colony isolation for creating AID cell lines

Stable transfection was achieved as previously described [33]. Briefly, we generated conditional human HCT116 mutants by homology-directed repair (HDR)-mediated gene tagging using CRISPR-Cas9. Around 60–70% confluent cells expressing OsTIR1 were plated at 3 × 10^5^ cells in a 6-well plate and cultured for 24 h at 37°C. On the next day of transfection, 4 mL of 200 ng/mL CRISPR plasmid, 3 mL of 200 ng/mL donor plasmid, 90 mL of OptiMEM I Reduced Serum Medium (Gibco, #31985070), and 8 mL of FuGENE 6 Transfection Reagent (Promega, #E2691) were mixed and incubated at room temperature for 15 min before being applied to the cells. For antibiotic selection and colony formation, Hygromycin B Gold (Gibco, #10,687010), 100 mg/mL was used. For colony isolation, single colonies were picked under a stereo microscope and transferred to a 96-well plate containing 10 mL of trypsin/ EDTA and neutralized with 200 mL of media. After 2 weeks of culture, the cells were transferred to a 24-well plate. After a few days of culture, genomic DNA was isolated. Briefly, the cells were harvested, lysed with SDS buffer (100 mM NaCl, 50 mM Tris–Cl pH 8.1, 5mM EDTA, and 1% wt/vol SDS), and treated with proteinase k (New England Biolabs, #P8102) (0.6 mg/mL) at 55 °C for 2 h. Then, the lysis solutions were treated with PCl (phenol/ chloroform/ isoamyl alcohol) and used in EtOH precipitation. Genomic DNA pellets were washed with 70% EtOH and resuspended in RNase-containing water. The PCR reaction was set up using 0.5 U of Taq DNA Polymerase (G Biosciences, #786–447) (1 × PCR Buffer), 0.5 mM primers, and 1 mL of genomic DNA from the HCT116 CMV-OsTIR1 parental cells to a 20-mL total volume reaction mixture. PCR was performed using the following conditions: 30 cycles of 98 °C for 2 min, 55 °C for 30 s, and 68 °C for 0.5 min/kb. PCR products were examined for biallelic insertion using agarose gel electrophoresis. Initially, progenitor cells were produced by integrating OsTIR1 (CMV-OsTIR1) into the safe-harbor AAVS1 locus. We then co-transfected the donor template plasmid with Cas9 and sgRNAs that targeted the STOP codon of the LMNB1 or LMNB2 genes in HCT116 cells expressing OsTIR1. To generate cells with mAID in both genes, we simultaneously transfected the two donor templates with two sgRNA that target both genes. Cells expressing mClover for each protein target (LMNB1, LMNB2, or LMNB1 & B2) were sorted and grown as single-cell colonies. We PCR-screened 179 colonies in total to identify clones with homozygous LAMINB1-mAID (5 + /40 colonies) and LAMIN B2-mAID (5 + /55 colonies) as well as clones that had homozygous mAID in both genes (3 + /84 colonies).

### Primers and sgRNA

Appropriate primers were designed to check the insertion by PCR. For creating stable cell lines, primer sets detected both the wild type (WT) (1–1.5 kb) and inserted alleles (1–1.5 kb plus the size of the insertion), and another primer set to detect only the inserted allele. The first primer set was designed outside of the homology arms. Primers and sgRNA sequences used to create all AID cell lines are listed in Additional file 1.

### Plasmids for AID cell lines

The donor construct contained the AID domain fused to mClover and an intervening T2A site with a hygromycin resistance marker. The construct was flanked by 50-base pair homology arms corresponding to the last exon region of the sgRNA recognition sequence and Cas9 cleavage site. To identify a CRISPR–Cas9 targeting site, we chose an appropriate sequence within 50-bp upstream or downstream from the stop codon. The following target finder sites were used to construct the CRISPR–Cas9 plasmid: IDT custom Alt-R guide design and WEG CRISPR finder. Construction of the CRISPR–Cas9 plasmid and donor plasmids have been previously described [33]. The AAVS1 T2 CRISPR in pX330 plasmid (Addgene plasmid # 72833; http://n2t.net/addgene:72833; RRID: Addgene_72833) is based on pX330-U6-Chimeric_BB-CBh-hSpCas9 from Dr. Feng Zhang (Addgene #42230) (Cong et al., Science, 2013) and was a gift from Masato Kanemaki. The AAVS1 target sequence is described in Mali et al. (Mali et al., Science, 2013). pMK232 (CMV-OsTIR1-PURO) was a gift from Masato Kanemaki (Addgene plasmid # 72834; http://n2t.net/addgene:72834; RRID: Addgene 72834). pMK364 (CMV-OsTIR1-loxP-PURO-loxP) was a gift from Masato Kanemaki (Addgene plasmid # 121184; http://n2t.net/addgene:121184; RRID: Addgene_121184). pMK290 (mAID-mClover-Hygro) was a gift from Masato Kanemaki (Addgene plasmid # 72828; http://n2t.net/addgene:72828; RRID: Addgene_72828).

### Auxin treatment

For auxin treatment, HCT116^LMN(B1&B2)−AID^ cells were plated at 50,000 cells per well of a 6-well plate (Cellvis, P12-1.5H-N). Cells were given at least 24 h to re-adhere prior to treatment. To induce expression of OsTIR1, 2 μg/ml of doxycycline (Fisher Scientific, #10592–13-9) was added to cells 24 h prior to auxin treatment. For live-cell flow cytometry, western blots, RT-qPCR, RNA-seq, and in situ Hi-C experiments, 1000 μM 3-Indoleacetic acid (IAA, Sigma-Aldrich, #12886) was solubilized in 100% EtOH before each treatment as a fresh solution. For live-cell confocal microscopy, fixed-cell flow cytometry, fixed-cell immunofluorescence, CRISPR-Sirius fluorescent imaging, and Dual-PWS experiments, 1000 μM Indole-3-acetic acid sodium salt (IAA, Sigma-Aldrich, #6505–45-9) was solubilized in RNase-free water (Fisher Scientific, #10–977-015) before each treatment as a fresh solution and added to HCT116^LMN(B1&B2)−AID^ cells. Optimal auxin treatment time was determined based on the results from western blot, immunofluorescence, and flow cytometry experiments (Fig. 1D).

### GSK343 treatment

For GSK343 treatment, HCT116 cells were plated at 50,000 cells per well of a 6-well plate (Cellvis, P12-1.5H-N). Cells were given at least 24 h to re-adhere before treatment. GSK343 (Millipore Sigma, #SML0766) was dissolved in DMSO to create a 10 mM stock solution. This was further diluted in complete cell media to a final treatment concentration of 10 µm.

### Fixed-cell flow cytometry (FACS) analysis

Flow cytometry analysis for HCT116^LMNB1−AID^, HCT116^LMNB2−AID^, and HCT116^LMN(B1&B2)−AID^ cells for AID system verification and nuclear morphology experiments was performed on the Amnis ImageStreamXTM, located at the University of Virginia Flow Cytometry Core Facility in Charlottesville, VA. To assess the degree of apoptosis induced by auxin treatment, we used the Annexin V APC Kit (Cayman Chemical, #601410) and followed the manufacturer’s protocol. Flow cytometry analysis for HCT116^LMN(B1&B2)−AID^ cells to determine proper auxin treatment concentration was performed on a BD LSRFortessa Cell Analyzer FACSymphony S6 SORP system, located at the Robert H. Lurie Comprehensive Cancer Center Flow Cytometry Core Facility at Northwestern University in Evanston, IL. For all FACS analysis, the same protocol was used. After 24 h of doxycycline treatment followed by auxin treatment, cells were harvested and fixed. Briefly, cells were washed with DPBS (Gibco, #14190–144), trypsinized (Gibco, #25200–056), neutralized with media, and then centrifuged at 500 × *g* for 5 min. Cells were then resuspended in 500 μL of 4% PFA and DPBS and fixed for 10 min at room temperature, followed by centrifugation and resuspension in cold FACS buffer (DPBS with 1% of BSA and 2mM EDTA added) at 4°C until analysis could be performed the following day. For propidium iodine (PI) staining, cells were harvested, and cell pellets were washed with DPBS. Then, DPBS was removed and ice-cold 70% EtOH was added drop by drop to the cell pellets for fixation. Cells were fixed for 3 h at 4°C. After fixation, EtOH was removed, and cells were washed twice with DPBS. PI staining solution (20 µL/ sample; BioLegend, #421301) including RNase A (0.5 µL/ sample; Thermo Fisher Scientific, #EN0531) was added to the cells and incubated with the cells for 30 min protected from light. Staining solution was subsequently removed, and cells were washed twice using 2% FBS containing DPBS. Finally, cells were filtered through 70 µM filters. All flow cytometry data were analyzed using FlowJo software.

### Live-cell imaging for growth kinetics

The Incucyte Live cell imaging system (Sartorius) was used to measure growth kinetics of the cells. Cells were seeded to 96-well plates and next day, as soon as the cells were treated with doxycycline, the system was set to collect images every 2 h in phase and green channels (phase: proliferation of the cells, green: degradation of lamins). Twenty-four hours later, cells were treated with IAA and the system collected images for 72 h.

### Protein detection and antibodies

HCT116^LMNB1−AID^, HCT116^LMNB2−AID^, and HCT116^LMN(B1&B2)−AID^ cells were lysed using Radio Immuno Precipitation Assay (RIPA) buffer (Sigma-Aldrich, #R0278) with protease inhibitor added (Sigma-Aldrich, #P8340). Cell lysates were quantified with a standard Bradford assay using the Protein Assay Dye Concentrate (BioRad, #500–0006) and BSA as a control. Heat denatured protein samples were resolved on a 4–12% bis–tris gradient gel, transferred to a PVDF membrane using the Life Technologies Invitrogen iBlot Dry Transfer System (Thermo Fisher Scientific, IB1001) (20 V for 7 min), and blocked in 5% nonfat dried milk (BioRad, #120–6404) in 1 × TBST. Whole-cell lysates were blotted against the following primary antibodies: Lamin B1 (Cell Signaling, #13435, dilution 1:1000), Lamin B2 (Cell Signaling, #12255, dilution 1:1000), and alpha-tubulin (Thermo Fischer Scientific, #62204, dilution 1:2000). The following secondary antibodies were used: anti-rabbit IgG HRP (Promega, #W4018). Blots were incubated with the primary antibody overnight at 4 °C, followed by incubation with the secondary antibodies for 1 h at room temperature. To develop blots for protein detection, chemiluminescent substrates were used (Thermo Fischer Scientific, #32106). To quantify the western blot bands, we used the iBright Analysis Software from Thermo Fisher Scientific to define bands as regions of interest. By measuring the mean grey intensity values, the final relative quantification values were calculated as the ratio of each protein band relative to the lane’s loading control for all three replicates. Uncropped blots are included in Additional file 3.

### Quantitative real-time PCR and RNA isolation

Total RNA from transfected cells was harvested using the RNeasy Plus Mini Kit (Qiagen, #74134) following the manufacturer’s protocol. One milligram of RNA was converted to cDNA using the Applied Biosystems High-Capacity RNA-to-cDNA Kit (Thermo Fisher Scientific, #4387406). The GAPDH or HPRT-1 gene was used as an internal control for analysis. RT-qPCR was performed on a StepOnePlus Applied Biosystems instrument with SYBR Green. RNA quantity was measured using the Nanodrop 2000 Spectrophotometer at 260nm.

### Fixed-cell immunofluorescence

HCT116^LMN(B1)−AID^ cells, HCT116^LMN(B2)−AID^ cells, or HCT116^LMN(B1&B2)−AID^ cells at a low passage (< P10) were plated at 100,000 cells per well of a 6-well glass-bottom plate (Cellvis, #P06-1.5H-N). Following auxin treatment, cells were washed twice with 1 × Phosphate Buffered Saline (PBS) (Gibco, #10010031). Cells were fixed with 4% paraformaldehyde (PFA) (Electron Microscopy Sciences, #15,710) for 10 min at room temperature, followed by washing with PBS 3 times for 5 min each. Cells were permeabilized using 0.2% TritonX-100 (10%) (Sigma-Aldrich, #93443) in 1 × PBS, followed by another wash with 1 × PBS for 3 times for 5 min each. Cells were blocked using 3% BSA (Sigma-Aldrich, #A7906) in PBST (Tween-20 in 1 × PBS) (Sigma-Aldrich, #P9416) at room temperature. The following primary antibodies were added overnight at 4 °C: anti-lamin B1 (Abcam, #ab16048, dilution 1:1000), anti-lamin B2 (Abcam, #ab155319, dilution 1:1000), anti-lamin A (Abcam, #ab8980, dilution 1:1000), anti-H3K27ac (Abcam, #ab177178, dilution 1:7000), and anti-H3K27me3 (Abcam, #ab6002, dilution 1:200). Cells were washed with 1 × PBS 3 times for 5 min each. Cells were washed with 1 × PBS 3 times for 5 min each. The following secondary antibodies were added for 1 h at room temperature: Goat anti-Rabbit IgG (H + L) Alexa Fluor 568 (Abcam, #ab175471, dilution 1:1000), Goat anti-Mouse IgG (H + L) Highly Cross-Adsorbed Secondary Antibody, Alexa Fluor Plus 647 (Thermo Fisher Scientific, #A32728, dilution 1:200), and Invitrogen Goat anti-Rabbit IgG (H + L) Highly Cross-Adsorbed Secondary Antibody, Alexa Fluor 647 (Thermo Fisher Scientific, #A-21245). Cells were washed with 1 × PBS 3 times for 5 min each. Finally, cells were stained with DAPI (Thermo Fisher Scientific, #62248, diluted to 0.5 μg/mL in 1 × PBS) for 10 min at room temperature. Prior to imaging, cells were washed with 1 × PBS twice for 5 min each.

### Preparation of Hi-C Libraries for in situ Hi-C

In situ Hi-C was performed as previously described [2]. Briefly, 2–5 million cells at 80% confluence were detached and pelleted by centrifugation at 300 × G for 5 min. Cells were resuspended in fresh medium at a concentration of 1E6 cells per 1 mL media. In a fume hood, 1% formaldehyde was used to crosslink cells, with 10 min of incubation at room temperature with mixing. 2.5M glycine solution was added to a final concentration of 0.2M to quench the reaction, and the reaction was incubated at room temperature for 5 min with gentle rocking. Samples were centrifuged at 300 × *g* for 5 min at 4°C. Cells were resuspended in 1mL of ice-cold PBS and spun at 300 × *g* for 5 min at 4°C. Cell pellets were flash-frozen in liquid nitrogen and either stored at − 80°C or used immediately for lysis and restriction digest. Nuclei were permeabilized. Two hundred fifty microliters of Hi-C lysis buffer (10mM Tris–HCl pH 8.0, 10mM NaCl, 0.2% Igepal CA930 (Sigma-Aldrich, #I3021)) with 50 μL of protease inhibitors (Sigma-Aldrich, #P8340) was added to each crosslinked pellet of cells. After incubation on ice for > 15 min, samples were centrifuged at 2500 × *g* for 5 min and washed with 500 μL of ice-cold Hi-C lysis buffer. Pelleted nuclei were resuspended in 50 μL of 0.5% sodium dodecyl sulfate (SDS) (Sigma-Aldrich, #436143) and incubated at 62°C for 10 min. Next, 145 μL of water (Fischer Scientific, #10–977-015) and 25 μL of 10% Triton X-100 (Sigma-Aldrich, #93443) were added to quench the SDS. Samples were mixed and incubated at 37°C for 15 min. DNA was digested with 100U of MboI restriction enzyme (New England Biolabs, #R0147) and 25 μL of 10X NEBuffer 2 (New England Biolabs, #B7002S). Chromatin was digested overnight at 37°C with rotation. Samples were incubated at 62°C for 20 min to inactivate MboI, and then cooled to room temperature. The ends of restriction fragments were labeled using biotinylated nucleotides (Thermo Fisher Scientific, #19524016) and ligated in a small volume (~ 900 μL) using 10X NEB T4 DNA ligase buffer (New England Biolabs, #B0202) and DNA Polymerase I, Large (Klenow) Fragment (New England Biolabs, #M0202) after 1 h of incubation at 37°C. Samples were mixed and incubated at room temperature for 4 h prior to reversal of crosslinks. We added 50 μL of 20 mg/mL proteinase K (New England Biolabs, #P8102) and 120 μL of 10% SDS and incubated samples at 55°C for 30 min. Next, 130 μL of 5M sodium chloride was added and samples were incubated at 68°C overnight. Ligated DNA was purified and sheared to a length of ~ 400 bp, as previously described [2] using a LE220-plus Focused-ultrasonicator (Covaris, #500569) and AMPure XP beads (Beckman Coulter, #A63881). DNA was quantified using the Qubit dsDNA High Sensitivity Assay Kit (Thermo Fisher Scientific, #Q33230) and undiluted DNA was run on a 2% agarose gel to verify successful size selection. Point ligation junctions were pulled down with 10 m/mL Dynabeads MyOne Steptavidin T1 beads (Thermo Fisher Scientific, #65601) and prepared for Illumina sequencing using Illumina primers and protocol (Illumina, 2007) as previously described [2]. Paired-end sequencing was performed using the Illumina HiSeq 2000 OR 2500 platform. A no-ligation control was also used.

### Hi-C data processing and analysis

Juicebox was used to visualize Hi-C contact maps [79]. All Hi-C data reported were produced using Illumina paired-end sequencing. We followed the Hi-C data processing pipeline that has previously been described [2]. This pipeline uses the Burrows-Wheeler single end aligner (BWA) [80] to map each read end separately to the hg19 reference genome, removes reads that map to the same fragment, removes duplicate or near-duplicate reads, and filters the remaining reads based on the mapping quality score. All analysis (i.e., aggregate peak analysis) and annotations (i.e., annotation of domains, assigning loci to subcompartments, and peaks) were performed as previously described [2, 81]. All contact matrices were KR-normalized with Juicer. Domains were annotated using TopDom.

### Chromosome paint hybridization

Cells were trypsinized, resuspended in appropriate growth media and then plated onto glass coverslips and allowed to adhere for approximately 16 h prior to fixation. Cells were rinsed once in PBS prior to fixation in 4% PFA for 10 min at room temperature. Cells were then washed 3 times in PBS, followed by incubation in PBS with 0.01% Triton X-100 at room temperature 3 times for 3 min, then incubation in 0.5% Triton X-100/1 × PBS at room temperature for 15 min. Cells were then incubated in 20% glycerol/ PBS at room temperature for 3 h. Cells were washed in PBS 3 times for 10 min each and then incubated in 0.1 N HCl for 5 min at room temperature. The cells were incubated in 2 × SSC twice for 3 min each before being placed in 50% formamide (Electron Microscopy Sciences)/2 × SSC at room temperature for approximately 18 h. After the addition of chromosome paints (Metasystems) to the coverslips, slides were heated to 75°C for 2 min before being placed at 37° for approximately 72 h for hybridization. After hybridization, coverslips were washed in 2 × SSC washes at 37° three times for 5 min each, followed by washes in 0.1 × SSC at 60° three times for 5 min each. The coverslips were then washed in 4 × SSC/0.2% Tween-20 three times for 3 min and mounted on microscope slides using Diamond antifade with DAPI (Invitrogen). The following chromosome paints were used in this study: Chr 1: D-0301–050-OR, Chr 2: D-0302–050-OR, Chr 18: D-0318–050-OR, and Chr 19: D-0319–050-OR.

### Dual-PWS imaging

Briefly, PWS measures the spectral interference signal resulting from internal light scattering originating from nuclear chromatin. This is related to variations in the refractive index distribution (Σ) (extracted by calculating the standard deviation of the spectral interference at each pixel), characterized by the chromatin packing scaling (*D*). *D* was calculated using maps of Σ, as previously described [39, 49, 51, 52]. Measurements were normalized by the reflectance of the glass medium interface (i.e., to an independent reference measurement acquired in a region lacking cells on the dish). This allows us to obtain the interference signal directly related to refractive index (RI) fluctuations within the cell. Although it is a diffraction-limited imaging modality, PWS can measure chromatin density variations because the RI is proportional to the local density of macromolecules (e.g., DNA, RNA, proteins). Therefore, the standard deviation of the RI (Σ) is proportional to nanoscale density variations and can be used to characterize packing scaling behavior of chromatin domains with length scale sensitivity around 20–200 nm, depending on sample thickness and height. Changes in *D* resulting from each condition are quantified by averaging over nearly 2000 cells, taken across 3 technical replicates. Live-cell PWS measurements obtained using a commercial inverted microscope (Leica, DMIRB) using a Hamamatsu Image-EM charge-coupled device (CCD) camera (C9100-13) coupled to a liquid crystal tunable filter (LCTF, CRi VariSpec) to acquire monochromatic, spectrally resolved images ranging from 500 to 700 nm at 2-nm intervals as previously described [46, 49, 50]. Broadband illumination is provided by a broad-spectrum white light LED source (Xcite-120 LED, Excelitas). The system is equipped with a long pass filter (Semrock BLP01-405R-25) and a × 63 oil immersion objective (Leica HCX PL APO). Cells were imaged under physiological conditions (37°C and 5% CO_2_) using a stage top incubator (In vivo Scientific; Stage Top Systems). All cells were given at least 24 h to re-adhere before treatment (for treated cells) and imaging.

### Dynamic PWS measurements

Dynamic PWS measurements were obtained as previously described [49]. Briefly, dynamics measurements (${\Sigma }_{t}^{2}$, fractional moving mass (${m}_{{\text{f}}} )$, and diffusion) are collected by acquiring multiple backscattered wide-field images at a single wavelength (550 nm) over time (acquisition time), to produce a three-dimensional image cube, where ${\Sigma }_{t}^{2}$ is temporal interference and *t* is time. Diffusion is extracted by calculating the decay rate of the autocorrelation of the temporal interference as previously described [49]. The fractional moving mass is calculated by normalizing the variance of ${\Sigma }_{t}^{2}$ at each pixel. Using the equations and parameters supplied and explained in detail in the supplementary information of our recent publication [49], the fractional moving mass is obtained by using the following equation to normalize ${\Sigma }_{t}^{2}$ by${\rho }_{0}$, the density of a typical macromolecular cluster:

$${\Sigma }_{t}^{2}\left(\frac{{\uppi \rho }_{0}}{2{\Gamma }^{2}{k}^{3}{n}_{i}}\right){\left(\frac{{NA}_{i}}{{NA}_{c}}\right)}^{2}{\left(\frac{{n}_{1}}{{n}_{m} - {n}_{1}}\right)}^{2}= {\rho }_{0}{{\text{V}}}_{cm}\varphi = {m}_{c}\varphi = {m}_{{\text{f}}}$$

With this normalization, $${\Sigma }_{t}^{2}$$ is equivalent to $${m}_{{\text{f}}}$$, which measures the mass moving within the sample. This value is calculated from the product of the mass of the typical moving cluster ($${m}_{c})$$ and the volume fraction of mobile mass ($$\varphi$$). $${m}_{c}$$ is obtained by $${m}_{c}= {{\text{V}}}_{cm}{\rho }_{0}$$, where $${{\text{V}}}_{cm}$$ is the volume of the typical moving macromolecular cluster. To calculate this normalization, we approximate $${n}_{m}$$ = 1.43 as the refractive index (RI) of a nucleosome, $${n}_{1}$$ = 1.37 as the RI of a nucleus, $${n}_{i}$$ = 1.518 as the refractive index of the immersion oil, and $${\rho }_{0}$$ = 0.55 g cm^−3^ as the dry density of a nucleosome. Additionally, $$k$$ = 1.57E5 cm^−1^ is the scalar wavenumber of the illumination light, and $$\Gamma$$ is a Fresnel intensity coefficient for normal incidence. $${NA}_{c}$$ = 1.49 is the numerical aperture (NA) of collection and $${NA}_{i}$$ = 0.52 is the NA of illumination. As stated previously [49], $${\Sigma }_{t}^{2}$$ is sensitive to instrument parameters such as the depth of field and substrate refractive index. These dependencies are removed through normalization with the proper pre-factor calculated above for obtaining biological measurements. It should also be noted that backscattered intensity is prone to errors along the transverse direction [49]. Due to these variations, these parameters are more accurate when calculating the expected value over each pixel.

### Regional PWS analysis

We used PWS to calculate *D* values via measuring the variations in spectral light interference resulting from light scattering due to heterogeneities in chromatin density as previously described [51]. The same cells used to analyze average chromatin packing scaling in whole nuclear regions were used for regional chromatin packing scaling analysis. For the periphery characterization, individual nuclei were segmented into 6 ribbons of 260 nm width each using MATLAB. The remaining region at the center of the nucleus was classified as the center. We calculated the average *D* for each pixel, followed by averaging all these values to estimate the average *D* in each region.

### Confocal imaging

For CRISPR-Sirius, when MCP-Halo was added to cells for fluorescent imaging, HaloTag-JF646 was added to the cells at 10 μM 24 h before imaging and incubated overnight at 37°C and 5% CO_2_. On the day of imaging, live cells were washed three times with DPBS (Gibco, #14190–144) and further incubated with phenol-red free media (Cytiva, #SH30270.01). The optical instrument was built on a commercial inverted microscope (Eclipse Ti-U with the perfect focus system, Nikon). Images of live cells were collected using a × 100 objective and sent to an electron-multiplying CCD (iXon Ultra 888, Andor). A 637-nm laser (Obis, Coherent) was co-illuminated through a × 100/ 1.49 NA (numerical aperture) oil objective lens (SR APO TIRF, Nikon) with an average power at the sample of 3 to 10 kW/cm^3^. The microscope stage incubation chamber was maintained at 37 °C and supplemented with 5% CO_2_. For single image acquisition, at least 50 frames were taken at 30 ms exposure time and a gain of 150. Z-stack images were acquired at 0.024 µm per step for a total of 401–701 frames depending on the size of the nuclei being imaged [46, 49, 50]. Images of fixed-cells previously transfected with CRISPR-Sirius plasmids were imaged using the Nikon SoRa Spinning Disk confocal microscope equipped with a Hamamatsu ORCA-Fusion Digital CMOS camera. Images were collected using a × 60/1.42 NA oil immersion objective mounted with a × 2.8 magnifier. mClover was excited with a 488-nm laser, HaloTag-JF646 was excited with a 640-nm laser, and DAPI was excited with a 405-nm laser. Imaging data were acquired by Nikon acquisition software.

### SMLM sample preparation and imaging

The primary antibody rabbit anti-H3K9me3 (Abcam, #ab176916) was aliquoted and stored at − 80 °C. The secondary antibody goat anti-rabbit AF647 (Thermo Fisher Scientific, #A-21245) was stored at 4 °C. The cells were plated on No. 1 borosilicate bottom eight-well Lab-Tek Chambered cover glass with at a seeding density of 1.25 × 10^4^. After 48 h, the cells were fixed in 3% paraformaldehyde in PBS for 10 min, and then subsequently washed with PBS once for five min. Thereafter, the samples were quenched with freshly prepared 0.1% sodium borohydride in PBS for 7 min and rinsed with PBS three times at room temperature. The fixed samples were permeabilized with a blocking buffer (3% bovine serum albumin (BSA), 0.5% Triton X-100 in PBS) for 20 min and then incubated with rabbit anti-H3K9me3 in blocking buffer for 1–2 h at room temperature and rinsed with a washing buffer (0.2% BSA, 0.1% Triton X-100 in PBS) three times. The fixed samples were further incubated with the corresponding goat secondary antibody–dye conjugates, anti-rabbit AF647, for 40 min, washed thoroughly with PBS three times at room temperature and stored at 4 °C.

### SMLM image analysis

To segment nuclei, nuclear, nuclear periphery, and nuclear interior masks were generated using built-in OpenCV Python package methods to perform combination erosion and dilation operations on the reconstructed H3K9me3 image. To identify the nuclear periphery and what was considered the nuclear lamina, the *cv2.findContours* method was used to first identify the contour around each nucleus. Contours were then dilated *n* = 5 times with a 5 × 5 one’s kernel to approximate 100 nm distance from the identified edge. Contours then underwent a bitwise and multiplication with the nuclear mask to keep only the dilated portion of the contour that lies within the original nuclear mask. Nuclear interior masks were identified as the subtraction of the larger nuclear mask by the dilated contour. To measure Normalized STORM Intensity (NSI), reconstructed images were generated using the built-in ThunderSTORM Fiji plugin average shifted histograms algorithms where pixel intensity in resultant image is a proxy for count of localized events within that bin (bin size = 26 nm per pixel). NSI is the quotient of the sum of all pixel intensities within a specific regional mask (e.g., nuclear periphery) normalized by the area of that mask (number of pixels) and the total nuclear intensity normalized by nuclear area. NSI reports the proportion of signal intensity, which is a proxy for the number of events within a given area, relative to the entire nuclear signal. In the equation below, *A*_mask_ is the nuclear regional mask area, *s*_*i*_ is the *i*th pixel of that regional mask, *A*_nuc_ is area of total nuclear mask, and *s*_*j*_ is *j*th pixel of that total nuclear mask.

$$NSI= \frac{\frac{1}{{A}_{mask}}\sum_{i=1}^{N}{s}_{i}}{\frac{1}{{A}_{nuc}}\sum_{j=1}^{N}{s}_{j}}$$

### Lentivirus packaging

HEK293T cells were used to produce lentiviral particles using FuGENE HD Transfection Reagent (Promega, #E2311) following the manufacturer’s protocol. Briefly, 1 day before transfection, HEK293T cells at low passage (< P10) were plated at 100,000 cells per well of a 12-well plate (Cellvis, P12-1.5H-N). At the time of transfection, cells reached a confluency of 70–80%. For lentivirus packaging, a master mix of DNA was prepared in reduced serum media (OptiMEM, Gibco, #31985–070). This master mix contained the lentiviral packaging plasmid pCMV-VSV-G (a gift from Bob Weinberg, Addgene plasmid # 8454) and pCMV-dR8.2 (a gift from Bob Weinberg, Addgene plasmid # 8455). For packaging each virus, the following amounts of each plasmid were mixed: 0.5 μg transfer vector + 0.45 μg pCMV-dR8.2 + 0.05 μg pCMV-VSV-G. Media was changed 24 h post-transfection to fresh DMEM. Lentiviral particles were harvested 60 h after transfection. The viral supernatants were filtered using a 33-mm-diameter sterile syringe filter with a 0.45-µm pore size hydrophilic PVDF membrane (Millipore Sigma, SLHVR33RS) and added to HEK293T cells. The virus was immediately used or stored at − 80 °C. Polybrene (8 μg/mL; Sigma-Aldrich) was supplemented to enhance transduction efficiency.

### CRISPR-Sirius labeling

Plasmids were obtained from Addgene as bacterial stabs and streaked onto LB-ampicillin plates. Upon overnight growth and single-colony selection, a single colony was inoculated into LB-ampicillin liquid culture overnight. Plasmid DNA isolation was performed using QIAprep Spin Miniprep kit (Qiagen, # 27104) following the manufacturer’s protocol. pHAGE-TO-dCas9-P2A-HSA (Addgene plasmid # 121936; http://n2t.net/addgene:121936; RRID: Addgene_121936), pHAGE-EFS-MCP-HALOnls (Addgene plasmid # 121937; http://n2t.net/addgene:121937; RRID: Addgene_121937), and pPUR-hU6-sgRNA-Sirius-8XMS2 (Addgene plasmid # 121942; http://n2t.net/addgene:121942; RRID: Addgene_121942) were gifts from Thoru Pederson. Target sequences for CRISPR-Sirius labeling are listed in Additional file 1.

### CRISPR-Sirius transduction

For live-cell CRISPR-Sirius, HCT116^LMN(B1&B2)−AID^ cells were transfected with CRISPR-dCas9 and donor plasmids using FuGENE HD Transfection Reagent (Promega, #E2311) following the manufacturer’s protocol. Briefly, cells at low passage (< P10) were plated at 100,000 cells per well of a 6-well glass-bottom plate (Cellvis, P06-1.5H-N). Twenty-four hours after plating, 50 μL dCas9, 50 μL MCP-HALOnls, and 100 μL sgRNA lentiviral particles were added to each well. Twenty-four hours after transduction, lentiviral particles were removed by replacing media.

### CRISPR-Sirius transfection

For additional quantification of foci and distances of foci to the nuclear periphery, HCT116^LMN(B1&B2)−AID^ cells were co-transfected with 200 ng MCP-HaloTag, 400 ng of dCas9 plasmid DNA, and 2 µg of plasmid DNA for the desired guide RNAs using Lipofectamine LTX and Plus Reagent. Cells were incubated for 24 h prior to overnight staining with HaloTag-JF646 before fixation and imaging.

### Fluorescence in situ hybridization (FISH)

Cells were trypsinized, resuspended in appropriate growth media, and then plated onto glass coverslips and allowed to adhere for approximately 16 h prior to fixation. Cells were rinsed once in PBS prior to fixation in 4% PFA for 10 min at room temperature. Cells were then washed 3 times in PBS, followed by incubation in PBS with 0.01% Triton X-100 at room temperature 3 times for 3 min, then incubation in 0.5% Triton X-100/1 × PBS at room temperature for 15 min. Cells were then incubated in 20% glycerol/PBS at room temperature for 3 h. Cells were washed in PBS 3 times for 10 min each and then incubated in 0.1 N HCl for 5 min at room temperature. The cells were incubated in 2 × SSC twice for 3 min each before being placed in 50% formamide (Electron Microscopy Sciences)/2 × SSC at room temperature for approximately 18 h. After the addition of FISH probes (Empire Genomics) to the coverslips, slides were heated to 75°C for 2 min before being placed at 37° for approximately 24 h for hybridization. After hybridization, coverslips were washed in 2 × SSC washes at 37° three times for 5 min each, followed by washes in 0.1 × SSC at 60° three times for 5 min each. The coverslips were then washed in 4 × SSC/0.2% Tween-20 three times for 3 min and mounted on microscope slides using either Diamond antifade with DAPI (Invitrogen) or 5 µm of DRAQ5 Fluorescent Probe Solution (Thermo Fisher Scientific, #62251). The following gene-specific FISH probes were used in this study: CEMIP-20-AQ (Chr15:80,779,370–80,951,771), SLCO3A1-20-RE (Chr15:91,853,708–92,172,435), and CYP1A1-20-AQ (Chr15:74,719,542–74,725,528).

### Data and image analysis

We used GraphPad Prism 10.1.1 or Excel for statistical analysis and for making all boxplots. Flow cytometric analysis (FACS) data were analyzed using FlowJo software version 10.6.1. To localize the fluorescent puncta, we used ImageJ software to first generate max projections of the Z-stack images/50 frame single-layer acquisition. Max projected images were then background subtracted using the standard rolling ball algorithm with a radius of 12.0. Processed images were then input into the ThunderSTORM ImageJ plugin with a peak intensity threshold coefficient of 5.0 to locate the fluorescent puncta associated with the CRISPR-Sirius and FISH foci. Puncta coordinates were saved in Microsoft Excel as a.csv file to be input to the Python algorithm. The processed image was then fed into a Python computer vision algorithm that segmented out the nucleus and determined the coordinates for the nuclear periphery as well as the centroid of the nucleus. To quantify the spatial distance to the nuclear center or between foci, only pairs of foci lying in the same focal plane were analyzed. Distances were measured from each fluorescent puncta to the nearest nuclear periphery coordinate as well as the centroid of the segmented nucleus. To account for differences in cell area, the distance from each focus to the centroid was divided by the radius, assuming a circular area. This was further verified by dividing the distance by the nuclear area. To detect foci numbers, maximum intensity projection of Z-series images was performed. For chromosome paint experiments, images were analyzed through imaging processing pipelines utilizing standard tools on Cell Profiler [82] and Fiji [83].

### Coefficient of variation analysis

To assess chromatin compaction through the Coefficient of Variation (CoV) analysis, DAPI-stained cells (see section Fixed-cell immunofluorescence) treated with Auxin (see section “Auxin treatment”) were imaged on a Nikon SoRa Spinning Disk confocal microscope (see section “Confocal imaging”). Following a published workflow [53], we used ImageJ to create masks of each nucleus. The coefficient of variation of individual nuclei was calculated in MATLAB, with CoV = *σ*/*μ*, where *σ* represents the standard deviation of the intensity values and *μ* representing the mean value of intensity of the nucleus.

### RNA-Seq library preparation

Total RNA extraction was performed on samples from colon carcinoma epithelial HCT116 cells and HCT116^LMN(B1&B2)−AID^ cells using the RNeasy Plus Mini Kit (Qiagen, #74134) following the manufacturer’s protocol. The conditions for these samples were control, 12-h auxin, 48-h auxin, and 48-h auxin with 6 days of removal by changing cell culture media. These samples were collected with three biological replicates per condition. RNA-Seq libraries were prepared using the NEBNext Ultra Directional RNA Library Prep Kit for Illumina (New England BioLabs, #E7760), according to the company’s instruction. Library quality was measured using the Qubit 2.0 and Bioanalyzer.

### RNA-Seq data analysis

Bulk mRNA sequencing was conducted in The Genome Analysis and Technology Core in Charlottesville, VA, at the University of Virginia. Paired-end reads were acquired using NextSeq 2000 (75bp) system on high-throughput mode. Reads were aligned to the hg19 genome using HISAT2 and quantified using StringTie. Read counts were normalized and compared for differential gene expression using the DESeq2 package in R. Heatmaps were generated using the pheatmap package. Other plots were generated using the ggplot2 package. We used Metascape (https://metascape.org/gp/index.html#/main/step1) to perform pathway enrichment analysis. The publicly available hg19 DamID track was downloaded from the 4D Nucleome data repository (data.4dnucleome.org) [45, 84]. The tracks are obtained from two independent biological replicates. We used bedtools to compare gene coordinates with the DamID LAD coordinates. We specifically used the “reldist,” “closest,” and “coverage” options of bedtools.

## Quantification and statistical analysis

Statistical analysis was performed using GraphPad Prism 10.1.1 and Microsoft Excel. Pairwise comparisons were calculated on datasets consisting of, at a minimum, biologically independent duplicate samples using two-tailed unpaired *t* test. Experimental data are presented as the mean ± SEM unless otherwise stated in figure legends. The type of statistical test is specified in each case. Significance was calculated by Mann–Whitney test for measuring distances of foci in CRISPR-Sirius and FISH experiments. Multiple comparison corrections were applied for datasets with comparing more than two groups and multiple comparisons. A *P* value of < 0.05 was considered significant. Statistical significance levels are denoted as follows: ns = not significant; *P < 0.05; **P < 0.01; ***P < 0.001; ****P < 0.0001. Sample numbers (# of nuclei, *n*), the number of replicates (*N*), and the type of statistical test used is indicated in figure legends. 

### Supplementary Information

**Additional file 1.** Plasmids for making parental HCT116 cells expressing OsTIR1 (**Table S1**), plasmids for the construction of CRISPR and donor plasmids for tagging (**Table S2**), primers used to generate the sgRNAs for creating the cell lines (**Table S3**), and CRISPR-Sirius labeling information (**Table S4**).

**Additional file 2:** Supplementary Figures [91]. **Fig. S1.** Degradation of B-type lamins increases nuclear area but does not induce apoptosis. **Fig. S2.** The mesoscale structure of chromatin is overall preserved upon B-type lamin degradation. **Fig. S3.** Chromosome occupancy is shifted upon the addition of auxin. **Fig. S4.** Dual-PWS reveals differential higher-order chromatin structure and dynamics upon B-type lamin loss. **Fig. S5.** Chromatin decompaction promotes redistribution of heterochromatic marks. **Fig. S6.** SMLM allows sub-diffraction resolution to assess heterochromatin redistribution without perturbing B-type lamins. **Fig. S7.** Degradation of B-type lamins promotes differential gene expression near LAD boundaries. **Fig. S8.** Degradation of B-type lamins promotes differential gene expression near LAD boundaries. **Fig. S9.** Chromosomes with decreased interaction frequencies had more DEGs outside LADs.

**Additional file 3.** Uncropped western blots of H3K27me3 levels before and after 24-h auxin treatment [90].

**Additional file 4.** Differentially expressed genes for each RNA-seq condition tested (**Table S5**).

**Additional file 5.** Review history.

## Data Availability

RNA sequencing and Hi-C data were submitted to Sequence Read Archive under BioProject Accession number PRJNA1078503 and are available at the following URL: https://www.ncbi.nlm.nih.gov/sra/PRJNA1078503 [85]. Raw and processed Hi-C data are also available as open data in the Dryad repository under a Creative Commons Public Domain License (CC0) and are available at the following URL: https://datadryad.org/stash/share/XCJsTz8vWBTUrbxr5bbGx1eE8wY-Q-2ezW6-mmUVAKk [86]. The original source codes used to generate data in this study are available on GitHub under GNU General Public License v3.0 [87] and Zenodo under a Creative Commons Attribution 4.0 International license [88]. The DamID data that support the findings of this study are from the 4D Nucleome data repository (https://data.4dnucleome.org) [45, 84] (Bas van Steensel, NKI, 2018) under accession number 4DNES24XA7U8 and are available at the following URL: https://data.4dnucleome.org/experiment-set-replicates/4DNES24XA7U8/ [89]. Supplementary figures, microscopy data, and uncropped western blot data that support the findings of this study are available in Figshare [90–96].
