# Supplementary material for: Depletion of lamins B1 and B2 promotes chromatin mobility and induces differential gene expression by a mesoscale-motion-dependent mechanism
Source: Genome Biol. 2024 Mar 22;25:77. doi: 10.1186/s13059-024-03212-y (PMC10958841; doi:10.1186/s13059-024-03212-y)
Supplement: Supplementary file 2 — Additional file 2: Supplementary Figures [91]. Fig. S1. Degradation of B-type lamins increases nuclear area but does not induce apoptosis. Fig. S2. The mesoscale structure of chromatin is overall preserved upon B-type lamin degradation. Fig. S3. Chromosome occupancy is shifted upon the addition of auxin. Fig. S4. Dual-PWS reveals differential higher-order chromatin structure and dynamics upon B-type lamin loss. Fig. S5. Chromatin decompaction promotes redistribution of heterochromatic marks. Fig. S6. SMLM allows sub-diffraction resolution to assess heterochromatin redistribution without perturbing B-type lamins. Fig. S7. Degradation of B-type lamins promotes differential gene expression near LAD boundaries. Fig. S8. Degradation of B-type lamins promotes differential gene expression near LAD boundaries. Fig. S9. Chromosomes with decreased interaction frequencies had more DEGs outside LADs. [file 13059_2024_3212_MOESM2_ESM.pdf]

Supplementary Figures for

Depletion of lamins B1 and B2 promotes chromatin mobility and induces differential gene expression by a mesoscale-motion dependent mechanism

Emily M. Pujadas Liwag, Xiaolong Wei, Nicolas Acosta, Lucas Carter, Jiekun Yang, Luay Almassalha, Surbhi Jain, Ali Daneshkhah, Suhas S.P. Rao, Fidan Seker-Polat, Kyle L. MacQuarrie, Joe Ibarra, Vasundhara Agrawal, Erez Lieberman Aiden, Masato T. Kanemaki, Vadim Backman, Mazhar Adli

Fig. S1

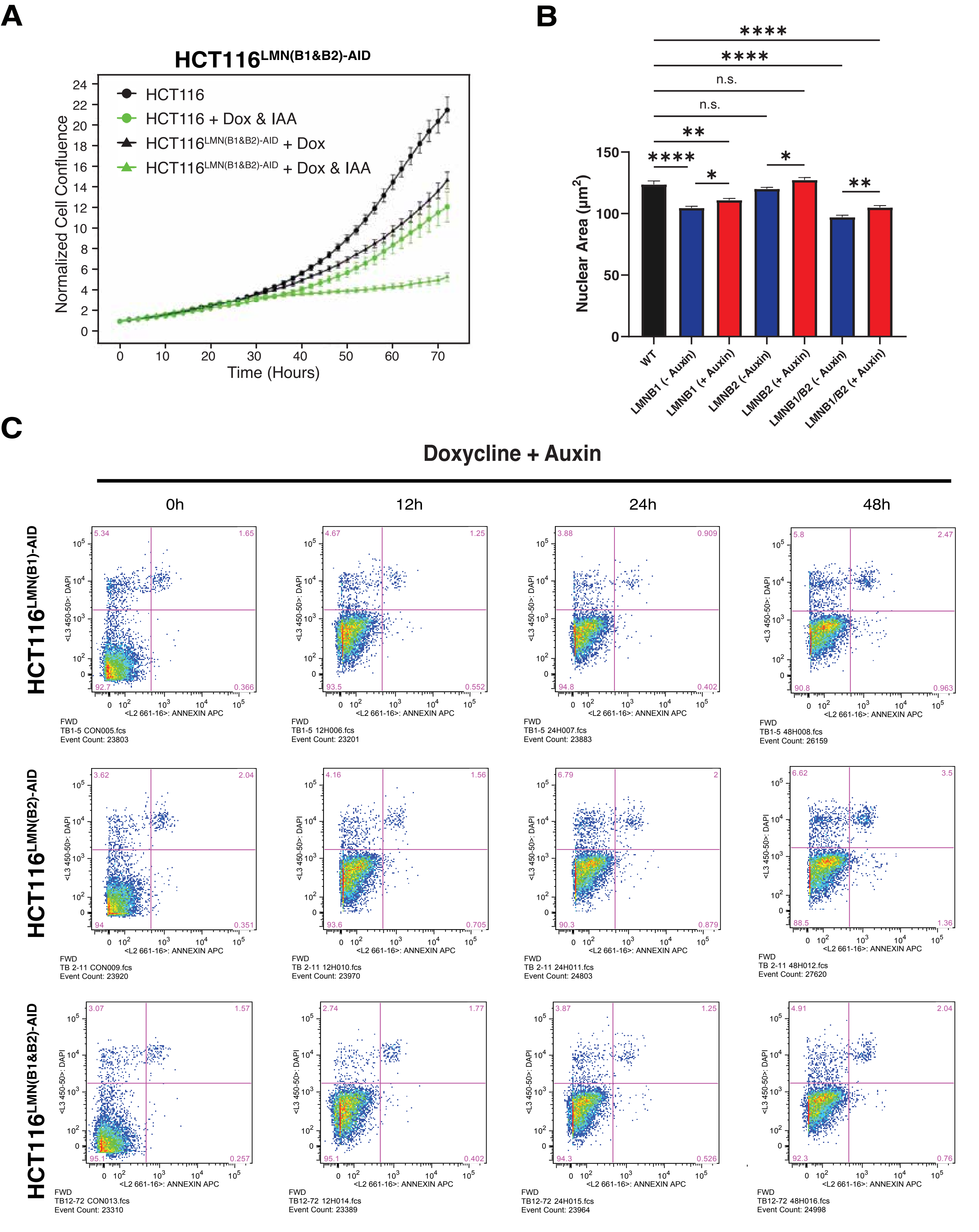

A
HCT116LMN(B1&B2)-AID
HCT116
HCT116 + Dox & IAA
HCT116LMN(B1&B2)-AID + Dox
HCT116LMN(B1&B2)-AID + Dox & IAA
Normalized Cell Confluence
Time (Hours)
B
Nuclear Area (μm2)
n.s.
n.s.
WT
LMNB1 (- Auxin)
LMNB1 (+ Auxin)
LMNB2 (-Auxin)
LMNB2 (+ Auxin)
LMNB1/B2 (- Auxin)
LMNB1/B2 (+ Auxin)
C
Doxycline + Auxin
0h
12h
24h
48h
HCT116LMN(B1)-AID
HCT116LMN(B2)-AID
HCT116LMN(B1&B2)-AID
<L3 450-50>: DAPI
<L2 661-16>: ANNEXIN APC
FWD
TB1-5 CON005.fcs
Event Count: 23803
TB1-5 12H006.fcs
Event Count: 23201
TB1-5 24H007.fcs
Event Count: 23883
TB1-5 48H008.fcs
Event Count: 26159
TB 2-11 CON009.fcs
Event Count: 23920
TB 2-11 12H010.fcs
Event Count: 23970
TB 2-11 24H011.fcs
Event Count: 24803
TB 2-11 48H012.fcs
Event Count: 27620
TB12-72 CON013.fcs
Event Count: 23310
TB12-72 12H014.fcs
Event Count: 23389
TB12-72 24H015.fcs
Event Count: 23964
TB12-72 48H016.fcs
Event Count: 24998

Fig. S1

Degradation of B-type lamins increases nuclear area but does not induce apoptosis.

A. Time-course imaging in a 96-well plate using the Incucyte Live-Cell Analysis System to assess the growth rate of HCT116 and HCT116$^{LMN(B1\&B2)-AID}$ cells. Growth rate data every 2 hours for a total of 72 hours (normalized to the 0-hour condition). We measured growth rates with and without the addition of doxycycline to induce TIR1, as well as with and without the addition of both doxycycline and auxin to degrade B-type lamins. Error bars: SEM. Data was compiled from three biological replicates (N = 3).

B. Quantification of DAPI-stained area from fixed-cell immunofluorescence (Mean + SEM). Data was compiled from two independent biological replicates (N = 2). Significance was calculated by one-way ANOVA with Šidák's test for multiple comparisons (n.s. = not significant; *<0.05; **<0.01). WT HCT116 (Control n = 66), LMNB1 (Control n = 257; Auxin n = 289), LMNB2 (Control n = 272; Auxin n = 196), LMNB1/B2 (Control n = 209; Auxin n = 257).

C. Auxin treatment to induce acute depletion of B-type lamins in HCT116$^{LMN(B1)-AID}$ cells, HCT116$^{LMN(B2)-AID}$ cells, and HCT116$^{LMN(B1\&B2)-AID}$ cells does not substantially induce cell death. Viable, in-tact cells are present in the bottom left quadrant, dead cells are shown in the top right quadrant, and apoptotic cells are shown in the bottom right quadrant in the plots. At least 20,000 events were recorded during the experiment.

Fig. S2

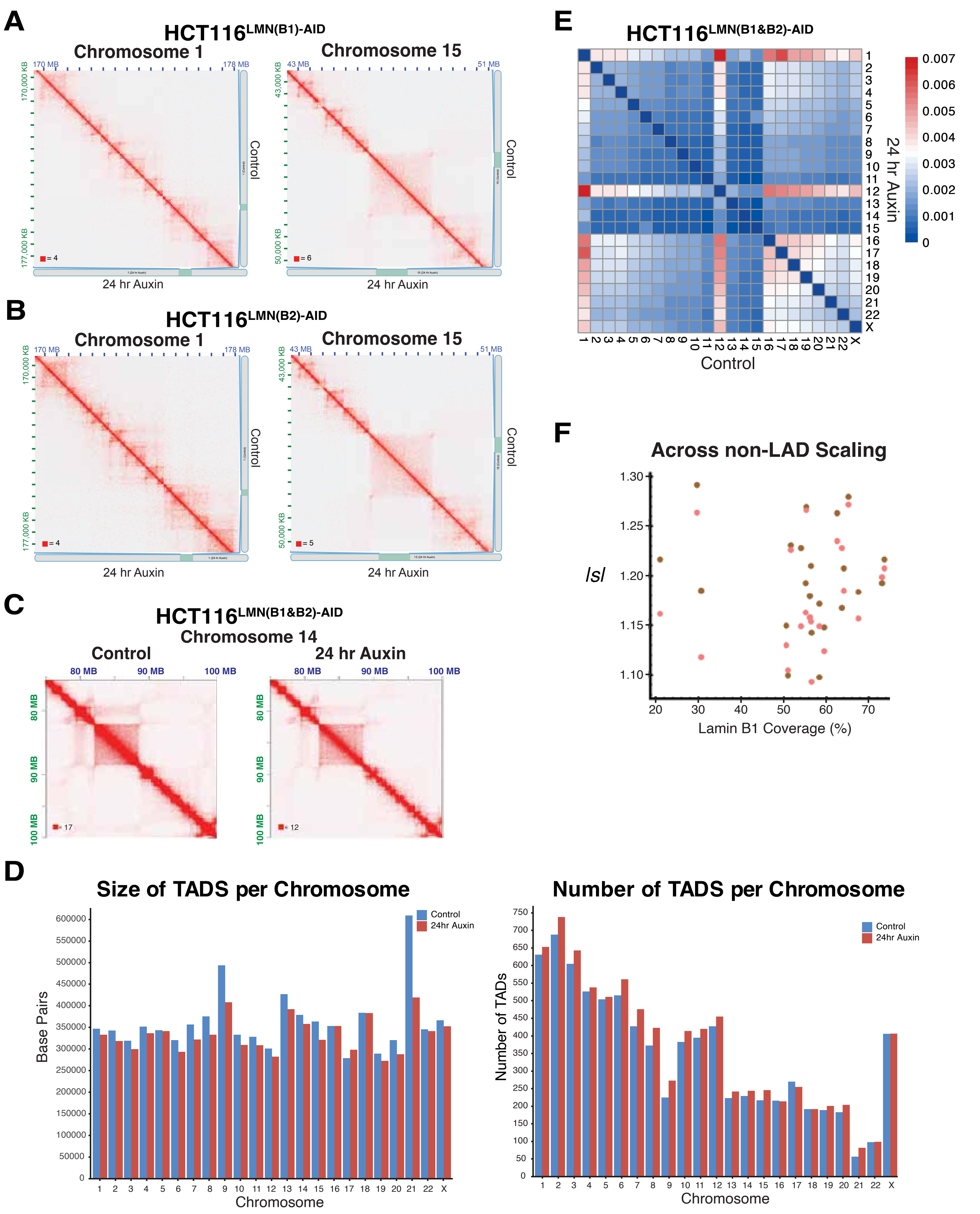

A
HCT116LMN(B1)-AID
Chromosome 1
Chromosome 15
Control
24 hr Auxin
B
HCT116LMN(B2)-AID
Chromosome 1
Chromosome 15
Control
24 hr Auxin
C
HCT116LMN(B1&B2)-AID
Chromosome 14
Control
24 hr Auxin
80 MB
90 MB
100 MB
D
Size of TADS per Chromosome
Base Pairs
Chromosome
Control
24hr Auxin
Number of TADS per Chromosome
Number of TADs
Chromosome
E
HCT116LMN(B1&B2)-AID
24 hr Auxin
Control
F
Across non-LAD Scaling
|s|
Lamin B1 Coverage (%)

Fig. S2

The mesoscale structure of chromatin is overall preserved upon B-type lamin degradation.

A. The normalized Hi-C trans-interaction matrices for chromosomes 1 and 15 in the control and 24-hour auxin treatment are shown for HCT116$^{LMN(B1)\text{-}AID}$ cells. 15 kb resolution.
B. Normalized Hi-C trans-interaction matrices for chromosomes 1 and 15 in the control and 24-hour auxin treatment conditions for HCT116$^{LMN(B2)\text{-}AID}$ cells. 15 kb resolution.
C. Normalized Hi-C interaction matrices for HCT116$^{LMN(B12\&B2)\text{-}AID}$ cells for chromosome 14 at 50 kb resolution from 75 Mb to 100 Mb.
D. TAD sizes for each chromosome for HCT116$^{LMN(B1\&B2)\text{-}AID}$ cells in the control and 24-hour auxin treatment conditions.
E. Normalized Hi-C trans-interaction matrix for the mean contact frequency, which is the ratio of observed/ expected interchromosomal contact frequency.
F. Scatter plot for the relationship between lamin B1 coverage and |*s*| in non-LAD segments.

Fig. S3

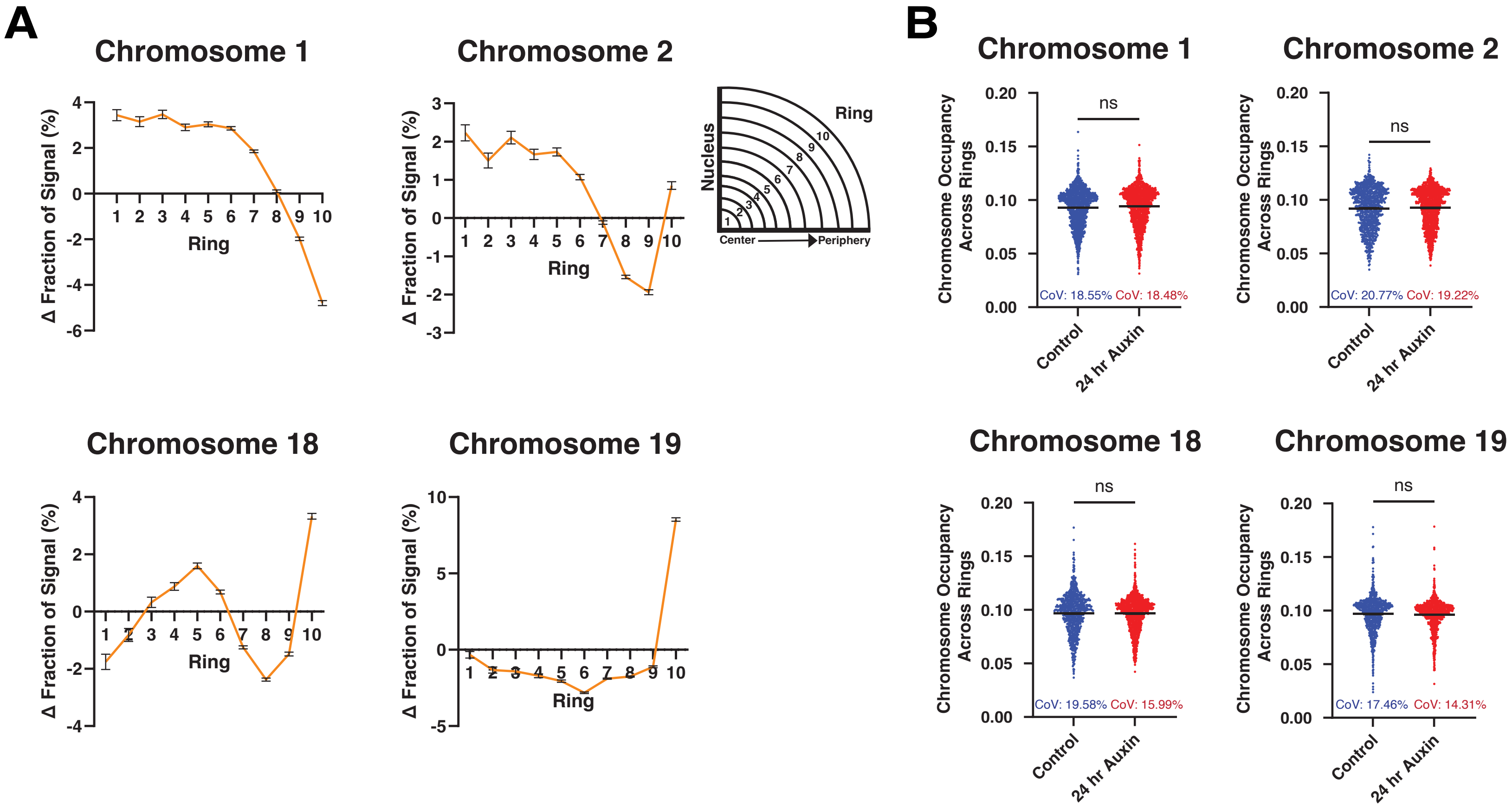

Fig. S3

Chromosome occupancy is shifted upon the addition of auxin.

A. Percent change between concentric rings for each chromosome and condition. Error bars: SD.
B. Chromosome occupancy of nuclei in compiled rings before and after 24-hour auxin treatment. The coefficient of variation (CoV) for each condition and chromosome is obtained by dividing the standard by the mean and is expressed as a percentage. Data was compiled from three independent biological replicates (N = 3). Significance was calculated by unpaired t test (n.s. = not significant). Chr. 1 (Control n = 1660; Auxin n = 1960), Chr. 2 (Control n = 1010; Auxin n = 1740), Chr. 18 (Control n = 890; Auxin n = 1480), Chr. 19 (Control n = 830; Auxin n = 1200). The violin plots extend from the minimum to the maximum value. The line in the middle of each plot is the median value of the distribution, and the lines above and below are the third and first quartiles, respectively. Each dot represents one cell.

Fig. S4

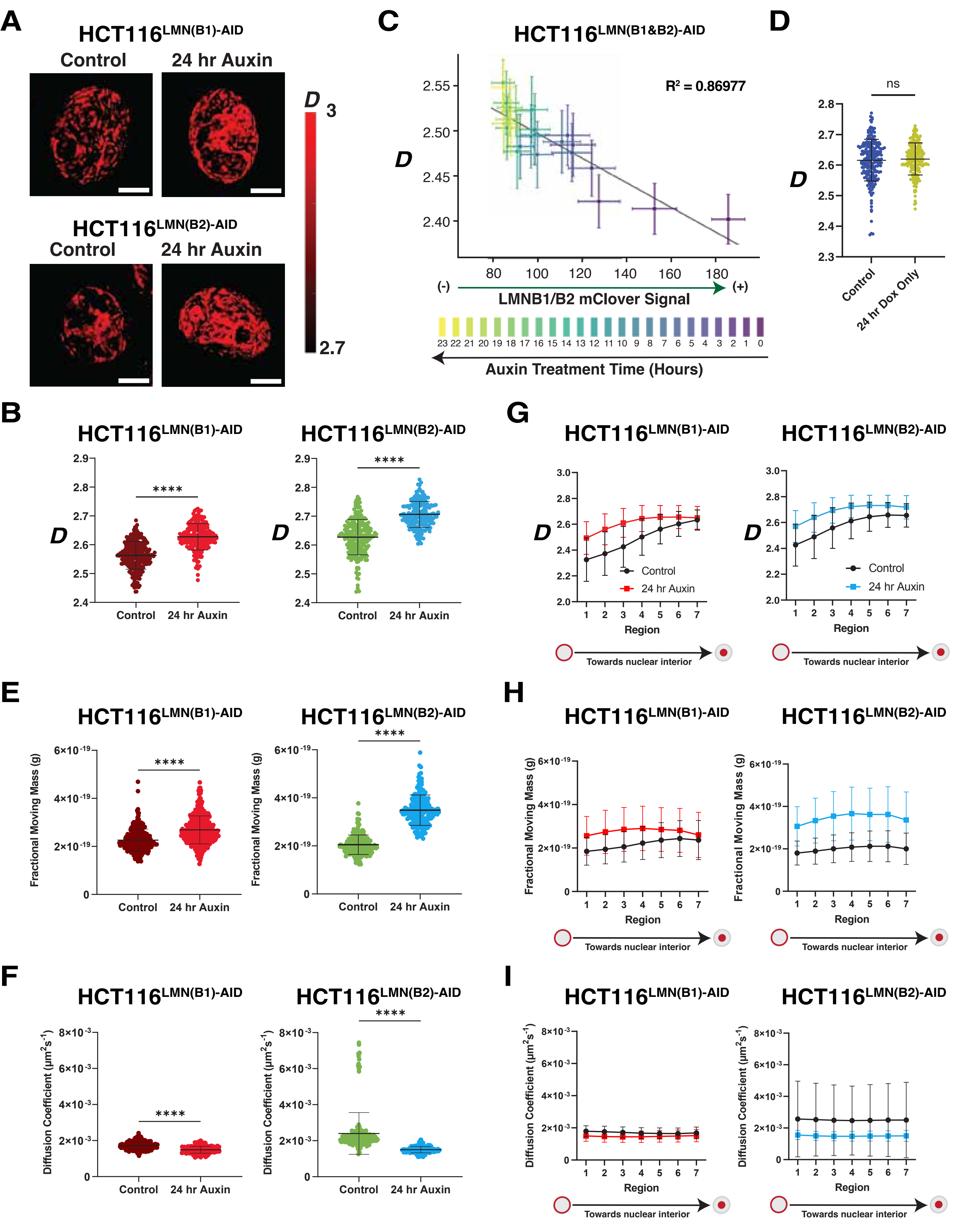

A
HCT116LMN(B1)-AID
Control
24 hr Auxin
HCT116LMN(B2)-AID
Control
24 hr Auxin
D
3
2.7
C
HCT116LMN(B1&B2)-AID
R² = 0.86977
D
LMNB1/B2 mClover Signal
(-)
(+)
23 22 21 20 19 18 17 16 15 14 13 12 11 10 9 8 7 6 5 4 3 2 1 0
Auxin Treatment Time (Hours)
D
ns
Control
24 hr Dox Only
B
HCT116LMN(B1)-AID
HCT116LMN(B2)-AID
****
Control
24 hr Auxin
G
Control
24 hr Auxin
Region
Towards nuclear interior
E
Fractional Moving Mass (g)
H
F
Diffusion Coefficient (μm²s⁻¹)
I

Fig. S4

Dual-PWS reveals differential higher-order chromatin structure and dynamics upon B-type lamin loss.

A. Representative PWS images of HCT116$^{LMN(B1)-AID}$ cells and HCT116$^{LMN(B2)-AID}$ cells before and after 24-hour auxin treatment. Scale bar = 5 µm. Data are representative of three independent biological replicates (N = 3).
B. Violin plots for D in HCT116$^{LMN(B1)-AID}$ cells and HCT116$^{LMN(B2)-AID}$ cells before and after 24-hour auxin treatment.
C. Live-cell PWS 24-hour auxin treatment time course in HCT116$^{LMN(B1\&B2)-AID}$ cells. Error bars: SD. Data are representative of three independent biological replicates (N = 3; n = 634).
D. Doxycycline treatment does not alter D in HCT116$^{LMN(B1\&B2)-AID}$ cells. Data was compiled from three independent biological replicates (N = 3). Significance was calculated by unpaired t test with Welch's correction applied (n.s. = not significant). (Control n = 507; Auxin n = 519).
E. Violin plots for fractional moving mass in HCT116$^{LMN(B1)-AID}$ cells and HCT116$^{LMN(B2)-AID}$ cells before and after 24-hour auxin treatment.
F. Violin plots for diffusion coefficient in HCT116$^{LMN(B1)-AID}$ cells and HCT116$^{LMN(B2)-AID}$ cells before and after 24-hour auxin treatment.
G. Regional PWS measurements of D in HCT116$^{LMN(B1)-AID}$ cells and HCT116$^{LMN(B2)-AID}$ cells before and after 24-hour auxin treatment.
H. Regional PWS measurements of fractional moving mass in HCT116$^{LMN(B1)-AID}$ cells and HCT116$^{LMN(B2)-AID}$ cells before and after 24-hour auxin treatment.
I. Regional PWS measurements of diffusion coefficient for HCT116$^{LMN(B1)-AID}$ cells and HCT116$^{LMN(B2)-AID}$ cells before and after 24-hour auxin treatment.

For A-B and E-I: Data was compiled from three independent biological replicates (N = 3). Significance was calculated by unpaired t test with Welch's correction applied (****< 0.0001). Lamin B1 (Control n = 694; Auxin n = 585), Lamin B2 (Control n = 568; Auxin n = 525). Violin plots extend from the minimum to the maximum value. The line in the middle of each plot is the median value of the distribution, and the lines above and below are the third and first quartiles, respectively. Each dot represents one cell.

Fig. S5

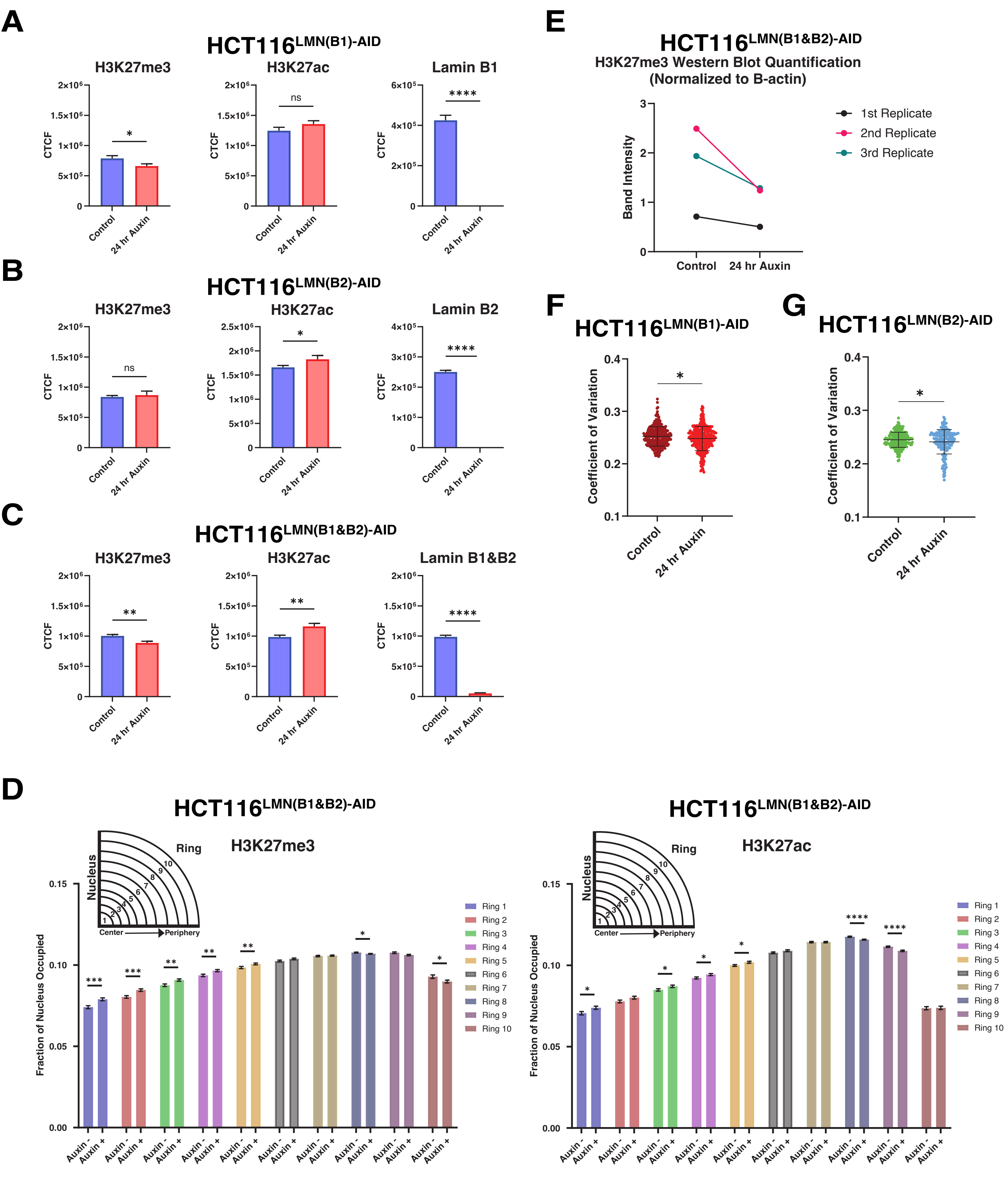

A
HCT116LMN(B1)-AID
H3K27me3
H3K27ac
Lamin B1
CTCF
Control
24 hr Auxin
ns
*
****
B
HCT116LMN(B2)-AID
H3K27me3
H3K27ac
Lamin B2
C
HCT116LMN(B1&B2)-AID
H3K27me3
H3K27ac
Lamin B1&B2
**
D
HCT116LMN(B1&B2)-AID
H3K27me3
H3K27ac
Ring
Nucleus
Center
Periphery
Fraction of Nucleus Occupied
Ring 1
Ring 2
Ring 3
Ring 4
Ring 5
Ring 6
Ring 7
Ring 8
Ring 9
Ring 10
Auxin -
Auxin +
E
HCT116LMN(B1&B2)-AID
H3K27me3 Western Blot Quantification
(Normalized to B-actin)
Band Intensity
1st Replicate
2nd Replicate
3rd Replicate
Control
24 hr Auxin
F
HCT116LMN(B1)-AID
Coefficient of Variation
G
HCT116LMN(B2)-AID

Fig. S5

Chromatin decompaction promotes redistribution of heterochromatic marks.

A. Bar plots of H3K27me3, H3K27ac, and Lamin B1 corrected total cell fluorescence in HCT116$^{LMN(B1)\text{-}AID}$ cells before and after 24-hour auxin treatment (Mean + SEM). (Control n = 281; 24-hr Auxin n = 193).
B. Bar plots of H3K27me3, H3K27ac, and Lamin B2 corrected total cell fluorescence in HCT116$^{LMN(B2)\text{-}AID}$ cells before and after 24-hour auxin treatment (Mean + SEM). (Control n = 249; 24-hr Auxin n = 152).
C. Bar plots of H3K27me3, H3K27ac, and Lamin B1&B2 corrected total cell fluorescence in HCT116$^{LMN(B1\&B2)\text{-}AID}$ cells before and after 24-hour auxin treatment (Mean + SEM). (Control n = 383; 24-hr Auxin n = 367).
D. Graphing of the average fraction and SEM of H3K27me3 and H3K27ac fluorescent signals present in each one of ten concentric nuclear rings. Data was compiled from two independent biological replicates (N = 2). Significance was calculated by unpaired t test (*< 0.05; **< 0.01; ***< 0.001; ****< 0.0001). (H3K27me3 (Control n = 455; Auxin n = 510), H3K27ac (Control n = 448; Auxin n = 447)).
E. Western blot analysis for H3K27me3 in HCT116$^{LMN(B1\&B2)\text{-}AID}$ cells. GAPDH was used as a loading control. Three independent biological replicates (N = 3) are shown.
F. Coefficient of variation plot for HCT116$^{LMN(B1)\text{-}AID}$ cells showing chromatin decompaction upon 24 hours of auxin treatment. (Control n = 770; Auxin n = 492).
G. Coefficient of variation plot for HCT116$^{LMN(B2)\text{-}AID}$ cells. (Control n = 650; Auxin n = 393).

For A-C and F-G: Data was compiled from three independent biological replicates (N = 3). Significance was calculated by unpaired t test (n.s. = not significant; *<0.05; **<0.01; ***<0.001; ****<0.0001). For F-G: Violin plots extend from the minimum to the maximum value. The line in the middle of each plot is the median value of the distribution, and the lines above and below are the third and first quartiles, respectively.

Fig. S6

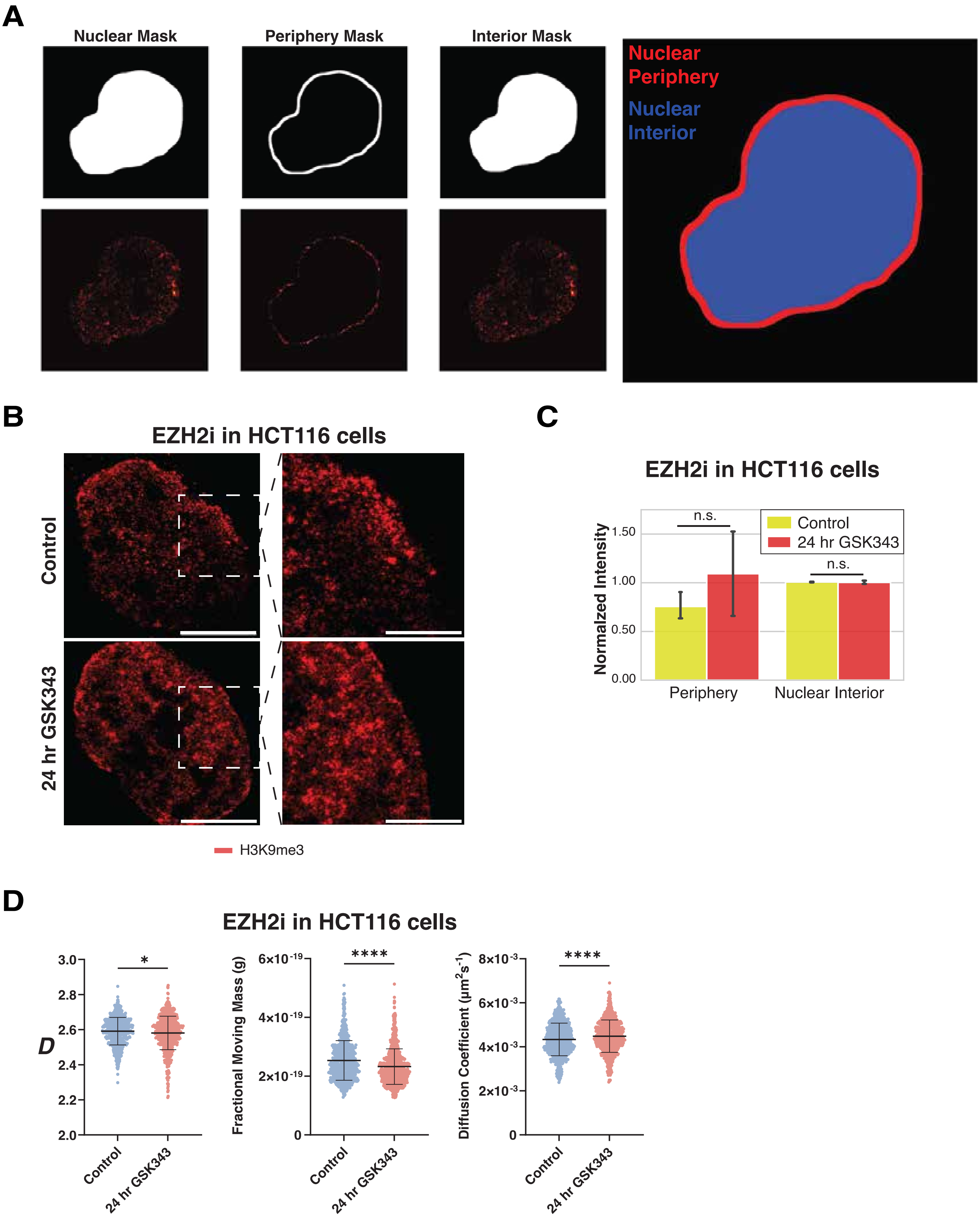
A
Nuclear Mask
Periphery Mask
Interior Mask
Nuclear Periphery
Nuclear Interior
B
EZH2i in HCT116 cells
Control
24 hr GSK343
H3K9me3
C
EZH2i in HCT116 cells
Normalzed Intensity
n.s.
n.s.
Control
24 hr GSK343
1.50
1.00
0.50
0.00
Periphery
Nuclear Interior
D
EZH2i in HCT116 cells
D
3.0
2.8
2.6
2.4
2.2
2.0
*
Fractional Moving Mass (g)
6x10-19
4x10-19
2x10-19
0
****
Diffusion Coefficient (μm2s-1)
8x10-3
6x10-3
4x10-3
2x10-3
0
****
Control
24 hr GSK343

Fig. S6

SMLM allows sub-diffraction resolution to assess heterochromatin redistribution without perturbing B-type lamins.

A. Example of Normalized STORM Intensity (NSI) analysis pipeline. Top: Representative images of binary masks used to specify the regions of interest for NSI analysis. Bottom: Representative masked HCT116$^{LMN(B1\&B2)-AID}$ nucleus corresponding to the binary masks above. Right: Overlay of the nuclear interior and periphery masks in blue and red, respectively.
B. STORM images of HCT116 cells after 24-hour GSK343 treatment (10 μM) compared to the vehicle control (DMSO). Scale bars = 5 μm, Scale bars (zoom) = 2 μm.
C. Quantification of NSI after EZH2 inhibition. Error bars: SD. Significance was calculated by unpaired t test (n.s. = not significant). (Control n = 4; GSK343 n = 5).
D. Violin plots for D, fractional moving mass, and the diffusion coefficient in HCT116 cells after 24-hour GSK343 treatment (10mM) compared to the vehicle control (DMSO). Data was compiled from three independent biological replicates (N = 3). Significance was calculated by unpaired t test (*<0.05; ****< 0.0001). (Control n = 707; Auxin n = 807). The truncated violin plots extend from the minimum to the maximum value. The line in the middle of each plot is the median value of the distribution, and the lines above and below are the third and first quartiles, respectively. Each dot represents one cell.

Fig. S7

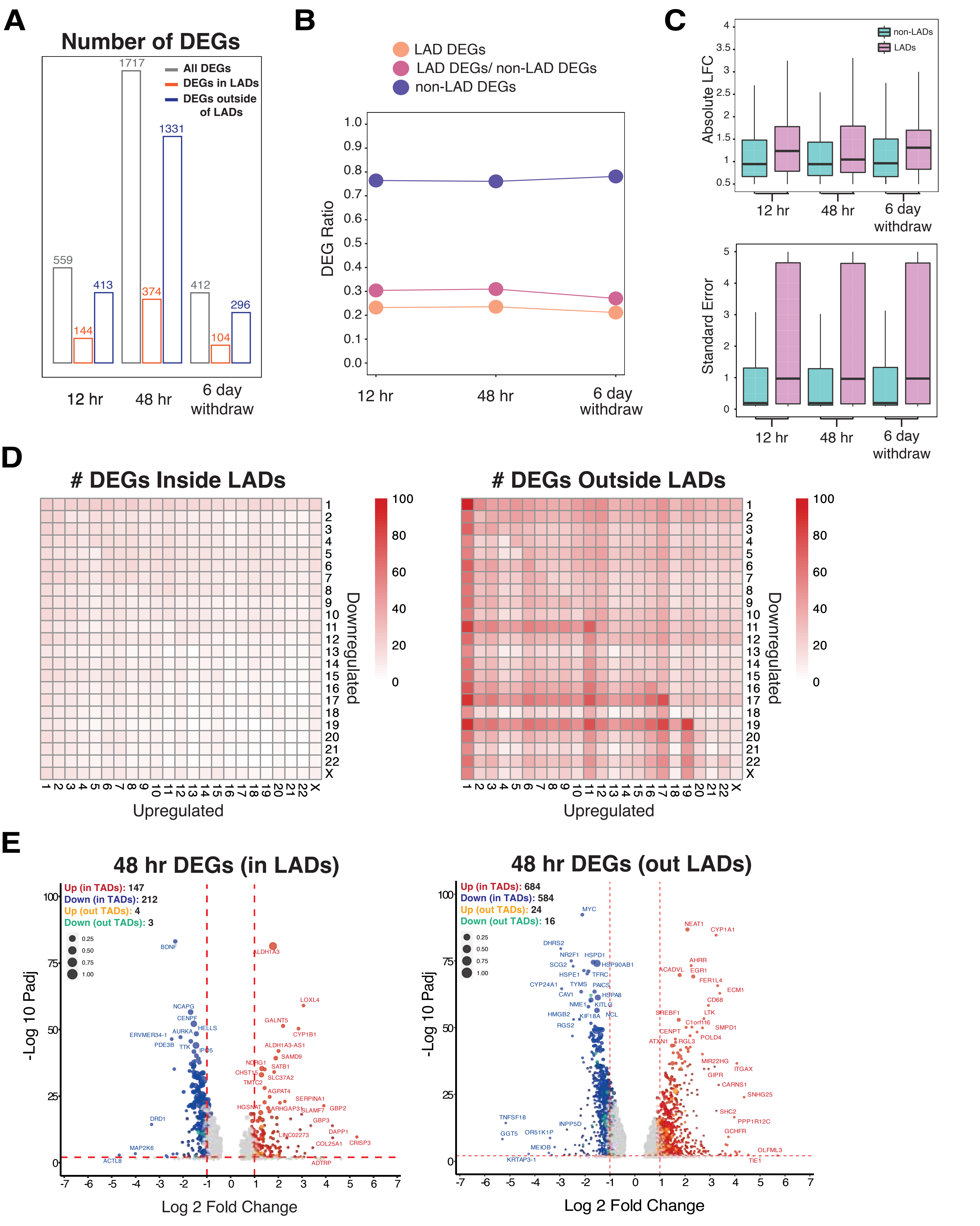

A
Number of DEGs
All DEGs
DEGs in LADs
DEGs outside of LADs
559
144
413
1717
374
1331
412
104
296
12 hr
48 hr
6 day withdraw
B
LAD DEGs
LAD DEGs/ non-LAD DEGs
non-LAD DEGs
DEG Ratio
1.0
0.9
0.8
0.7
0.6
0.5
0.4
0.3
0.2
0.1
0.0
12 hr
48 hr
6 day withdraw
C
non-LADs
LADs
Absolute LFC
4
3.5
3
2.5
2
1.5
1
0.5
12 hr
48 hr
6 day withdraw
Standard Error
5
4
3
2
1
0
12 hr
48 hr
6 day withdraw
D
DEGs Inside LADs
DEGs Outside LADs
1 2 3 4 5 6 7 8 9 10 11 12 13 14 15 16 17 18 19 20 21 22 X
Downregulated
Upregulated
100
80
60
40
20
0
E
48 hr DEGs (in LADs)
Up (in TADs): 147
Down (in TADs): 212
Up (out TADs): 4
Down (out TADs): 3
0.25
0.50
0.75
1.00
BDNF
ALDH1A3
LOXL4
NCAPG
CENPF
HELLS
AURKA
ERVMER34-1
PDE3B
TTK
IPO5
GALNT5
CYP1B1
ALDH1A3-AS1
SAMD9
NDRG1
SATB1
CHST15
SLC37A2
TMTC2
AGPAT4
SERPINA1
HGSNAT
ARHGAP31
SLAMF7
GBP2
GBP3
DRD1
LINC02273
DAPP1
COL25A1
CRISP3
MAP2K6
ACTL8
ADTRP
-Log 10 Padj
Log 2 Fold Change
48 hr DEGs (out LADs)
Up (in TADs): 684
Down (in TADs): 584
Up (out TADs): 24
Down (out TADs): 16
MYC
NEAT1
CYP1A1
DHRS2
NR2F1
HSPD1
SCG2
HSP90AB1
HSPE1
TFRC
AHRR
ACADVL
EGR1
FER1L4
ECM1
CYP24A1
TYMS
PAICS
CAV1
HSPA8
NME1
KITLG
CD68
HMGB2
KIF18A
NCL
SREBF1
LTK
RGS2
C1orf116
SMPD1
CENPT
POLD4
ATXN1
RGL3
MIR22HG
ITGAX
GIPR
CARNS1
SNHG25
TNFSF18
SHC2
INPP5D
PPP1R12C
GGT5
OR51K1P
GCHFR
MEIOB
OLFML3
KRTAP3-1
TIE1
-Log 10 Padj
Log 2 Fold Change

Fig. S7

Degradation of B-type lamins promotes differential gene expression near LAD boundaries.

A. Bar plot showing the number of all DEGs compared to the number of DEGs within versus outside of LAD boundaries defined by publicly available DamID data for all three auxin treatment conditions (adjusted P value < 0.01 and absolute log fold change > 1).
B. Dot plot showing the ratios of DEGs within LADs, outside of LADs, and all DEGs.
C. Box plot showing the absolute log fold change and standard error for DEGs within LADs and outside of LADs for all three auxin treatment conditions.
D. Heatmaps showing the number of DEGs within and outside of LADs in relation to their upregulation or downregulation.
E. Volcano plots showing DEGs within and outside of LADs in relation to TADs after 48 hours of auxin treatment (adjusted P value < 0.01 and absolute log fold change > 1). “Up” refers to upregulated genes and “Down” refers to downregulated genes.

Fig. S8

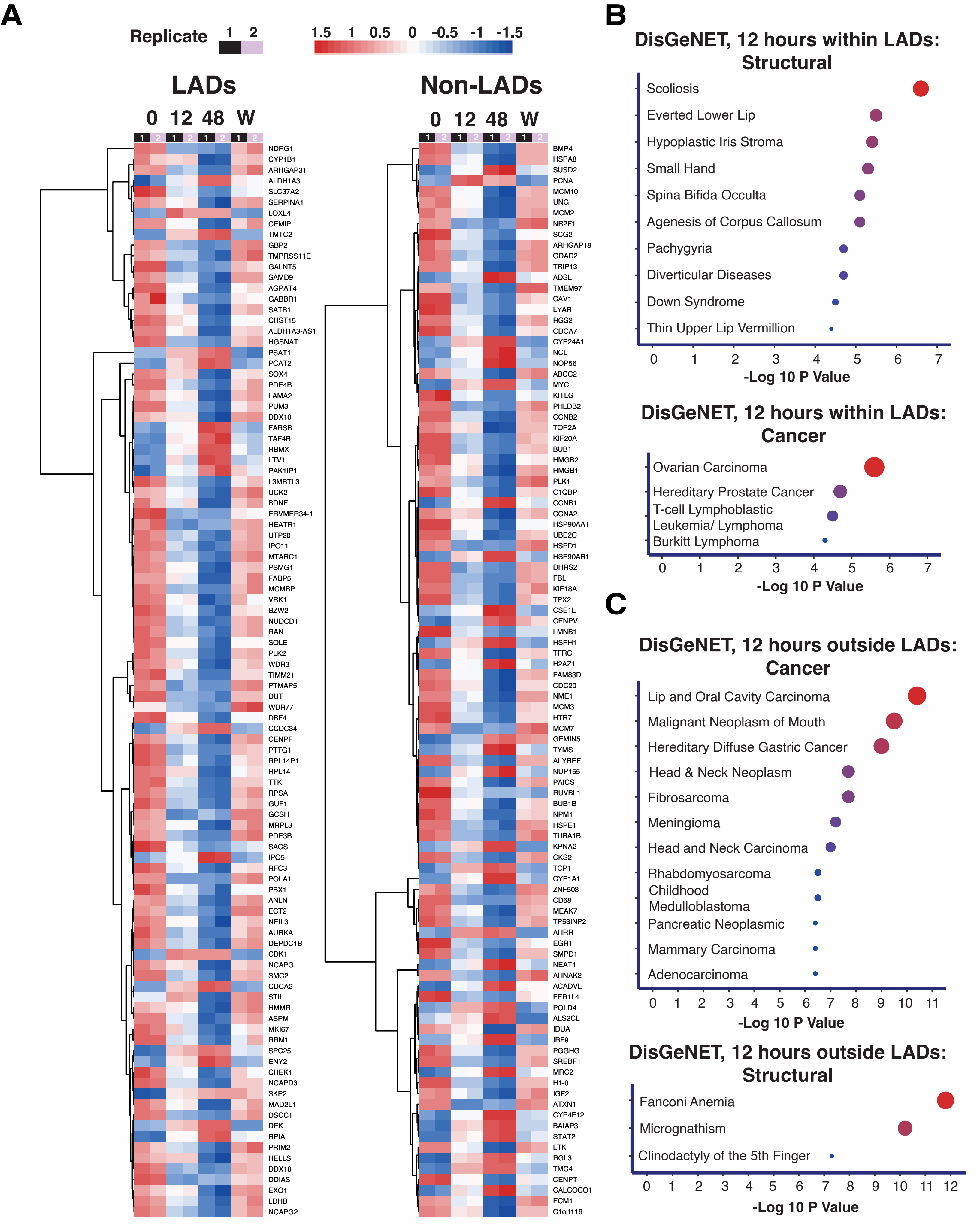

A
Replicate 1 2
1.5 1 0.5 0 -0.5 -1 -1.5
LADs
0 12 48 W
NDRG1
CYP1B1
ARHGAP31
ALDH1A3
SLC37A2
SERPINA1
LOXL4
CEMIP
TMTC2
GBP2
TMPRSS11E
GALNT5
SAMD9
AGPAT4
GABBR1
SATB1
CHST15
ALDH1A3-AS1
HGSNAT
PSAT1
PCAT2
SOX4
PDE4B
LAMA2
PUM3
DDX10
FARSB
TAF4B
RBMX
LTV1
PAK1IP1
L3MBTL3
UCK2
BDNF
ERVMER34-1
HEATR1
UTP20
IPO11
MTARC1
PSMG1
FABP5
MCMBP
VRK1
BZW2
NUDCD1
RAN
SQLE
PLK2
WDR3
TIMM21
PTMAP5
DUT
WDR77
DBF4
CCDC34
CENPF
PTTG1
RPL14P1
RPL14
TTK
RPSA
GUF1
GCSH
MRPL3
PDE3B
SACS
IPO5
RFC3
POLA1
PBX1
ANLN
ECT2
NEIL3
AURKA
DEPDC1B
CDK1
NCAPG
SMC2
CDCA2
STIL
HMMR
ASPM
MKI67
RRM1
SPC25
ENY2
CHEK1
NCAPD3
SKP2
MAD2L1
DSCC1
DEK
RPIA
PRIM2
HELLS
DDX18
DDIAS
EXO1
LDHB
NCAPG2
Non-LADs
0 12 48 W
BMP4
HSPA8
SUSD2
PCNA
MCM10
UNG
MCM2
NR2F1
SCG2
ARHGAP18
ODAD2
TRIP13
ADSL
TMEM97
CAV1
LYAR
RGS2
CDCA7
CYP24A1
NCL
NOP56
ABCC2
MYC
KITLG
PHLDB2
CCNB2
TOP2A
KIF20A
BUB1
HMGB2
HMGB1
PLK1
C1QBP
CCNB1
CCNA2
HSP90AA1
UBE2C
HSPD1
HSP90AB1
DHRS2
FBL
KIF18A
TPX2
CSE1L
CENPV
LMNB1
HSPH1
TFRC
H2AZ1
FAM83D
CDC20
NME1
MCM3
HTR7
MCM7
GEMIN5
TYMS
ALYREF
NUP155
PAICS
RUVBL1
BUB1B
NPM1
HSPE1
TUBA1B
KPNA2
CKS2
TCP1
CYP1A1
ZNF503
CD68
MEAK7
TP53INP2
AHRR
EGR1
SMPD1
NEAT1
AHNAK2
ACADVL
FER1L4
POLD4
ALS2CL
IDUA
IRF9
PGGHG
SREBF1
MRC2
H1-0
IGF2
ATXN1
CYP4F12
BAIAP3
STAT2
LTK
RGL3
TMC4
CENPT
CALCOCO1
ECM1
C1orf116
B
DisGeNET, 12 hours within LADs: Structural
Scoliosis
Everted Lower Lip
Hypoplastic Iris Stroma
Small Hand
Spina Bifida Occulta
Agenesis of Corpus Callosum
Pachygyria
Diverticular Diseases
Down Syndrome
Thin Upper Lip Vermillion
0 1 2 3 4 5 6 7
-Log 10 P Value
DisGeNET, 12 hours within LADs: Cancer
Ovarian Carcinoma
Hereditary Prostate Cancer
T-cell Lymphoblastic Leukemia/ Lymphoma
Burkitt Lymphoma
0 1 2 3 4 5 6 7
-Log 10 P Value
C
DisGeNET, 12 hours outside LADs: Cancer
Lip and Oral Cavity Carcinoma
Malignant Neoplasm of Mouth
Hereditary Diffuse Gastric Cancer
Head & Neck Neoplasm
Fibrosarcoma
Meningioma
Head and Neck Carcinoma
Rhabdomyosarcoma
Childhood Medulloblastoma
Pancreatic Neoplasmic
Mammary Carcinoma
Adenocarcinoma
0 1 2 3 4 5 6 7 8 9 10 11
-Log 10 P Value
DisGeNET, 12 hours outside LADs: Structural
Fanconi Anemia
Micrognathism
Clinodactyly of the 5th Finger
0 1 2 3 4 5 6 7 8 9 10 11 12
-Log 10 P Value

Fig. S8

Degradation of B-type lamins promotes differential gene expression near LAD boundaries.

A. Heatmaps showing the top 100 DEGs within versus outside of LAD boundaries upon 48 hours of auxin treatment defined by publicly available DamID data (adjusted P value < 0.01 and absolute log fold change > 1).
B. DisGeNET results indicate terms upon 12 hours of auxin treatment within LADs related to structural changes and cancer.
C. DisGeNET results indicate terms upon 12 hours of auxin treatment outside of LADs related to cancer and structural changes.

Fig. S9

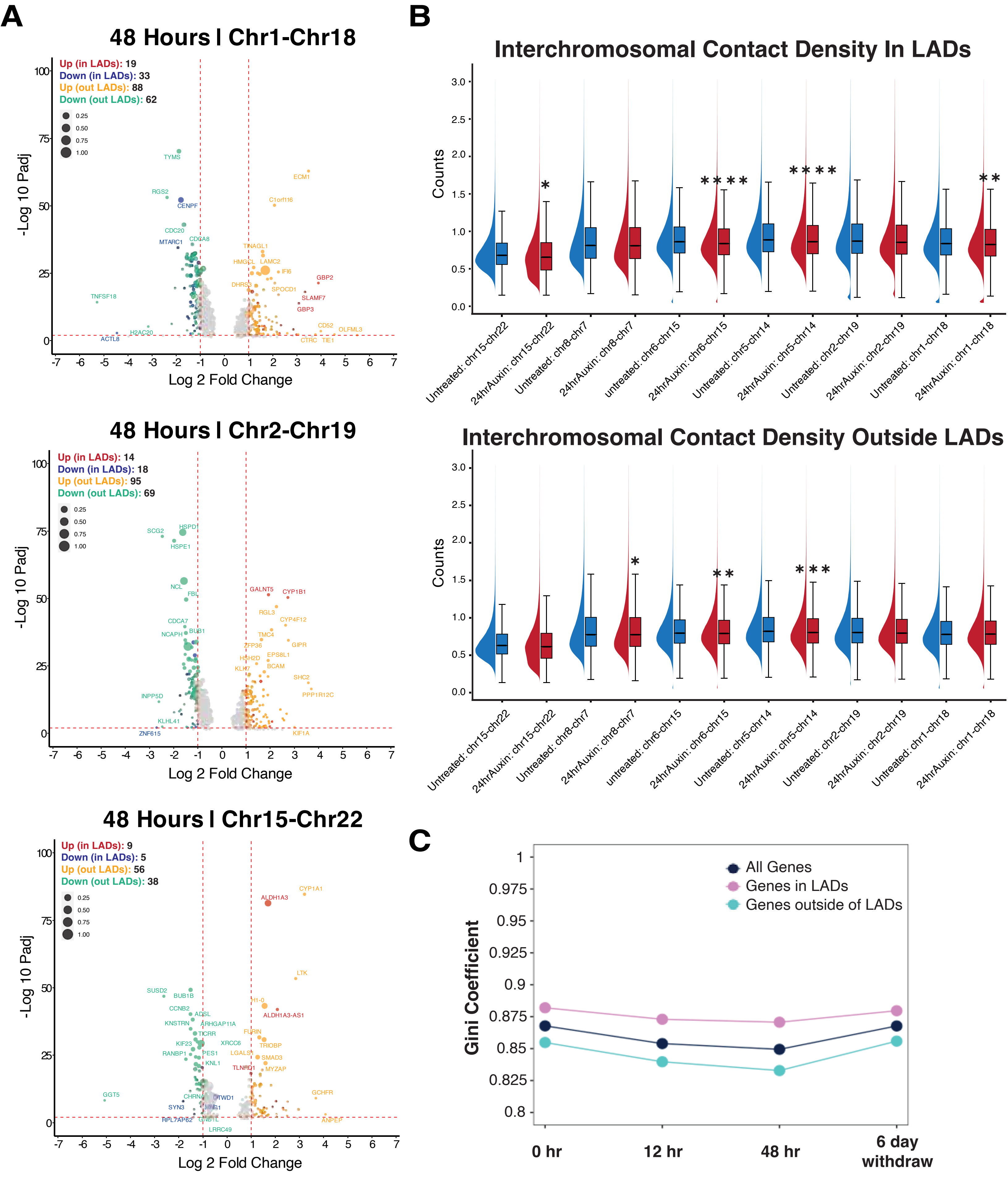
A
48 Hours | Chr1-Chr18
Up (in LADs): 19
Down (in LADs): 33
Up (out LADs): 88
Down (out LADs): 62
-Log 10 Padj
Log 2 Fold Change
48 Hours | Chr2-Chr19
Up (in LADs): 14
Down (in LADs): 18
Up (out LADs): 95
Down (out LADs): 69
-Log 10 Padj
Log 2 Fold Change
48 Hours | Chr15-Chr22
Up (in LADs): 9
Down (in LADs): 5
Up (out LADs): 56
Down (out LADs): 38
-Log 10 Padj
Log 2 Fold Change
B
Interchromosomal Contact Density In LADs
Counts
Untreated: chr15-chr22
24hrAuxin: chr15-chr22
Untreated: chr8-chr7
24hrAuxin: chr8-chr7
untreated: chr6-chr15
24hrAuxin: chr6-chr15
Untreated: chr5-chr14
24hrAuxin: chr5-chr14
Untreated: chr2-chr19
24hrAuxin: chr2-chr19
Untreated: chr1-chr18
24hrAuxin: chr1-chr18
Interchromosomal Contact Density Outside LADs
Counts
C
All Genes
Genes in LADs
Genes outside of LADs
Gini Coefficient
0 hr
12 hr
48 hr
6 day withdraw

Fig. S9

Chromosomes with decreased interaction frequencies had more DEGs outside LADs.

A. Volcano plots showing DEGs specific to chromosome pairs with decreased interaction frequency within and outside of LADs 48 hours of auxin treatment (adjusted P value < 0.01 and absolute log fold change > 1). “Up” refers to upregulated genes and “Down” refers to downregulated genes.
B. Raindrop plot comparing interchromosomal contact frequencies for the control and 24-hour treatment conditions. Error bars: SEM. Data was pooled from two independent biological replicates (N = 2). Significance was calculated by unpaired t test (**< 0.01).
C. Dot plot showing the Gini coefficient as a measure of transcriptional divergence for all three conditions.
