# Supplementary material for: Depletion of lamins B1 and B2 promotes chromatin mobility and induces differential gene expression by a mesoscale-motion-dependent mechanism
Source: Genome Biol. 2024 Mar 22;25:77. doi: 10.1186/s13059-024-03212-y (PMC10958841; doi:10.1186/s13059-024-03212-y)
Supplement: Supplementary file 3 — Additional file 3. Uncropped western blots of H3K27me3 levels before and after 24-h auxin treatment [90]. [file 13059_2024_3212_MOESM3_ESM.pdf]

Uncropped Western Blots

H3K27me3

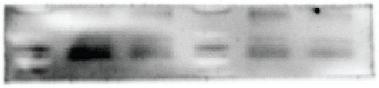

B-actin

H3K27me3

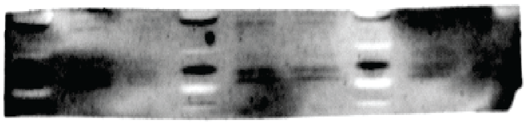

H3K27me3

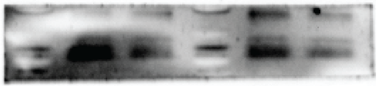

B-actin

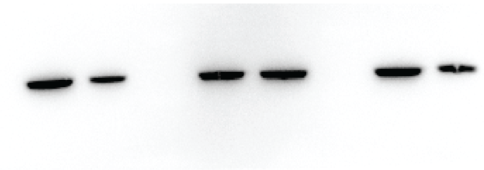

NOTES:

- **Left side:** H3K27me3 blot is 10 second exposure, left lanes were used for analysis (1st replicate)
- **Right side:** Top H3K27me3 blot is 10 second exposure. Bottom H3K27me3 blot is the left two lanes with a 20 second exposure. These two lanes were used for analysis (2nd and 3rd replicates)
