# Supplementary material for: Depletion of lamins B1 and B2 promotes chromatin mobility and induces differential gene expression by a mesoscale-motion-dependent mechanism
Source: Genome Biol. 2024 Mar 22;25:77. doi: 10.1186/s13059-024-03212-y (PMC10958841; doi:10.1186/s13059-024-03212-y)
Supplement: Supplementary file 5 — Additional file 5. Review history. [file 13059_2024_3212_MOESM5_ESM.docx]

**1^st^ round**

**Reviewer 1**

In this manuscript, the authors report effects of acutely degrading lamin B1, B2 or both, on chromatin behavior. They rely on genomics approaches to map LADs (lamin DamID) and 3D interactions (in situ Hi-C), and on fixed- and live-cell imaging techniques to investigate changes in chromatin dynamics resulting from B-type lamin degradation. They report no or minor changes in 3D chromatin interaction properties (TAD # and size, A/B compartmentalization, and contact probabilities in and outside LADs. They report however changes in various aspects of chromatin dynamics by imaging, using among other techniques power wave spectroscopic (PWS) microscopy.
The manuscript is rich in data, but genomic novelty is overall marginal: 1) the authors confirm that B-type lamins only play a minor role, of any, on chromatin organization based on Hi-C and DamID data; 2) their imaging data, although they seem innovative and interesting from a physical point of view, only show marginal effects which are hard to give biological meaning to - perhaps because there is little attempt to do so.
Indeed, the authors fail to relate their findings to the literature, which is rich in this domain. How are their findings consistent with, or discordant from, other reports of the effects of KO or KD of lamins (lamin A/C in particular - there is only one line about this in the paper), LBR, other NE proteins, on chromatin organization? Papers should be discussed and cited. How to the findings relate to inverted nuclei organization? How to they relate to heterochromatin distribution and LAD properties in senescent cells or cancer, when lamin B1 is degraded?
The authors seem to be strong in chromatin imaging, which is the focus of this paper. It would be informative to see more details on the (chromatin) scale of their experiments and how their data relate to other imaging approaches to looks at chromatin organization and dynamics (Fig. 4-5).
The gene expression data are quite striking in the sense that degradation of lamin B1+B2 leads to changes in LADs and inter-LADs (this has been reported) and that auxin wash out restores expression patterns essentially to their pre-auxin treatment state. The analysis however lacks statistical quantifications of the gene expression changes reported. How are gene expression changes outside explained? Some attempts could be perhaps made by relating this to imaging data. See below.
Since the authors also analyze chromatin by imaging nuclear radial aspect (center-periphery), and since functionally, lamins provide this radial functionality to chromatin, it would be interesting analyze gene expression changes in relation to chromatin dynamics / compaction / center-periphery - at least discuss these aspects in the discussion. This would be interesting and novel, as the literature seldom reports relationships between imaging and expression data on a nucleus scale.
Replicates and statistics
The authors report "Experimental data are presented as the mean ± SD of three independent experiments unless otherwise stated" (Methods), but it is unclear where exactly this applies (except for the RNAseq where this is clear). Hi-C? If so how are the data presented and analyzed? The contact maps are for one experiment I assume. DamID?.
Imaging data: what do the single data points represent on the graphs? How many cells/nuclei were examined, in how many experiments (n=3?). In general, clarity should be brought throughout the manuscript on the actual number of experiments, cells/nuclei etc, analyzed. The statistical tests used should be clearly stated; as such, I also doubt whether they appropriate.

Editorial
Even though some sections are well described (the authors should be commended for that, e.g. on PWS microscopy), other more impactful sections are hard to grasp: e.g. it is very hard to grasp anything substantial from the abstract, which is superficially written with vague "buzz words" (mesoscale, positioning (used 5 times over 9 lines), chromatin dynamics - all repeated) lacking substance.
Another example is in the Conclusions (abstract): …degrading lamin B1 and B2 has several consequences…: what does this mean? Then: "…on structural disease (what is a "structural disease"?) … and cancer: there is no evidence or any disease or cancer relations in the paper. In addition to the writing style, these claims are not substantiated. In short, I would suggest to re-write the abstract in a more tangible manner.
On a similar note, I do not understand anything from the last paragraph of the introduction, which un-necessarily again tries to summarize the findings.

Discussion and model: the discussion is largely a reiteration of the results rather than an interpretation of the data. The authors should attempt to relate the various findings from genomics, imaging and RNAseq experiments to provide a functional view of the roles of B-type lamins on chromatin organization and dynamics. Discussion of the data in relation to previously published work is missing.
The model presented in Fig 7 is too detailed / too complicated, making it difficult to grasp the main point.

**Reviewer 2**

In the manuscript titled "Depletion of lamins B1 and B2 alters chromatin mobility and induces differential gene expression by a mesoscale-motion dependent mechanism" by Pujadas et al., the authors generated an auxin-inducible degron system targeting both B1 and B2-type lamins simultaneously to overcome the challenges posed by their essentiality and redundancy. Using Hi-C, they found depletion of B-type lamins has minimal disruption to mesoscale chromatin folding. The author then applied live-cell Dual Partial Wave Spectroscopic (Dual-PWS) microscopy and found that B-type lamin degradation results in overall greater magnitude of chromatin turnover with a slower rate of motion. They finally investigated how gene expression is affected by RNAseq. Overall, this work provides valuable data for understanding the role of B-type lamins in chromatin structure, dynamics and gene expression. However, the quality and sufficiency of some key data are not high enough to support some of the conclusions. Therefore, a major revision is required for the manuscript.

(1) In Figure 3B-C, the authors say that when we compared |s| for chromosomes associated with the periphery (e.g., chromosome 3) to those associated with the nuclear center (e.g., chromosome 19), we observed a greater difference between |s| in chromosome 3. However, I could not see any difference in Contact probability curves either across Non-LAD or across LAD between chromosome 3 and 19. Those |s| values do not seem to be much different.
(2) In Figure 3D, the authors say that analysis of each chromosome demonstrated an inverse relationship between |s| within LADs and the percent coverage of the chromosome confined within LADs. I do not understand why and what is the significance of this observation. And also in Figure 3D, using "Lamin B1 Coverage (%)" to represent LAD may not be proper.
(3) In Figure 3E, are there any statistical significances for those contact scaling by segment?
(4) The following statement/conclusion "Taken together, our temporal analysis indicates that B-type lamin degradation results in overall greater magnitude of chromatin turnover with a slower rate of motion" seems very important. However, it is also quite confusing. The authors are suggested to provide more biophysical pictures to help the readers grasp the insights.
(5) In relation with (4), what is rippling effect?
(6) The image quality and analysis of Figure 5A are not acceptable.
(7) In the section "Auxin treatment results in lamina-dependent changes in 3D spatial localization and dynamic chromatin reorganization", the authors utilized CRISPR-Sirius to label two genes XXYLT1 and TCF3 on Chromosomes 3 and 19, respectively. I have several questions for this part. First, I do not see any "dynamic" chromatin reorganization data although they used live cell genomic loci labeling. The authors also mentioned "To measure the spatial distance and dynamics of the XXYLT1" in the text. Figure 5E-F only show the relative distance, which can be more easily provided by FISH and CRISPR-Sirius is not necessary. Second, I do not think the relative distance of only two gene loci is enough to make conclusion on lamina-dependent changes in 3D spatial localization and dynamic chromatin reorganization. Third, Figure 5E lower right panel image looks weird. Fourth, in the main text, the authors quantified the average distance between the nuclear periphery, but in Figure 5F they show distance from center. It is better off consistent.
(8) For gene expression alteration upon acute B-type lamin depletion, the authors performed RNAseq and analyzed data within both LADs and non-LADs. I have several questions for this part. First, I am not sure for the untreated group whether the authors applied mock transfection, which is crucial for using the untreated group as the control and basal level for examining DEGs. Second, the data of the group "6 day withdraw DEGs" (Figure S6B) have hundreds of Up and Down DEGs that are similar with the 12 hr group. I do not see how the authors make a conclusion that "Further, restoration of B-type lamins after growing HCT116LMN(B1&B2)-AID cells in auxin-free media for 6 days after 48 hours of auxin treatment returned cells to basal levels of gene expression." Third, what is the main mechanism that causes Up and Down DEGs? Gene relocation? Are XXYLT1 and TCF3 up or down DEGs?
(9) Regarding the action mechanism of B-type lamins, the authors claimed that "Our findings suggest that the mechanistic role of B-type lamins is stabilization of heterochromatin and chromosomal positioning along the nuclear periphery" and "mesoscale-motion dependent mechanism". I do not think the data in the current manuscript have provided sufficient evidence to support these claims. They need to do chromosome painting to measure the size and distribution of certain chromosomes and chromatin domains. They also need to provide additional evidence to support the "mesoscale-motion dependent mechanism" by manipulating mesoscale-motion with approaches other than lamin depletion.
(10) Lastly, I feel that the following logic makes more sense to reveal the function of lamins: investigate mesoscale chromatin structure -> Chromosome painting size of chromosome and chromatin domains, distribution of chromosome and heterochromatin -> chromatin and gene dynamics -> gene expression

**Reviewer 3**

In this manuscript, Pujadas and colleagues utilise a chemically inducible degron system in human cells to deplete LAMINB1 and B2. They utilise this acute loss of function system to investigate the impact of LAMINB loss on cell fitness, nuclear organisation, chromatin architecture and gene regulation. The paper is driven by the hypothesis that LAMINB1 and B2, as critical structural components of the nuclear periphery, serve to create a repressive compartment for peripheral associated genes. The authors conclude that loss of LAMINB1 and B2 has little impact of mid-range chromatin folding (compartmentalisation, TADs etc) but leads to pronounced changes in gene expression concomitant with increased chromatin mobility within the nucleus. Interestingly the changes in gene expression seem not to follow the expectations that genes associated with the nuclear periphery are preferentially repressed.

The subject of this study addresses an important intersection between nuclear organisation, chromatin architecture and gene regulation. The system employed for depletion, the quality of the Hi-C data and the gene expression data are of significant interest. However this reviewer does not feel that some of the other assays performed are robust or well enough explained to support some of the conclusions, and thus to allow for publication in as currently presented. Therefore, I believe that the authors must address the following concerns and revise the manuscript accordingly before the manuscript can be accepted for publication in Genome Biology.

**Major Comments:**

*Clarity:*

There are various issues with the clarity of the text and figures, these need to be improved (for specific examples see minor comments below). In addition, some sections should be removed as they are not robust and distract from the main findings of the study (see below).

Fig S4E – The clover signal shown on the X axis should not be plotted as an inverse as it makes the interpretation of the relationship between D and LAMINB much less clear. Indeed page 13 – Line 316 should read strong anti-correlation not correlation.

In several instances, the figure panels are discussed in the wrong order in the text and this should be corrected. For details see below in minor corrections.

*Control experiments:*

The functional normality of tagged lines is assessed based on the nuclear staining shown in Figure 1, however there are other important test that are required also. Do the targeted cells grow as parental e.g. cell cycling, gene expression, genome organisation in the absence of TIR1 indication? Some additional validation is required to address at least some of these concerns, or justification as to how they have been mitigated.

*Incomplete loss of Lamin B1 and 2:*

It is commendable that the authors utilise the clover as a sensitive indicator of degradation, however it does highlight that depletion is not complete compared to controls within the experimental times used (see Figure 1F / S1B / S1D). There is also no explanation of what the two tables are in S1. The labelling appears to suggest they are the same information, so are these quantifications for individual replicates or the values from different tagged lines? Furthermore on Page 8 (line 197/198) it is stated that nearly 80% of LMNB1-mAID was degraded within the first 6 hours of auxin treatment. What data is this based upon? This does not look to be the case in the FACs analysis and if this is based on the immunoblot this should be explicitly stated. Whilst it is likely that many published degron studies have incomplete loss, you have clearly demonstrated it here by comparing the blot to the clover FACS. Consequently you must, at the very least, comment on this and temper your conclusions to include the caveat that there is likely residual LAMINB1 and B2 left and this may limit the extent of the phenotypes assayed here.

*Incomplete or unconvincing data:*

Page 9; 211-214. ‘In addition to the change in nuclear volume, confocal sections in HCT116LMN(B1&B2)-AID cells revealed lamin-specific spherical and discrete puncta, which has been previously demonstrated in the nucleus of cultured cells 36,37 (S2C).’ It is not clear why this is important or of interest. Nor is it indicated in the legend for S2C what conditions this represents - is this auxin treated or not (e.g. if yes are these the non-degraded cells analysed in S2A) or is this Auxin treated without TIR1 induction? Do you see these puncta in WT or parental cells? Unless this is an important point it is a distraction from the main points and should be removed. If it is important this should be justified and discussed.

The concept of changes in chromatin marks is an interesting one, however the data shown is not convincing. Fig 5A and B - How is the WB quantified and are there replicates? IFs are poor quality and the analysis uninformative.

Fig 5E – the data for TCF3 is noisy with high background and does not seem to reflect the quantification in 5F. Fig 5F – indicates distance from centre in the plot whereas the text on page 17 Line 407/408 ‘between the nuclear periphery and either the XXYLT1 or TCF3 gene loci’ indicates the distance from the periphery. The later would not fit with the plot. This needs to be corrected in the text.

Page 18 – paragraph from 438-449. The gene expression analysis is interesting but this section lacks two pieces of key information to assess the prevalence of DEGs in LADs vs non-LADs. Firstly what fraction of the genome are scored as LADs e.g. are there less LAD genes because the fraction of the genome covered by LADs is smaller? On a similar note, LAD gene density is disproportionately low. If you factor this in – is the fraction of LAD genes that are differentially expressed more than the fraction of non-LAD genes that are differentially expressed? Also it would be better to focus the analysis on 12 h as this will be the immediate early effects and more likely to give you a clear direct influence of periphery loss (less secondary expression changes at earlier time points).

Methods:

For CRISPR-syrius (P16 lines 401-404). ‘Specifically, we chose one intronic gene region on Chromosome 3 (XXYLT1) containing ~ 333 tandem repeats and one intronic gene region on Chromosome 19 (TCF3) containing ~ 36 tandem repeats as a reference to the nuclear interior’. It is unclear where the repeats for the two studied genes come from. Are these endogenous repeats within the genes targeted by CRISPR or are these engineered into the cells? Whilst this is a very important question in the paper, the data (particularly the examples) are poor and do not represent the observed trend that is being discussed. Whilst this is a nice approach, it is unclear why a conventional approach e.g. DNA FISH was not employed here.

Page 15-16 lines 379-382. This conclusion is interesting but is not supported by robust data. This needs to be clarified or supported by additional data.

**Minor Comments / corrections:**

Page 7; line 168 Fig 1D referred to before 1C – re-order figure or text accordingly.

Page 7; line 177-178 – ‘Genomic localization has previously demonstrated utility as an indicator of functional retention in transcription factor binding’. It is unclear what this means – please rephrase.

Figure 1C. The full genotype should be shown above the panel e.g. LMN(B1&B2)-AID as the shorthand is confusing as it resembles the naming of the staining/label. The same is also noted in figure S1A, and although the figure is transposed, cell line vs the staining/tag should be made clearer.

Figure 1G, S2A-B – the effect size is small, how does this compare to WT or parental cell controls?

Fig 2A. It would be useful to see some additional zoomed in shots of the Hi-C plots as the whole genome views are not informative at this scale. It would be interesting in particular, to see regions associated with LADs and non-LADs as a reference for later sections.

Fig 2B – need to label A and B compartments on the plots.

Figure 2D. There is a subtle reduction in size and increase in number of TADs. This presumably means that some TADs called as one large TAD in control cell Hi-C become fragmented and identified as separate sub-TADs following LAMINB loss. Is this the case and is there any distinction between where these TADs are relative to LADs?

Page 10 lines 247/248. Fic 2C is referenced after 2D in the text

Page 11 lines 260-265. Fig 3E is referred to before 3B-D.

Fig 3E. Has statistical analysis been performed on this? What is the result?

Fig4 – The y axis label of D looks like panel labels – needs to tidy up the figure for clarity.

Page 13 – lines 324-330. This section needs to be clarified to make it easier to follow to a non-specialist what the underlying biology you are looking at is here.

Page 14 – lines 340-342. ‘Taken together, our temporal analysis indicates that B-type lamin degradation results in overall greater magnitude of chromatin turnover with a slower rate of motion’. What does this actually mean for chromatin in the nucleus; turnover of what?

Fig 5D – I think the y label should read foci not loci.

**Authors’ response**

Reviewer #1:

**Remarks to the Author:** In this manuscript, the authors report effects of acutely degrading lamin B1, B2 or both, on chromatin behavior. They rely on genomics approaches to map LADs (lamin DamID) and 3D interactions (in situ Hi-C), and on fixed- and live-cell imaging techniques to investigate changes in chromatin dynamics resulting from B-type lamin degradation. They report no or minor changes in 3D chromatin interaction properties (TAD # and size, A/B compartmentalization, and contact probabilities in and outside LADs.  They report however changes in various aspects of chromatin dynamics by imaging, using among other techniques power wave spectroscopic (PWS) microscopy.

The manuscript is rich in data, but genomic novelty is overall marginal: 1) the authors confirm that B-type lamins only play a minor role, of any, on chromatin organization based on Hi-C and DamID data; 2) their imaging data, although they seem innovative and interesting from a physical point of view, only show marginal effects which are hard to give biological meaning to - perhaps because there is little attempt to do so.

Indeed, the authors fail to relate their findings to the literature, which is rich in this domain. How are their findings consistent with, or discordant from, other reports of the effects of KO or KD of lamins (lamin A/C in particular - there is only one line about this in the paper), LBR, other NE proteins, on chromatin organization? Papers should be discussed and cited. How do the findings relate to inverted nuclei organization? How to they relate to heterochromatin distribution and LAD properties in senescent cells or cancer, when lamin B1 is degraded?

**Response:** We would like to thank the reviewer for their perspectives, positive feedback, and detailed examination of our manuscript. We have carefully revised the manuscript to better relate our findings to the literature (i.e., LBR, NE proteins, Lamin A/C) in the discussion section. We have added more citations and potential explanations in the discussion section to provide more biological meaning to the imaging data. We have also provided a more detailed explanation of the proposed mechanism throughout the main text and in the discussion section. Our point-wise responses to the reviewer’s comments are detailed below.

Although this manuscript reports minor changes in 3D chromatin interaction properties (TAD # and size, A/B compartmentalization, and contact probabilities in and outside LADs), LADs may be redistributed with LMNB1/B2 depletion. We propose that changes in nuclear morphology caused by LMNB degradation alter heterochromatin distribution and LAD partitioning, which among other possible mechanisms, induces differential gene expression both within and outside of LADs.

In addition, this manuscript used the Auxin-inducible Degron System, which is different from KO or KD, to acutely deplete nuclear lamins. Other reports of the effects of KO or KD of lamins are kinds of permanent depletion which may cause big changes in 3D chromatin interaction properties. However, it is possible the acute depletion mainly causes LAD changes. Further, these studies have solely relied on single degradation of Lamin B1 or Lamin B2, whereas in this study, we have assessed both the individual and compound degradation of both B-type lamins.

**Point 1:** The authors seem to be strong in chromatin imaging, which is the focus of this paper. It would be informative to see more details on the (chromatin) scale of their experiments and how their data relate to other imaging approaches to looks at chromatin organization and dynamics (Fig. 4-5).

**Response:** We would like to clarify that the data presented in this manuscript at the chromatin scale span across several length scales. DNA (~1 nm) and histones (~10 nm) are well below the diffraction limit of light (~200 nm) and therefore require labeling with fluorescent proteins or other large probes.

Figure 6 contains dynamic PWS data that allows us to study the statistical chromatin conformation across thousands of live cells. In comparison to other chromatin imaging techniques, PWS allows for label-free measurements to extract not only structural information, but temporal information within the same acquisition. Measurements like fractional moving mass provide a useful way to assess chromatin mobility when averaged across the nuclear area. Unlike other methods, this technique allows us to study the mobilization of chromatin domains without the use of fluorescent tags or the need to fix cells. Although this technique is high-throughput and allows for population-based measurements, however, these measurements are not molecularly specific. Therefore, we applied super-resolution imaging techniques (i.e., STORM) in **Figure 7** to both enhance spatial resolution and obtain more biologically relevant information below the diffraction limit. While this technique allows for multiplexing capabilities to detect several histone modifications, for example, it is not a high-throughput technique and requires cellular fixation.

**Figure 7** also contains immunofluorescence data, which is high-throughput and can be applied to live or fixed cells but does not reveal information at length scales below the diffraction limit. However, it can be used in combination with chromosome-specific approaches such as chromosome painting **(Figure 5)** or gene-specific labeling approaches such as CRISPR-Sirius **(Figure 7)** or FISH **(Figure 8)**. Visualization of time-dependent chromatin changes in individual cells remains a challenge in the field. A large drawback of chromosome painting and FISH is that these techniques require the use of formamide, which severely damages chromatin structure. Therefore, we applied CRISPR-Sirius to measure the distances of chromosome-specific loci relative to the nuclear center in live cells. CRISPR systems for site-specific fluorescent labeling of genomic loci provide a targeted method for visualizing chromatin dynamics in live cells, but still include several limitations such as rapid photobleaching of the fluorophores, the requirement of endogenous tandem repeats, and high background fluorescence.

To study changes in chromatin organization and dynamics upon B-type lamin perturbation, other groups have utilized chromosome painting and CRISPR-SunTag (Chang et al., 2022; Protein Cell), immunostaining with FISH and polymer simulations (Falk et al., 2018; Nature), or have primarily relied on sequencing-based techniques such as Hi-C or DamID-derived imaging approaches to visualize LADs in fixed cells (Guelen et al., 2008; Nature and Kind et al., 2013; Cell).

Importantly, all these studies have been completed in different cell types and have typically focused on only lamin B1 or lamin A perturbation. Further, their results may differ from those in our manuscript based on the nature of the specific techniques used, the type of perturbation, cell type, among other factors.

**Point 2:** The gene expression data are quite striking in the sense that degradation of lamin B1+B2 leads to changes in LADs and inter-LADs (this has been reported) and that auxin wash out restores expression patterns essentially to their pre-auxin treatment state. The analysis however lacks statistical quantifications of the gene expression changes reported. How are gene expression changes outside explained? Some attempts could be perhaps made by relating this to imaging data. See below.

**Response:** We would like to thank the reviewer for their feedback and have added additional experiments and analyses to explain how differential gene expression occurs outside of LADs.

To address the reviewer’s concerns, we have supplied for RNA-sequencing analysis to determine how many DEGs are found within and outside of LADs, along with their direction of change (up vs. downregulated). **Please refer to Figures 8B and S7A-D. Please refer to lines** **553-563.**

Using the same publicly available Lamin B1 DamID data from HCT116 cells, we again segmented the genome into regions within LADs and regions outside LADs. After 48 hours of lamin depletion, we identified nearly 22% of enriched genes within LADs (adjusted P value < 0.01 and absolute log fold change > 1) **(Fig 8B; Fig. S7A-B)**. Anomalously, lamin B1 and lamin B2 depletion predominantly generated the highest number DEGs outside of LAD boundaries for all three timepoints, with the greatest number at 48 hours of auxin treatment (374 genes within LADs, 1331 genes outside of LADs at 48 hours) **(Fig. S7A-B)**. Further, the log fold change of DEGs outside of LADs was larger in magnitude than within LADs, indicating that they were disproportionally influenced by loss of B-type lamins **(Fig. S7C)**. Unexpectedly, in LAD regions, there was no clear trend towards upregulation or downregulation in gene expression. In non-LAD regions however, there appeared to be more upregulated genes **(Fig. 8B; Fig. S7D)**.

Additionally, we have since applied FISH to label genomic loci that were differentially expressed in fixed cells. The genes that were upregulated (Up DEGs) used in these FISH experiments are *CEM1P* (Chr15:80,779,370-80,951,771) and *SLC03A1* (Chr15:91,853,708-92,172,435). Both genes are within LADs based on the DamID data. We also performed FISH with a gene that was downregulated (Down DEG) known as *CYP1A1* (Chr15:74,719,542-74,725,528). This gene is located outside of a LAD based on the DamID data. We have also added STORM experiments to further verify that heterochromatin shifts from the nuclear periphery to the nuclear interior upon B-type lamin degradation. **Please refer to Figures S9A-B.** **Please refer to lines 583-594.**

We found that the relative distance between *SLC03A1* and *CEM1P* within nearby LADs did not change, but the distance between *SLC03A1* and *CYP1A1* outside of a LAD decreased **(Fig. 8C; Fig. 8E)**.  We interpret this mobility of gene loci as LADs disassociating from the nuclear periphery and moving into the interior nuclear space as cohesive units. This cohesive internal shift of gene loci is in line with the reduction of H3K9me3 intensity at the nuclear periphery demonstrated in SMLM experiments. Based on these results, LAD redistribution may be one of the mechanisms that causes differential gene expression after lamin degradation, although genes within LADs were not entirely upregulated **(Fig. S8A)**.  Our data support a model in which B-type lamin degradation does not significantly alter genome topology, but induces significantly increased chromatin mobility (e.g., re-localization of peripherally associated chromosomes, gene loci, and heterochromatin) at the nuclear scale to facilitate differential gene expression within and around LADs **(Fig. 8D)**.

**Point 3:** Since the authors also analyze chromatin by imaging nuclear radial aspect (center-periphery), and since functionally, lamins provide this radial functionality to chromatin, it would be interesting analyze gene expression changes in relation to chromatin dynamics / compaction / center-periphery - at least discuss these aspects in the discussion. This would be interesting and novel, as the literature seldom reports relationships between imaging and expression data on a nucleus scale.

**Response:** To address the reviewer’s comment, we have attempted to better relate measurements from confocal imaging (e.g., the coefficient of variation) to the proposed mechanism of heterochromatin-associated chromatin redistribution promoting differential gene expression. **Please refer to lines 462-470.**

To assess if the observed changes in chromatin dynamics could be further explained by chromatin decompaction, we used spinning disk confocal microscopy to measure the CoV in DAPI-stained HCT116^LMN(B1&B2)-AID^ cells. Previously used to assess the degree of heterogeneity of DNA signal across the nucleus, the CoV is calculated as the standard deviation of the DAPI intensity values divided by the mean value of nuclear pixel intensity for each nucleus. Our results demonstrated that upon auxin treatment, chromatin compaction slightly decreased in HCT116^LMNB1-AID^ (1.11%), HCT116^LMNB2-AID^ (2.31%), and HCT116^LMN(B1&B2)-AID^ (2.86%) cells **(Fig. 7C, S5F-G)**. Therefore, the removal of B-type lamins results in destabilization, re-localization, and a global reduction of heterochromatin that is in part mediated by chromatin decompaction.

We have re-analyzed the bulk RNA-sequencing data based on the reviewer’s suggestions. Specifically, we compared specific pairs of chromosomes and determined how their gene expression changed in relation to being either within or outside of LADs. **Please refer to Figures S9A-B.** **Please refer to lines 602-609.**

To further explore how gene expression changes relate to the observed changes in chromatin dynamics, we examined the DEGs after 48 hours of auxin treatment that were specific to chromosomes tested in chromosome paint and FISH experiments. Chromosome pairs 1 & 18, 2 &19, and 15 & 22 all had more DEGs outside of LADs that were also more upregulated than downregulated **(Fig. S9A)**. We also found that the interchromosomal contact density between all pairs tested was reduced both within and outside of LADs, with specific chromosome pairs exhibiting more significant changes than others (i.e., pairs 6 & 15 and 5 & 14 both within LADs) **(Fig. S9B)**.

We have also updated the discussion to better integrate the conclusions from all the data presented in the manuscript. **Please refer to lines 641-664.**

B-type lamins regulate spatial chromatin organization as we observed that their degradation leads to the movement of gene-specific loci towards the nuclear interior and increased density fluctuations in the chromatin polymer. This could possibly be explained by a transition from relatively large, anchored LADs to smaller, but still substantial domains enriched in heterochromatin. Chromatin packing scaling may increase due to a larger volume fraction of domains upon B-type lamin disruption. This may be verified by electron microscopy, which has previously been used to indicate that the mobility of peripheral LADs is largely confined to the layer of densely staining heterochromatin.  Conversely, it has been proposed that LADs may not play a direct gene silencing, but rather that the association of genomic loci with the nuclear lamina may reinforce transcriptional states. This could help explain why DEGs within LADs were not primarily upregulated when LADs shift away from the nuclear lamina, but those outside of LAD boundaries were. However, recently proposed heteropolymer models such as the Minimal Chromatin Model (MiChroM) and ChIP-seq assays across several human cell types are in line with our experimental data and therefore supports the explanation of chromatin dynamics in establishing higher-order chromatin organization and differential gene expression. Chromatin dynamics may change because in the presence of B-type lamins, LADs are attached to the nuclear periphery and most of the captured motion by live-cell dual-PWS is due to the non-LAD polymeric structures, which are small and therefore have a larger diffusion coefficient. When B-type lamins are degraded, however, the new non-attached LADs begin to shift away which leads to a substantial increase in the fractional moving mass. As demonstrated by measuring TAD sizes that were associated with LADs from the DamID data, these domains are larger and physically move more slowly throughout the nuclear area which leads to a lower diffusion coefficient.  Therefore, our dual-PWS measurements are consistent with those obtained by SMLM imaging, which demonstrated the physical breakdown of H3K9me3 components at the nuclear periphery.

**Replicates and statistics:**

**Point 4:** The authors report "Experimental data are presented as the mean ± SD of three independent experiments unless otherwise stated" (Methods), but it is unclear where exactly this applies (except for the RNAseq where this is clear). Hi-C? If so how are the data presented and analyzed? The contact maps are for one experiment I assume. DamID?. …

**Response:** We would like to clarify that the type of statistical analysis used, cell numbers, and number of experiments have been updated in the figure panels and methods. Hi-C contact maps are from pooled replicates for each condition. Then TADs, contact scaling, etc., are called from the summed pool. Statistical tests are differences in the observed frequencies. Using these pooled replicates, we calculated *s* for each individual chromosome which gives 22 points.

**Please refer to lines 250-252.**

For each cell type, we generated more than a 1 billion contacts from cells before (~ 1,354,831,021) and after 24 hours of auxin treatment (~ 1,210,053,449). At least two biological Hi-C replicates for all three cell lines were obtained.

For DamID, the merged replicates are publicly available online, specifically at: <https://data.4dnucleome.org/experiment-set-replicates/4DNES24XA7U8/>. This has also been specified in the Availability of Data and Materials section. We have cited the 4D Nucleome data repository accordingly.

**Please refer to lines 1212-1214.**

The publicly available hg19 DamID track was downloaded from the 4D Nucleome data repository (data.4dnucleome.org). The tracks are obtained from two independent biological replicates.

**Please refer to lines 1239-1241.**

DamID data obtained from two independent biological replicates (raw and processed files) are available at the 4D Nucleome data repository (<https://data.4dnucleome.org/files-processed/4DNFICCV71TZ/#file-overview>).

**Point 5:** Imaging data: what do the single data points represent on the graphs?  How many cells/nuclei were examined, in how many experiments (n=3?).  In general, clarity should be brought throughout the manuscript on the actual number of experiments, cells/nuclei etc, analyzed. The statistical tests used should be clearly stated; as such, I also doubt whether they appropriate.

**Response:** We would like to clarify that the single data points on the graphs represent individual nuclei, compiled over three separate experiments unless otherwise specified in the figure captions. We have since modified the main text and figure captions to clearly state the number of experiments, number of cells, and the statistical tests used.

We appreciate the reviewer bringing up the question about statistical testing, and whether other types of analyses may be superior to the subset of individual t-tests we performed. During initial preparation of the manuscript, we spent significant time debating the most appropriate – and biologically meaningful – approach to take to testing, ultimately settling on the tests that we included. That being said, the comment from the reviewer led to us reviewing the testing we had performed in the past, and performing some additional tests to determine whether other approaches might be superior. In summary, we believe the testing we performed focuses in on the most biologically relevant information for our work, while still fairly representing what is and is not statistically significant. We found that the conclusions remained the same with other tests, and if not, changed the type of test and significance accordingly.

We have modified the Quantification and Statistical Analysis section in the methods accordingly. **Please refer to lines 12-18-1227.**

Statistical analysis was performed using GraphPad Prism 10.1.1 and Microsoft Excel.  Pairwise comparisons were calculated on datasets consisting of, at a minimum, biologically independent duplicate samples using two-tailed unpaired t test. Experimental data are presented as the mean ± SEM unless otherwise stated in figure legends. The type of statistical test is specified in each case. Significance was calculated by Mann-Whitney test for measuring distances of foci in CRISPR-Sirius and FISH experiments. Multiple comparison corrections were applied for datasets with comparing more than two groups and multiple comparisons. A *P* value of < 0.05 was considered significant. Statistical significance levels are denoted as follows: n.s. = not significant; **P*<0.05; ***P*<0.01; ****P*<0.001; *****P*<0.0001. Sample numbers (# of nuclei, n), the number of replicates (N), and they type of statistical test used is indicated in figure legends.

**Editorial:**

**Point 6:** Even though some sections are well described (the authors should be commended for that, e.g. on PWS microscopy), other more impactful sections are hard to grasp: e.g. it is very hard to grasp anything substantial from the abstract, which is superficially written with vague "buzz words" (mesoscale, positioning (used 5 times over 9 lines), chromatin dynamics - all repeated) lacking substance.

**Response:** We would like to thank the reviewer for their insight and positive feedback on sections such as the explanation of PWS microscopy. We have revised the abstract to remove vague “buzz words” mentioned by the reviewer. Specifically, we have revised the results section of the abstract. **Please refer to lines 59-69.**

Using live-cell Dual Partial Wave Spectroscopic (Dual-PWS) microscopy, Stochastic Optical Reconstruction Microscopy (STORM), *in situ* Hi-C, CRISPR-Sirius, and Fluorescence *in situ* hybridization (FISH), we demonstrate that lamin B1 and lamin B2 are critical structural components of the nuclear periphery that create a repressive compartment for peripheral-associated genes. Although lamin B1 and lamin B2 depletion minimally alters higher-order chromatin folding, it disrupts cell morphology, significantly increases chromatin mobility, redistributes both constitutive and facultative heterochromatin, and induces differential gene expression both within and near LAD boundaries. Critically, we demonstrate that chromatin territories expand as upregulated genes within LADs radially shift inwards. Our results indicate that the mechanism of action of B-type lamins is derived from their role in constraining chromatin motion and spatial positioning of gene-specific loci, heterochromatin, and chromatin domains.

**Point 7:** Another example is in the Conclusions (abstract): …degrading lamin B1 and B2 has several consequences…: what does this mean? Then: "…on structural disease (what is a "structural disease"?) … and cancer: there is no evidence or any disease or cancer relations in the paper.  In addition to the writing style, these claims are not substantiated. In short, I would suggest to re-write the abstract in a more tangible manner.

**Response:** We have revised this section to be more tangible, as suggested by the reviewer. **Please refer to lines 72-76.**

Our findings suggest while B-type lamin degradation does not significantly change genome topology, it has major implications on 3D chromatin conformation at the single cell level both at the lamina-associated periphery and the non-LAD-associated nuclear interior with concomitant genome-wide transcriptional changes. This work raises intriguing questions about the individual and overlapping roles of lamin B1 and lamin B2 in cellular function and disease.

**Point 8:** On a similar note, I do not understand anything from the last paragraph of the introduction, which un-necessarily again tries to summarize the findings.

**Response:** We have revised this section to enhance clarity. **Please refer to lines 144-151.**

Surprisingly, although we observed an increase in chromatin density fluctuations, heterochromatic translocation, and gene loci internalization, mesoscale chromatin folding was minimally altered. Our findings suggest that substantial upregulation and downregulation of genes both within and outside of LADs is a direct consequence of these chromatin dynamics. By integrating chromatin imaging and gene expression data across several length scales, we propose that B-type lamins synergistically contribute to chromatin conformation (i.e., domains defined spatially at the single-cell level) independent of nuclear-scale chromatin regulation (i.e., contact-based domains in a cell ensemble).

**Point 9:** Discussion and model: the discussion is largely a reiteration of the results rather than an interpretation of the data. The authors should attempt to relate the various findings from genomics, imaging and RNAseq experiments to provide a functional view of the roles of B-type lamins on chromatin organization and dynamics. Discussion of the data in relation to previously published work is missing.

**Response:** We have modified part of the discussion to better relate our findings and provide a more functional view of the roles of B-type lamins on chromatin organization and dynamics. We have specifically referenced relevant findings in previously published work, such as the effects of KO or KD of lamins (lamin A/C in particular), LBR, and other NE proteins on chromatin organization. We have also attempted to relate our findings to inverted nuclei, cellular senescence, and cancer cell chromatin organization when B-type lamins are degraded. **Please refer to lines 666-701.**

**Point 10:** The model presented in Fig 7 is too detailed / too complicated, making it difficult to grasp the main point.

**Response:** We would like to clarify that this figure has since been modified to be significantly less detailed. **Please refer to Figure 8F and see below.**

Reviewer #2:

**Remarks to the Author:** In the manuscript titled "Depletion of lamins B1 and B2 alters chromatin mobility and induces differential gene expression by a mesoscale-motion dependent mechanism" by Pujadas et al., the authors generated an auxin-inducible degron system targeting both B1 and B2-type lamins simultaneously to overcome the challenges posed by their essentiality and redundancy. Using Hi-C, they found depletion of B-type lamins has minimal disruption to mesoscale chromatin folding. The author then applied live-cell Dual Partial Wave Spectroscopic (Dual-PWS) microscopy and found that B-type lamin degradation results in overall greater magnitude of chromatin turnover with a slower rate of motion. They finally investigated how gene expression is affected by RNAseq. Overall, this work provides valuable data for understanding the role of B-type lamins in chromatin structure, dynamics and gene expression. However, the quality and sufficiency of some key data are not high enough to support some of the conclusions. Therefore, a major revision is required for the manuscript.

**Response:** We would like to thank the reviewer for their detailed examination of our manuscript and helpful comments. We believe that we have addressed all the reviewer’s concerns and have revised the manuscript accordingly. We hope that this reviewer sees that we have greatly appreciated their feedback and have greatly revised the manuscript.

**Point 1:** In Figure 3B-C, the authors say that when we compared |s| for chromosomes associated with the periphery (e.g., chromosome 3) to those associated with the nuclear center (e.g., chromosome 19), we observed a greater difference between |s| in chromosome 3. However, I could not see any difference in Contact probability curves either across Non-LAD or across LAD between chromosome 3 and 19. Those |s| values do not seem to be much different.

**Response:** We would like to clarify that we indeed did not observe a substantial change in contact probability scaling between the control and 24-hour auxin conditions. However, we did see small chromosome-specific differences in the scaling between LAD and non-LAD segments for same chromosome. This is demonstrated in the left plots (untreated) for both Chromosome 3 and Chromosome 19 in **Figure 4A**. Considering that the contact probably scaling is plotted as an exponential, these plots show a 33.18% increase compared to a 19.06% increase between LADs and non-LADs in Chromosome 3 and Chromosome 19, respectively. Therefore, while loss of B-type lamins does not result in substantial differences in scaling, we demonstrate a clear difference between LAD and non-LAD scaling in a chromosome-specific manner. We have made this clearer in the text. **Please refer to lines 290-296.**

Consistent with this hypothesis, in the control conditions, we observed a lower *|s|* in both a chromosome associated with the periphery (chromosome 3; *|s|* 0.862 vs 1.148 for LAD vs non-LAD) and in the center (chromosome 19, *|s|* 1.002 vs 1.193 for LAD vs non-LAD) in the LAD segments compared to the non-LAD control **(Fig. 4A).** However, despite these differences at baseline, there was no substantial change in contact probability scaling between the control and 24-hour auxin conditions either within LAD domains nor within non-LAD domains for either the peripherally associated or centrally associated chromosome.

**Point 2:** In Figure 3D, the authors say that analysis of each chromosome demonstrated an inverse relationship between |s| within LADs and the percent coverage of the chromosome confined within LADs. I do not understand why and what is the significance of this observation. And also in Figure 3D, using "Lamin B1 Coverage (%)" to represent LAD may not be proper.

**Response:** We would like to clarify that this observation is critical to understand the rate of decay of contacts over genomic distance within versus outside of a LAD. Our collaborators in the Lieberman-Aiden lab, among other groups, have observed contact probability as a function of genomic distance averaged across the genome and that it exhibits a power law scaling between 500 kb and 7 Mb with a slope of -1.08 (Lieberman-Aiden et al., 2009; Science Advances). Within a LAD, a transcriptionally repressive and spatially constrained area, there are presumably many sites of chromosome contacts within a relatively short genomic distance. Based on this relationship, within a LAD, there would be a steeper slope (i.e., -1.5), as there are a greater number of contacts for a shorter distance.

In **Figure 4B**, we demonstrate that the higher the percentage of lamin B1 coverage (association) on a chromosome, the lower the contact probability scaling (*|s|*) is. In other words, more lamin B1 % coverage would result in more contacts scaled over a short genomic distance (i.e., within a constrained LAD environment). Conversely, there was no relationship between |*s*| within non-LADs and the chromosomal coverage of lamin B1 in **Figure S2F**.

As mentioned in our recent publication (Li et al., 2021; Scientific Advances), a chromatin polymer with a higher *D* and, consequently, a lower rate of decrease of chromatin volume concentration as a function of distance from the center of a domain should be associated with a higher contact frequency among distant loci. This would translate into a lower contact probability scaling *|s|.*

Based on this relationship, *|s|* within a LAD should be lower than that of a non-LAD, which is verified in **Figure 4C**.

To avoid confusion, we have since changed the text to describe lamin B1 coverage rather than LAD coverage. Please refer to Figures 4B-C and S2F. **Please refer to lines 298-308.**

Further, analysis of each chromosome demonstrated an inverse relationship between *|s|* within LADs and the percent coverage of the chromosome in contact with lamin B1 **(Fig. 4B)**. However, despite the large difference in *|s|* at baseline **(Fig. 4A)** and inverse relationship between *|s|* and lamin B1 coverage, only minor changes in *|s|* were observed upon B-type lamin depletion with a larger magnitude of effect observed within the non-LAD portions **(Fig. 4C)**. As expected, we observed a lower *|s|* (calculated between 10^5^-10^6^ bp) within LADs compared to non-LADs in untreated cells **(Fig. 4C)**. This is because a chromatin polymer with a slow rate of decay of chromatin volume concentration as a function of distance from the center of a domain (i.e., chromatin within a LAD) should be associated with a higher contact frequency among distant loci. This translates into a lower contact probability scaling *|s|.* As expected, there was no relationship between *|s|* within non-LADs and the chromosomal coverage of lamin B1 **(Fig. S2F).**

**Point 3:** In Figure 3E, are there any statistical significances for those contact scaling by segment?

**Response:** We would like to clarify that we did a paired t-test to determine significance. It has since been added to both the figure panel and figure legend. **Please refer to Figure 3D.**

**Point 4:** The following statement/conclusion "Taken together, our temporal analysis indicates that B-type lamin degradation results in overall greater magnitude of chromatin turnover with a slower rate of motion" seems very important. However, it is also quite confusing. The authors are suggested to provide more biophysical pictures to help the readers grasp the insights.

**Response:** We have since altered the text to rephrase and avoid confusion. We hope this modification has provided more biophysical pictures to help the readers grasp the insights within the discussion section. **Please refer to lines 417-423.**

Taken together, our temporal analysis indicates that B-type lamin degradation results in overall greater magnitude of chromatin chain fluctuations with a slower rate of motion. Such a phenotype would be observed with the dissociation of large structures from a confined state in a crowded environment. Although Dual-PWS is not a molecularly specific technique, this result could be explained by the weaking of chromatin and nuclear interactions at the nuclear periphery. These findings therefore provide further evidence that increased chromatin mobility due to a loss of B-type lamins at the periphery induces conformational changes, even in the nuclear interior.

**Point 5:** In relation with (4), what is rippling effect?

**Response:** We would like to clarify that by “rippling effect” we were referring to chromatin mobility that ranged from the nuclear periphery towards the nuclear interior. We have since removed this wording to enhance clarity.

**Point 6:** The image quality and analysis of Figure 5A are not acceptable.

**Response:** We have replaced the images and analysis in this figure to demonstrate a minor shift more clearly in heterochromatin from the nuclear periphery to the nuclear interior, as well as a shift in euchromatin from the nuclear interior towards the nuclear periphery. This figure has now been replaced with confocal images and an updated regional analysis using CellProfiler to measure the intensity distribution of H3K9me3 and H3K27me3 before and after the addition of auxin. The radial positioning of these marks was determined as a function of the signal intensity measured in each of the ten concentric rings per nucleus. **Please refer to Figure 7A and S5D.**

**Point 7:** In the section "Auxin treatment results in lamina-dependent changes in 3D spatial localization and dynamic chromatin reorganization", the authors utilized CRISPR-Sirius to label two genes XXYLT1 and TCF3 on Chromosomes 3 and 19, respectively. I have several questions for this part. First, I do not see any "dynamic" chromatin reorganization data although they used live cell genomic loci labeling. The authors also mentioned "To measure the spatial distance and dynamics of the XXYLT1" in the text. Figure 5E-F only show the relative distance, which can be more easily provided by FISH and CRISPR-Sirius is not necessary. Second, I do not think the relative distance of only two gene loci is enough to make conclusion on lamina-dependent changes in 3D spatial localization and dynamic chromatin reorganization. Third, Figure 5E lower right panel image looks weird. Fourth, in the main text, the authors quantified the average distance between the nuclear periphery, but in Figure 5F they show distance from center. It is better off consistent.

**Response:** We would like to thank the reviewer for their feedback. We believe that the techniques used in this manuscript such as chromosome painting, Dual-PWS, STORM, and CRISPR-Sirius, and FISH all provide sufficient evidence of dynamic chromatin organization upon B-type lamin reduction. With the addition of chromosome painting, we have demonstrated that although chromosome area does not change upon lamin depletion, there is a significant shift of chromosome 1 from the nuclear periphery towards the nuclear interior. Using both CRISPR-Sirius in live cells and FISH in fixed cells, we have now demonstrated four different gene loci that move internally 24 hours after the addition of auxin.

We chose to initially include CRISPR-Sirius rather than FISH because this technique enables gene-specific localization without the use of reagents that alter sub-200 nm (sub-Megabase pair) scale chromatin structure, such as formamide. CRISPR-Sirius, however, does not. Although we agree with this reviewer that an ideal experiment with live cell genomic loci labeling would be to track these gene loci right after auxin removal, tracking loci over the course of 24 hours to deplete B-type lamins would result in significant photobleaching of both the foci and the Janelia Fluor 646 HaloTag Ligand. Further, we cannot track the position of these foci in the same cells to determine how the position changes during auxin treatment due to the resolution limit of PWS. Using Dual-PWS in live cells, however, we have demonstrated a clear shift not only in chromatin packing scaling from the nuclear periphery towards the center but also, significant regional shifts in fractional moving mass and diffusive motion towards the nuclear interior. Finally, with confocal microscopy and STORM, we have demonstrated that there is a regional shift in heterochromatin towards the nuclear interior and a significant reduction of H3K9me3 at the nuclear periphery. Collectively, we believe that these results confirm lamina-dependent changes in 3D spatial localization and dynamic chromatin reorganization.

**Please refer to Figures 7D-E for the STORM figures and analysis. Please refer to lines 472-483.**

To confirm the reduction of heterochromatin-associated domains specifically at the nuclear periphery, we utilized single-molecule localization microscopy (SMLM) to measure the intensity of H3K9me3 in HCT116^LMN(B1&B2)-AID^ cells **(Fig. 7D)**. This powerful imaging technique dramatically improves spatial resolution in comparison to standard, diffraction-limited techniques and provides molecular-specific information. By utilizing STORM, we quantified H3K9me3 intensity normalized to the total nuclear intensity. As LADs are typically enriched in H3K9me3, we selected this marker to represent LAD distribution specifically at the nuclear periphery. We first partitioned each reconstructed image into two regions spanning the nuclear periphery and interior **(Fig. S6A)** to quantify the Normalized STORM Intensity (NSI) as a reporter for the proportion of H3K9me3 signal within this region relative to the entire nucleus. We found that upon 24 hours of auxin treatment, H3K9me3 NSI was significantly reduced at the nuclear periphery by 25% in comparison a 0.9% increase in NSI at the nuclear interior **(Fig. 7D-E)**.

**Please refer to Figures 8C-E for the FISH experiments and analysis. Please refer to lines 574-588.**

To understand the potential mechanism behind differential gene expression upon B-type lamin degradation, we utilized Fluorescence *in situ* Hybridization (FISH) to label genomic loci that were differentially expressed. Specifically, we identified *SLC03A1* and *CEM1P*, both genes on chromosome 15 that were upregulated, within a few megabase pairs apart, and within LADs based on the public DamID data. We also performed FISH with *CYP1A1*, a gene on the same chromosome that was downregulated and located outside of a LAD based on the DamID data.  First, we visualized pairs of each locus and confirmed that the two genes within LADs shifted towards the nuclear interior upon auxin treatment, consistent with *XXYLT1* in the CRISPR-Sirius experiments **(Fig. 8C-D)**. We then measured their relative distances between each other before and after B-type lamin degradation. We found that the relative distance between *SLC03A1* and *CEM1P* within nearby LADs did not change, but the distance between *SLC03A1* and *CYP1A1* outside of a LAD decreased **(Fig. 8C; Fig. 8E)**.  We interpret this mobility of gene loci as LADs disassociating from the nuclear periphery and moving into the interior nuclear space as cohesive units. This cohesive internal shift of gene loci is in line with the reduction of H3K9me3 intensity at the nuclear periphery demonstrated in SMLM experiments.

We have since replaced the CRISPR-Sirius figure to more clearly show the TCF3 foci. We have also altered the text for consistency when describing the relative distances of these foci for the CRISPR-Sirius experiments. **Please refer to Figure 7F. Please refer to lines 513-530.**

To assess the effect of B-type lamin degradation on chromosome-specific loci in live HCT116^LMN(B1&B2)-AID^ cells, we labeled genomic regions on chromosomes 3 and 19 using CRISPR-Sirius^55^. Chromosomes 3 and 19 were chosen to represent chromosomes that are localized relatively near the nuclear periphery and the nuclear interior, respectively.^55-60^ Specifically, we chose one intronic gene region on Chromosome 3 (*XXYLT1*) containing ~ 333 endogenous tandem repeats, and one intronic gene region on Chromosome 19 (*TCF3*) containing ~ 36 endogenous tandem repeats as a reference to the nuclear interior. To measure the spatial distance and dynamics of the *XXYLT1* gene loci, we labeled them with CRISPR-Sirius-8XMS2 and the MS2 capsid protein MCP-HaloTag, which contains a nuclear localization sequence.^55^ We visualized 2-3 pairs of loci in individual cells and quantified the average distance between the nuclear center and either the *XXYLT1* or *TCF3* gene loci, normalized to the nuclear radius **(Fig. 7F-H)**. Our results demonstrated that the average distance between the nuclear center and *XXYLT1* significantly decreased upon lamin degradation, indicating that the targeted loci moved towards the nuclear interior. In conjunction with redistribution of heterochromatin and nuclear wide changes in chromatin mobility, the average distance between the nuclear center and *TCF3* also decreased, although to a less significant degree. These changes in subnuclear localization preferences indicate that B-type lamins constrain chromatin dynamics and gene positioning, especially at the nuclear periphery.

**Point 8:** For gene expression alteration upon acute B-type lamin depletion, the authors performed RNAseq and analyzed data within both LADs and non-LADs. I have several questions for this part. First, I am not sure for the untreated group whether the authors applied mock transfection, which is crucial for using the untreated group as the control and basal level for examining DEGs. Second, the data of the group "6 day withdraw DEGs" (Figure S6B) have hundreds of Up and Down DEGs that are similar with the 12 hr group. I do not see how the authors make a conclusion that "Further, restoration of B-type lamins after growing HCT116LMN(B1&B2)-AID cells in auxin-free media for 6 days after 48 hours of auxin treatment returned cells to basal levels of gene expression." Third, what is the main mechanism that causes Up and Down DEGs? Gene relocation? Are XXYLT1 and TCF3 up or down DEGs?

**Response:** We would like to clarify that for the bulk RNA-seq data collection, a mock transfection was not necessary to determine the levels of gene expression between time points. From what we understand, a mock transfection is necessary when a transfection reagent is used in the treated condition. This is because our purpose of doing bulk-RNA sequencing was specifically to determine how gene expression was altered over the course of B-type lamin degradation. Therefore, we decided to test the 0-hour, 12-hour, 48-hour, and 48-hour with a 6-day withdraw of auxin treatment time points. To avoid confusion, we have ensured that the text specifies that we tested these conditions only within the HCT116 Lamin B1/B2-AID-mClover cells. We have since removed the statements regarding the 6-day withdraw condition and basal levels of expression.

**Please refer to lines 533-537.**

Although the changes in mesoscale chromatin structure were muted, we hypothesized that alterations in chromatin dynamics and gene positioning could still result in profound changes in gene expression. To test this, we utilized RNA-seq to measure gene expression changes between untreated cells (0-hour auxin) to 12-hour auxin-treated cells, 48-hour auxin-treated cells, and cells that were auxin-treated for 48 hours followed by a 6-day wash off **(Fig. 8A)**.

Based on the DamID and bulk RNAseq data, we propose that LAD partitioning or redistribution may be a potential mechanism that contributes to Up and Down DEGs. Recent findings from The University of Virginia have found that downregulation of lamin B1 is closely associated with nuclear morphology changes in mouse and human Nonalcoholic fatty liver disease (NAFLD) (Wei et al., 2022; Genome Research, 32 (11-12):1981-1992). In this paper, we reported that LMNB1 downregulation and nuclear shape change lead to redistribution of LADs, followed by up-regulation of lipid synthesis and storage genes. Changes in nuclear morphology alter LAD partitioning. We also found that the Lamin B Receptor is highly upregulated, while LMNB1 is downregulated, in mouse livers on WD treatment at 12 weeks. Therefore, LAD partitioning/ redistribution may be a mechanism, among others, that causes Up and Down DEGs upon Lamin B1 and B2 degradation.

We would like to clarify that neither *XXYLT1* nor *TCF3* were differentially expressed. They were chosen here as targets because they are located on Chromosome 3 and 19, respectively, and have tandem repeats that makes them suitable for CRISPR-Sirius within live cells. The gene targets on Chromosome 3 were preferentially localized near the nuclear periphery, while gene targets on Chromosome 19 were preferentially localized closer to the nuclear interior, based on previous confocal imaging. To address the reviewer’s concerns, we have since applied FISH to label genomic loci that were differentially expressed in fixed cells. The genes that were upregulated (Up DEGs) used in these FISH experiments are *CEM1P* (Chr15:80,779,370-80,951,771) and *SLC03A1* (Chr15:91,853,708-92,172,435). Both genes are within LADs based on the DamID data. We also performed FISH with a gene that was downregulated (Down DEG) known as *CYP1A1* (Chr15:74,719,542-74,725,528). This gene is located outside of a LAD based on the DamID data. **Please refer to Figure 8C-E. Please refer to lines 574-594.**

To understand the potential mechanism behind differential gene expression upon B-type lamin degradation, we utilized Fluorescence *in situ* Hybridization (FISH) to label genomic loci that were differentially expressed. Specifically, we identified *SLC03A1* and *CEM1P*, both genes on chromosome 15 that were upregulated, within a few megabase pairs apart, and within LADs based on the public DamID data. We also performed FISH with *CYP1A1*, a gene on the same chromosome that was downregulated and located outside of a LAD based on the DamID data.  First, we visualized pairs of each locus and confirmed that the two genes within LADs shifted towards the nuclear interior upon auxin treatment, consistent with *XXYLT1* in the CRISPR-Sirius experiments **(Fig. 8C-D)**. We then measured their relative distances between each other before and after B-type lamin degradation. We found that the relative distance between *SLC03A1* and *CEM1P* within nearby LADs did not change, but the distance between *SLC03A1* and *CYP1A1* outside of a LAD decreased **(Fig. 8C; Fig. 8E)**.  We interpret this mobility of gene loci as LADs disassociating from the nuclear periphery and moving into the interior nuclear space as cohesive units. This cohesive internal shift of gene loci is in line with the reduction of H3K9me3 intensity at the nuclear periphery demonstrated in SMLM experiments. Based on these results, LAD redistribution may be one of the mechanisms that causes differential gene expression after lamin degradation, although genes within LADs were not entirely upregulated **(Fig. S8A)**.  Our data support a model in which B-type lamin degradation does not significantly alter genome topology, but induces significantly increased chromatin mobility (e.g., re-localization of peripherally associated chromosomes, gene loci, and heterochromatin) at the nuclear scale to facilitate differential gene expression within and around LADs **(Fig. 8D)**.

**Point 9:** Regarding the action mechanism of B-type lamins, the authors claimed that "Our findings suggest that the mechanistic role of B-type lamins is stabilization of heterochromatin and chromosomal positioning along the nuclear periphery" and "mesoscale-motion dependent mechanism". I do not think the data in the current manuscript have provided sufficient evidence to support these claims. They need to do chromosome painting to measure the size and distribution of certain chromosomes and chromatin domains. They also need to provide additional evidence to support the "mesoscale-motion dependent mechanism" by manipulating mesoscale-motion with approaches other than lamin depletion.

**Response:** To address the reviewer’s concerns, we have completed two biological replicates of chromosome painting each for Chromosomes 1, 2, 18, and 19. We first measured the fraction of nuclei covered by each chromosome. Our results indicate that there is no significant change in chromosomal area for each of the chromosomes. Regarding the distribution of these chromosomes, however, we noticed a large spread in the distribution of Chromosome 1. Conversely, we also noticed that there was a very small spread in the distribution of Chromosome 19. **Please refer to Figures 5A-B. Please refer to lines 326-335.**

To investigate the observed mechanism behind minimal changes in higher-order chromatin folding structure, we performed two biological replicates of chromosome painting for chromosomes 1, 2, 18, and 19. We hypothesized that if B-type lamin depletion induced a shift in chromatin spatial localization at the chromosome level, then scenario (2) was more likely as it indicated an active interaction between the lamins and position maintenance. Chromosomes 1 and 2 were chosen to represent chromosomes localized relatively near the nuclear periphery while chromosomes 18 and 19 were chosen to represent chromosomes with preferential localization at the nuclear interior. We first measured the fraction of nuclei covered by each chromosome **(Fig. 5A-B)**. Our results indicate that there is no significant change in chromosomal area for each of the chromosomes.

To further test that the mechanistic role of B-type lamins is in stabilization of heterochromatin and chromosomal positioning along the nuclear periphery, we measured the distribution of these chromosomes by partitioning each nucleus into 10 bins of equal radius.

To account for differences in area for each ring, we normalized these values using the equation 2k-1, where k is the ring number. For example, the values in ring number 5 were divided by 9. The following normalized data has since been added to the main figures of the manuscript:

Using this normalization, we can now clearly see that Chromosomes 1 and 2 are preferentially localized to the nuclear periphery, whereas Chromosomes 18 and 19 are localized more internally and spread more equally among the rings. Importantly, Chromosomes 1 and 2 have a clear decrease in occupancy at the nuclear periphery upon B-type lamin removal (i.e., Rings 9-10), with a resulting increase in this occupancy towards the center (i.e., Rings 3-7).

To make the interpretation of this data even clearer, we have also plotted the percent change between these rings for each chromosome and condition. This data has since been added to the Supplementary Information. **Please refer to Figures 5C and S3A.** **Please refer to lines 337-349.**

To further explore the mechanistic role of B-type lamins in stabilization of chromosomal positioning along the nuclear periphery, we measured the distribution of each chromosome by partitioning each nucleus into 10 bins of equal radius. To account for differences in area for each ring, we normalized these values using the equation 2k-1, where k is the ring number. Our results verified that chromosomes 1 and 2 are preferentially localized to the nuclear periphery, whereas chromosomes 18 and 19 are localized more internally and spread more equally among the rings **(Fig. 5B-C)**. Importantly, chromosome 1 had a clear decrease in occupancy at the nuclear periphery upon B-type lamin removal (i.e., rings 9-10), with a resulting increase in this occupancy towards the nuclear interior (i.e., rings 3-7) **(Fig. 5C; Fig. S3A)**. Chromosome 2 occupancy was slightly reduced at ring 8, while the occupancy of chromosomes 18 and 19 remained primarily at the nuclear interior despite an increase at ring 10. This increased fraction of internal chromosomes appearing in the outmost ring could suggest a more accessible chromatin state at the nuclear periphery.

To determine if there was a difference in the chromosome occupancy of nuclei between the control and auxin conditions, we then compiled all 10 rings. Our results indicate no significant difference in the means between conditions, but we noticed a larger spread in the distribution of chromosomes with preferential localization in the nuclear interior (Chromosomes 18 and 19). We also calculated the coefficient of variation (CoV) for each condition and chromosome, which is obtained by dividing the standard by the mean and is expressed as a percentage. Our results demonstrate that there is a lower sample variability in the auxin treated condition for all samples. We have updated the text and figures accordingly. **Please refer to Figure S3B. Please refer to lines 351-360.**

To determine if there was a difference in the chromosome occupancy of nuclei between the control and auxin conditions, we then compiled all 10 rings **(Fig. S3B)**. Our results indicated no significant difference in the means between conditions. We also calculated the coefficient of variation (CoV) for each condition and chromosome, which is obtained by dividing the standard deviation by the mean and is expressed as a percentage. Our results demonstrated a lower sample variability in the auxin treated condition for all samples, indicating that when B-type lamins are disrupted, chromosomes tend to have a more homogenous spread throughout the nuclear area. Overall, this physical expansion of chromosome territories paired with the observed minimal changes at the nuclear scale further suggests that B-type lamins regulate chromatin subcellular localization using a motion-dependent mechanism.

Finally, to provide further support the "mesoscale-motion dependent mechanism" by manipulating mesoscale-motion with approaches other than lamin depletion, we have treated cells with GSK343 in the WT (wild type) HCT116 cell line. GSK343 is an inhibitor of EZH2, which has been shown to prevent H3K27me3. Using Dual-PWS, our results indicate that 24 hours of GSK343 treatment to HCT116 cells induces a change in the fractional moving mass, independent of lamin B1/B2 degradation. Based on SMLM imaging, we can see a clear increase and restructuring of heterochromatin at the nuclear periphery that produces a significant change in chromatin dynamics. We believe this helps further verify the proposed mesoscale-motion dependent mechanism. **Please refer to Figure S6B-D. Please refer to lines 481-498.**

We found that upon 24 hours of auxin treatment, H3K9me3 NSI was significantly reduced at the nuclear periphery by 25% in comparison a 0.9% increase in NSI at the nuclear interior **(Fig. 7D-E)**. To compare the effect of heterochromatin degradation on spatial positioning and chromatin mobility, we treated HCT116 cells with GSK343, a potent EZH2 inhibitor that specifically prevents H3K27 methylation. In contrast to B-type lamin depletion, we found that upon 24 hours of GSK343 treatment, H3K9me3 NSI was significantly increased at the nuclear periphery by 44% in comparison a 0.7% decrease in NSI at the nuclear interior **(Fig. S6B-C)**. Consequently, live-cell Dual-PWS measurements confirmed a reduction in both *D* and chromatin mobility **(Fig. S6D)**. These results demonstrate that reduced B-type lamins and the removal of heterochromatic marks do not result in the same phenotype, indicative of separate contributions to proper genome organization despite their preferential interaction with each other. In line with scenario (2), these results point to a B-type lamin-dependent role in restricting chromatin mobility and maintaining the spatial positioning of heterochromatin, while histone methylation maintains a repressive chromatin environment in coordination with other cellular processes that could oppose the spreading of heterochromatic domains. Taken together, these results indicate that although mesoscale organization is maintained, lamin B1 and B2 reduction induces heterochromatin destabilization from the nuclear lamina, suggesting the detachment of LADs from the inner nuclear matrix as well.

**Point 10:** Lastly, I feel that the following logic makes more sense to reveal the function of lamins: investigate mesoscale chromatin structure -> Chromosome painting size of chromosome and chromatin domains, distribution of chromosome and heterochromatin -> chromatin and gene dynamics -> gene expression

**Response:** We would like to thank the reviewer for their constructive feedback and suggestions. We have since modified the manuscript text and figures to match this suggested structure.

Reviewer #3:

**Remarks to the Author:** In this manuscript, Pujadas and colleagues utilise a chemically inducible degron system in human cells to deplete LAMINB1 and B2. They utilise this acute loss of function system to investigate the impact of LAMINB loss on cell fitness, nuclear organisation, chromatin architecture and gene regulation. The paper is driven by the hypothesis that LAMINB1 and B2, as critical structural components of the nuclear periphery, serve to create a repressive compartment for peripheral associated genes. The authors conclude that loss of LAMINB1 and B2 has little impact of mid-range chromatin folding (compartmentalisation, TADs etc) but leads to pronounced changes in gene expression concomitant with increased chromatin mobility within the nucleus. Interestingly the changes in gene expression seem not to follow the expectations that genes associated with the nuclear periphery are preferentially repressed.

The subject of this study addresses an important intersection between nuclear organisation, chromatin architecture and gene regulation. The system employed for depletion, the quality of the Hi-C data and the gene expression data are of significant interest. However this reviewer does not feel that some of the other assays performed are robust or well enough explained to support some of the conclusions, and thus to allow for publication in as currently presented. Therefore, I believe that the authors must address the following concerns and revise the manuscript accordingly before the manuscript can be accepted for publication in Genome Biology.

**Response:** We would like to thank the reviewer for their careful consideration of our manuscript and have addressed their specific comments below.

**Major Comments**

**Clarity:**

**Point 1:** There are various issues with the clarity of the text and figures, these need to be improved (for specific examples see minor comments below). In addition, some sections should be removed as they are not robust and distract from the main findings of the study (see below).

**Response:** We would like to thank the reviewer for their specific suggestions and have addressed each of their comments in the text and figures. We have also removed the suggested figures/ statements to enhance clarity.

**Point 2:** Fig S4E – The clover signal shown on the X axis should not be plotted as an inverse as it makes the interpretation of the relationship between D and LAMINB much less clear. Indeed page 13 – Line 316 should read strong anti-correlation not correlation.

**Response:** To address the reviewer’s concerns, we have replotted this figure to not be an inverse relationship. Further, we have corrected the wording in the text for consistency. **Please refer to Figure S4C. Please refer to lines 393-396.**

To confirm this result, we verified a strong anti-correlation between the change in *D* and mClover signal with a 24-hour auxin treatment time course and confirmed that OsTIR1 expression induced by doxycycline treatment does not alter *D* **(Fig. S4C-D)**.

**Point 3:** In several instances, the figure panels are discussed in the wrong order in the text and this should be corrected. For details see below in minor corrections.

**Response:** We have corrected the text and figure panels to be sequential and have addressed the other minor corrections suggested by the reviewer.

**Control experiments:**

**Point 4:** The functional normality of tagged lines is assessed based on the nuclear staining shown in Figure 1, however there are other important test that are required also. Do the targeted cells grow as parental e.g. cell cycling, gene expression, genome organisation in the absence of TIR1 indication? Some additional validation is required to address at least some of these concerns, or justification as to how they have been mitigated.

**Response:** We agree that there is a need to further demonstrate the functional normality of the tagged cell lines. To address the reviewer’s concerns, we have conducted additional experiments and have included them in the modified figures We have chosen specific data to include in the updated manuscript.

Specifically, we have completed live-cell imaging in 96-well plates in triplicate to compare the growth rates of the parental cell line (HCT116) and tagged cell line (HCT116 Lamin B1/B2-AID-mClover) with and without the addition of doxycycline to induce TIR1, as well as with and without the addition of both doxycycline and auxin to degrade B-type lamins. We collected growth rate data every 2 hours for a total of 72 hours (normalized to the 0-hour condition). These studies revealed that the tagged cell line appears to grow at the same rate as the parental cell line up to about 35 hours. Most data within this manuscript were collected at the auxin-treated 24-hour time point. The tagged cell line has a slower growth rate when treated with either doxycycline alone (black triangles) or with both doxycycline and auxin treatment (green triangles).  When the parental cell line was treated with both doxycycline and auxin (green circles), the growth rate was also slower than that of the untreated parental cell line (black circles), indicating that the auxin treatment itself may have a negative effect on cell growth. **Please refer to Figure S1A. Please refer to lines 183-191.**

To further test cellular functionality, we compared the growth rates of the parental cell line (WT HCT116; WT refers to wild type) and one of the tagged cell lines (HCT116^LMN(B1&B2)-AID^) with and without the addition of doxycycline to induce OsTIR1, as well as with and without the addition of both doxycycline and auxin to degrade B-type lamins **(Fig. S1A)**. HCT116^LMN(B1&B2)-AID^ treated with both doxycycline and auxin had a nearly identical growth rate as the parental cell line but slowed after about 35 hours of treatment. Doxycycline treatment alone resulted in a slightly lower growth rate than that of the parental cell line after about 30 hours of treatment as well. Therefore, although the addition of the mAID tag does not alter proper localization of lamins, our findings suggest that B-type lamins play a role in cell proliferation.

Further, we conducted three biological replicates of flow-cytometry based cell cycle analysis using propidium iodide staining. Our results indicate that the untreated tagged cells are slightly stalled in the G0 – G1 phase in comparison to the untreated parental cells. The tagged cells become arrested at the G0 – G1 phase even more when B-type lamins are degraded at 24 and 72 hours of auxin treatment. Subsequently, there were less tagged cells within the S phase and G2-M phase at 48 hours of auxin treatment.

Based on these results, we can temper our conclusions to say that although the tagged cells have a slightly slower growth rate than the parental cells, the addition of doxycycline and auxin to remove B-type lamins results in the most substantial differences in cell functionality (i.e., growth rate, cell-cycling). We have since updated the text and figures to include these additional analyses. We have included the most relevant data in the updated text and figures. **Please refer to Figure 2A. Please refer to lines 214-220.**

To further investigate the role of B-type lamins in retaining cell function and assess the impacts of a slowed growth rate, we applied flow-cytometry based cell cycle analysis using propidium iodide staining **(Fig. 2A)**. Our results indicate that the untreated HCT116^LMN(B1&B2)-AID^ cells were stalled in the G0 – G1 phase in comparison to the untreated parental cells. The HCT116^LMN(B1&B2)-AID^ cells became arrested at the G0 – G1 phase even more so when B-type lamins were degraded at 24 and 72 hours of auxin treatment. Subsequently, there were less HCT116^LMN(B1&B2)-AID^ cells within the S phase and G2-M phase at 48 hours of auxin treatment.

**Point 5:** Incomplete loss of Lamin B1 and 2: It is commendable that the authors utilise the clover as a sensitive indicator of degradation, however it does highlight that depletion is not complete compared to controls within the experimental times used (see Figure 1F / S1B / S1D). There is also no explanation of what the two tables are in S1. The labelling appears to suggest they are the same information, so are these quantifications for individual replicates or the values from different tagged lines? Furthermore on Page 8 (line 197/198) it is stated that nearly 80% of LMNB1-mAID was degraded within the first 6 hours of auxin treatment. What data is this based upon? This does not look to be the case in the FACs analysis and if this is based on the immunoblot this should be explicitly stated. Whilst it is likely that many published degron studies have incomplete loss, you have clearly demonstrated it here by comparing the blot to the clover FACS. Consequently you must, at the very least, comment on this and temper your conclusions to include the caveat that there is likely residual LAMINB1 and B2 left and this may limit the extent of the phenotypes assayed here.

**Response:** We would like to clarify that the statement regarding 80% of LMNB1-mAID degradation is based on Western Blot (WB) results (Fig. 1E) and confirm that there is in fact this caveat of residual B-type lamin proteins upon auxin treatment. Mechanistically, the dosage of doxycycline to induce TIR1, IAA ligand (auxin), and the AID-tagged target protein(s) are all key determinants of degradation efficiency (Macdonald et al., 2022; eLife). The flow cytometry results emphasize that there is a difference in LMNB1/B2 degradation between 500 µm and 1000µm IAA treatment (Fig. 1D).

We would like to highlight that the degradation effect and time of B-type lamins is different between WB and flow cytometry, due to the nature of these techniques. When endogenous LMNB1/B2 is being degraded, the WB detects LMNB1/B3 fragments that the antibody recognizes. However, flow cytometry directly detects the mClover signal which may be or may not be related to LMNB1/B2 protein. Therefore, compared to a WB, it takes a longer amount of time to demonstrate LMNB1/B2 degradation than with flow cytometry. In other words, flow cytometry is more sensitive to background than WB.

To confirm that the AID system works to degrade B-type lamins, we collected fluorescent (mClover signal) data every 2 hours for a total of 72 hours (normalized to the 0-hour condition). Our results demonstrate that at 24 hours of auxin treatment, about 70% of the mClover signal is removed. We therefore agree that like the majority of published degron studies, there is residual B-type lamins leftover, and this may limit the extent of the phenotypes assayed here in the manuscript. We have made this correction in the discussion text to temper our conclusions. **Please refer to lines 628-630.**

It should be noted, however, that the residual B-type lamins after auxin treatment and changes in cell cycling may limit the extent of the phenotypes assayed in this study.

**Incomplete or unconvincing data:**

**Point 6:** Page 9; 211-214. ‘In addition to the change in nuclear volume, confocal sections in HCT116LMN(B1&B2)-AID cells revealed lamin-specific spherical and discrete puncta, which has been previously demonstrated in the nucleus of cultured cells 36,37 (S2C).’ It is not clear why this is important or of interest. Nor is it indicated in the legend for S2C what conditions this represents - is this auxin treated or not (e.g. if yes are these the non-degraded cells analysed in S2A) or is this Auxin treated without TIR1 induction? Do you see these puncta in WT or parental cells? Unless this is an important point it is a distraction from the main points and should be removed. If it is important this should be justified and discussed.

**Response:** We agree with this reviewer’s comments and would like to clarify that this figure was referring to non-degraded cells. We agree with the reviewer that this is not an important part of our manuscript and have therefore removed it from both the text and figure panel.

**Point 7:** The concept of changes in chromatin marks is an interesting one, however the data shown is not convincing. Fig 5A and B - How is the WB quantified and are there replicates? IFs are poor quality and the analysis uninformative.

**Response:** To address the reviewer’s concerns regarding convincing and uninformative data, we have improved the representative images and have since moved this to the SI. This figure has now been replaced with updated regional analysis using CellProfiler to measure the intensity distribution of H3K9me3 and H3K27me3 before and after the addition of auxin. The radial positioning of these marks was determined as a function of the signal intensity measured in each of the ten concentric rings per nucleus. This analysis, in combination with STORM experiments, revealed that both constitutive (H3K9me3) and facultative (H3K27me3) heterochromatin was slightly reduced at the nuclear periphery in rings 8, 9, and 10. The slight increase in these chromatin modifiers closer to the nuclear center (i.e., ring 1) is indicative of a shift in these marks from the nuclear periphery towards more interior nuclear regions. We believe that this updated analysis further confirms our initial observations. **Please refer to Figure 7A and S5D.**

To confirm the global reduction in facultative heterochromatin upon B-type lamin degradation, we have repeated the WB analysis in triplicate (see below). This has since been replaced with one of the new replicates with higher image quality. **Please refer to Figure 7B.**

The text has been updated to reflect these modifications. **Please refer to lines 449-462.**

We reasoned that if LMNB1 and LMNB2 depletion leads to the detachment of chromatin domains from the nuclear periphery, as indicated by our dual-mode live-cell regional PWS results, this internal shift would be accompanied by heterochromatin redistribution. Using immunofluorescence, we found a global reduction in H3K27me3 and increase in H3K27ac upon 24 hours of auxin treatment in HCT116^LMN(B1&B2)-AID^ cells, with comparable results observed in HCT116^LMNB1-AID^ and HCT116^LMNB2-AID^ **(Fig. S5A-C)**.  Next, we confirmed an internal shift of H3K27me3 from the nuclear periphery using the same segmentation and area normalization for chromosome paint **(Fig. 7A; Fig. S5D)**. We also noticed that while H3K27ac increased slightly at the nuclear periphery in the outermost ring, in line with chromosomes 18 and 19 from chromosome painting experiments, there was an internal shift as well. To confirm that global histone methylation was reduced, we used western blot analysis to measure the amount of H3K27me3 in triplicate **(Fig. 7B; Fig. S5E)**. This analysis revealed a ~ 41% decrease in H3K27me3 upon 24 hours of auxin treatment to HCT116^LMN(B1&B2)-AID^ cells.

We have added information regarding the relative quantification of WB bands in the Methods section of the manuscript. **Please refer to lines 884-887.**

To quantify the western blot bands, we used the iBright Analysis Software from Thermo Fisher Scientific to define bands as regions of interest. By measuring the mean grey intensity values, the final relative quantification values were calculated as the ratio of each protein band relative to the lane’s loading control for all three replicates.

**Point 8:** Fig 5E – the data for TCF3 is noisy with high background and does not seem to reflect the quantification in 5F. Fig 5F – indicates distance from centre in the plot whereas the text on page 17 Line 407/408 ‘between the nuclear periphery and either the XXYLT1 or TCF3 gene loci’ indicates the distance from the periphery. The later would not fit with the plot. This needs to be corrected in the text.

**Response:** To address the reviewer’s concerns, we have replaced the *TCF3* representative images to one with a higher signal to noise ratio. Further, we have corrected the wording in the text for consistency. **Please refer to Figure 7F and 7H. Please refer to lines 516-524.**

Specifically, we chose one intronic gene region on Chromosome 3 (*XXYLT1*) containing ~ 333 endogenous tandem repeats, and one intronic gene region on Chromosome 19 (*TCF3*) containing ~ 36 endogenous tandem repeats as a reference to the nuclear interior. To measure the spatial distance and dynamics of the *XXYLT1* gene loci, we labeled them with CRISPR-Sirius-8XMS2 and the MS2 capsid protein MCP-HaloTag, which contains a nuclear localization sequence.^55^ We visualized 2-3 pairs of loci in individual cells and quantified the average distance between the nuclear center and either the *XXYLT1* or *TCF3* gene loci, normalized to the nuclear radius **(Fig. 7F-H)**.

**Point 9:** Page 18 – paragraph from 438-449. The gene expression analysis is interesting but this section lacks two pieces of key information to assess the prevalence of DEGs in LADs vs non-LADs. Firstly what fraction of the genome are scored as LADs e.g. are there less LAD genes because the fraction of the genome covered by LADs is smaller? On a similar note, LAD gene density is disproportionately low. If you factor this in – is the fraction of LAD genes that are differentially expressed more than the fraction of non-LAD genes that are differentially expressed? Also it would be better to focus the analysis on 12 h as this will be the immediate early effects and more likely to give you a clear direct influence of periphery loss (less secondary expression changes at earlier time points).

**Response:** We would like to clarify that **Figure 4B** demonstrates that Lamin B1% coverage ranges from ~ 20 – 75%. As our analyses showed a very high correlation between Lamin B1-defined LADs from DamID and Lamin B1/B2 tracks from RNA-sequencing, we interpreted Lamin B1% coverage as the fraction of the genome scored as LADs. It has been reported that human cells have approximately 1,000 – 1,500 LADs that typically range from 10 kb – 10 Mb in size (~0.5 Mb median) (Steensel et al., 2017).

We have since updated our analyses to determine what number of DEGs are within LAD or non-LAD regions at the 48-hour time point. While we agree with this reviewer that the 12-hour time point would be an interesting choice for analysis to determine the immediate early effects of B-type lamin degradation, we specifically chose the 48-hour time point because most of the B-type lamins are no longer present based on our previous flow cytometry and western blot experiments. This was also confirmed when assessing the mClover signal over a 72-hour time course experiment (omitted from the revised manuscript but included this plot in the response to Point 5 above).

**Please refer to Figures 8B and S7D.** **Please refer to lines 548-563.**

Given the global changes in nuclear organization observed, we hypothesized that depletion of B-type lamins would result in differential gene expression within both LAD and non-LAD territories. We anticipated that genes within LADs would experience upregulation once freed from the heterochromatic nuclear periphery. Likewise, as mesoscale structure was preserved, we anticipated that there would be limited association between gene expression changes and TAD structure**.** Using the same publicly available Lamin B1 DamID data from HCT116 cells, we again segmented the genome into regions within LADs and regions outside LADs^44^. After 48 hours of lamin depletion, we identified nearly 22% of enriched genes within LADs (adjusted P value < 0.01 and absolute log fold change > 1) **(Fig 8B; Fig. S7A-B)**. Anomalously, lamin B1 and lamin B2 depletion predominantly generated the highest number DEGs outside of LAD boundaries for all three timepoints, with the greatest number at 48 hours of auxin treatment (374 genes within LADs, 1331 genes outside of LADs at 48 hours) **(Fig. S7A-B)**. Further, the log fold change of DEGs outside of LADs was larger in magnitude than within LADs, indicating that they were disproportionally influenced by loss of B-type lamins **(Fig. S7C)**. Unexpectedly, in LAD regions, there was no clear trend towards upregulation or downregulation in gene expression. In non-LAD regions however, there appeared to be more upregulated genes **(Fig. 8B; Fig. S7D)**.

**Methods:**

**Point 10:** For CRISPR-syrius (P16 lines 401-404). ‘Specifically, we chose one intronic gene region on Chromosome 3 (XXYLT1) containing ~ 333 tandem repeats and one intronic gene region on Chromosome 19 (TCF3) containing ~ 36 tandem repeats as a reference to the nuclear interior’. It is unclear where the repeats for the two studied genes come from. Are these endogenous repeats within the genes targeted by CRISPR or are these engineered into the cells? Whilst this is a very important question in the paper, the data (particularly the examples) are poor and do not represent the observed trend that is being discussed. Whilst this is a nice approach, it is unclear why a conventional approach e.g. DNA FISH was not employed here.

**Response:** We would like to clarify that the repeats or the two studied genes are endogenous repeats and have not been engineered into the cells. To find these targets, we utilized CRISPRbar (Ma et al., 2018; Nature Methods), a tool developed by Thoru Pederson’s group. This tool allows the user to look at all repetitive regions on a particular chromosome and is a publicly available resource. This has since been modified in the text as stated in the last reviewer response. **Please refer to lines 508-516.**

We understand that the representative images are not obvious for CRISPR-Sirius and have replaced the TCF3 image with a sharper image. Therefore, we have completed FISH experiments to measure the relative movement of upregulated genes within a LAD (*SLC03A1*) versus outside of a LAD (*CYP1A1*). Our results further confirm that genes near the nuclear periphery (i.e., *SLC03A1 and CEM1P*) move more internally upon the addition of auxin, whereas genes more preferentially localized to interior regions (i.e., *CYP1A1*) appear to move slightly inward or stay relatively within the same location.

Our rationale for including both CRISPR-Sirius and FISH in the revised manuscript is to demonstrate that in both live and fixed cells, respectively, the results essentially demonstrate the same trends. **Please refer to Figure 7F and Figure 8C-E. Please refer to lines 574-590.**

To understand the potential mechanism behind differential gene expression upon B-type lamin degradation, we utilized Fluorescence *in situ* Hybridization (FISH) to label genomic loci that were differentially expressed. Specifically, we identified *SLC03A1* and *CEM1P*, both genes on chromosome 15 that were upregulated, within a few megabase pairs apart, and within LADs based on the public DamID data. We also performed FISH with *CYP1A1*, a gene on the same chromosome that was downregulated and located outside of a LAD based on the DamID data.  First, we visualized pairs of each locus and confirmed that the two genes within LADs shifted towards the nuclear interior upon auxin treatment, consistent with *XXYLT1* in the CRISPR-Sirius experiments **(Fig. 8C-D)**. We then measured their relative distances between each other before and after B-type lamin degradation. We found that the relative distance between *SLC03A1* and *CEM1P* within nearby LADs did not change, but the distance between *SLC03A1* and *CYP1A1* outside of a LAD decreased **(Fig. 8C; Fig. 8E)**.  We interpret this mobility of gene loci as LADs disassociating from the nuclear periphery and moving into the interior nuclear space as cohesive units. This cohesive internal shift of gene loci is in line with the reduction of H3K9me3 intensity at the nuclear periphery demonstrated in SMLM experiments. Based on these results, LAD redistribution may be one of the mechanisms that causes differential gene expression after lamin degradation, although genes within LADs were not entirely upregulated **(Fig. S8A)**.

**Point 11:** Page 15-16 lines 379-382. This conclusion is interesting but is not supported by robust data. This needs to be clarified or supported by additional data.

**Response:** To address this reviewer’s comment, we have since completed additional experiments that provide further evidence of chromatin destabilization at the nuclear periphery and a shift in heterochromatin upon B-type lamin disruption. Specifically, we have provided chromosome painting experiments that show chromosomes at the periphery moving internally, a regional internal shift and global reduction of H3K27me3 based on confocal and western blot assays, along with single molecule localization microscopy (SMLM) to measure the normalized H3K9me3 intensity at the nuclear periphery compared to the nuclear interior. Further, the dynamic PWS results illustrate a relatively large increase in fractional moving mass, meaning that there is greater movement of chromatin throughout the nuclear region in live cells. Measuring the coefficient of variation also highlights that there is overall chromatin decompaction, which is in line with greater chromatin mobility and “open chromatin” that spatially allows for increased acetylation. Our FISH and modified RNA-sequencing results also support this conclusion by demonstrating gene-specific changes within and outside of LADs. **Please refer to Main Figures 5B-C, 6F-H, 7A, 7D-E, 8C-E and Supplementary Figures S3A, S4G-I, and S5D, S6B-D.**

**Minor Comments / corrections:**

**Point 12:** Page 7; line 168 Fig 1D referred to before 1C – re-order figure or text accordingly.

**Response:** We have made this correction in the text accordingly. Throughout the manuscript, we have now ensured that all figures are referred to in sequential order, including supplementary figures and tables.

**Point 13:** Page 7; line 177-178 – ‘Genomic localization has previously demonstrated utility as an indicator of functional retention in transcription factor binding’. It is unclear what this means – please rephrase.

**Response:** With this statement, we were referring to two specific studies in which protein subcellular localization is indicative of normal cellular function. First, research from the Max Planck Institute of Biotechnology applied global mapping of protein translocation events to demonstrate that protein function is determined by subcellular localization because of preferential chemical environments and interaction partners (Itzhak et al., 2016). Second, researchers from the University of Texas described how aberrant localization of proteins within the nucleus contributes to the pathogenesis of several human diseases (Hung and Link et al., 2011).

However, we understand that this statement in the manuscript is unclear and have modified the text accordingly. **Please refer to lines 172-183.**

To confirm that addition of the mAID tag does not alter the functionality of the tagged lamin proteins, we first used immunofluorescence to ensure the continued association of lamin B1/B2 with other proteins that localize to the same compartment as the untagged proteins (i.e., lamin A/C). Several studies have demonstrated that subcellular localization is essential for proper protein function and biological processes, and that mis-location is strongly associated with human disease. Protein function is determined by subcellular localization due to preferential chemical environments and interaction partners. As expected, our results indicate that LMNB1-mAID-mClover, LMNB2-mAID-mClover, and LMNB1/B2-mAID-mClover proteins localize properly to the nuclear lamina in cells **(Fig. 1C)**. We observed a strong overlap between the mClover-signal (hence LMNB1-AID and/ or LMNB2-AID) and Lamin A/C immunostaining signal. This result suggests that addition of the mAID-mClover tag does not alter proper localization or protein function of LMNB1 and LMNB2.

**Point 14:** Figure 1C. The full genotype should be shown above the panel e.g. LMN(B1&B2)-AID as the shorthand is confusing as it resembles the naming of the staining/label. The same is also noted in figure S1A, and although the figure is transposed, cell line vs the staining/tag should be made clearer.

**Response:** We have made this correction in the figure panels. **Please refer to Figure 1C.**

**Point 15:** Figure 1G, S2A-B – the effect size is small, how does this compare to WT or parental cell controls?

**Response:** For these figures, we measured the DAPI-stained nuclear area of the LMNB1-mAID-mClover, LMNB2-mAID-mClover, and LMNB1/B2-mAID-mClover cell lines to directly compare the untreated versus auxin-treated cells.

While we did not include the parental cell line (WT HCT116) in this flow cytometry assay at the time, we measured the DAPI-stained area of these parental cells using confocal microscopy. The DAPI-stained area of the parental cells was slightly larger than that of the untreated tagged cell lines. However, the effect size remains approximately the same for the untreated tagged cell lines when compared to the auxin-treated LMNB1-mAID-mClover, LMNB2-mAID-mClover, and LMNB1/B2-mAID-mClover cells. **Please refer to Figure S1B.**

**Point 16:** Fig 2A. It would be useful to see some additional zoomed in shots of the Hi-C plots as the whole genome views are not informative at this scale. It would be interesting in particular, to see regions associated with LADs and non-LADs as a reference for later sections.

**Response:** We appreciate this suggestion from the reviewer and have included an additional zoomed in shots of the Hi-C matrix for chromosome 5 in LMNB1/B2-mAID-mClover cells. **Please refer to Figure 3G.** As shown, we computationally segmented the genome into contacts that spanned both LADs and non-LADs (across each), across only LADs, or across only non-LADs. This was done for both the control and 24-hour auxin conditions. As demonstrated, we could not detect any changes in these regions after 24-hour auxin treatment.

We have also replaced the Hi-C maps in the main and supplemental figures to demonstrate these minimal changes at 15 kb resolution for both chromosome 1 and 15. **Please refer to Figure 3A and Figures S2A-B.**

We have not included the following zoomed in shots of Hi-C plots with the merged Lamin B1-DamID track in the revised manuscript but have demonstrated them below to highlight the lack of change seen in Hi-C maps (visualized on Juicer Web). Here, we have selected chromosome 1 because it localized to the nuclear periphery and repositioned towards the nuclear center upon lamin removal, based on our chromosome paint experiments that have been included in the revised manuscript:

**Point 17:** Fig 2B – need to label A and B compartments on the plots.

**Response:** We have made this correction in the figure panel. **Please refer to Figure 3B.**

**Point 18:** Figure 2D. There is a subtle reduction in size and increase in number of TADs. This presumably means that some TADs called as one large TAD in control cell Hi-C become fragmented and identified as separate sub-TADs following LAMINB loss. Is this the case and is there any distinction between where these TADs are relative to LADs?

**Response:** We appreciate the concern of this reviewer and agree that there is a potential that some TADs are identified as separate sub-TADs following loss of B-type lamins. We used TopDom, developed by Shin et al., 2015 which detects TADs with a fixed window, but is not always flexible enough to detect TADs of different sizes and hierarchies in comparison to other TAD callers such as OnTAD. First, TopDom calculates the binSignal (average contact frequencies between upstream and downstream regions within a fixed window). It then detects the local minima of the binSignal curve, and then filters the false-positive minima. This is done by testing the difference between and within interactions using the Wicoxon rank-sum test. From here, regions between two consecutive local minima are annotated as TADs.

Despite the possibility of missing TADs of different sizes due to TopDom having a window size as it’s single key parameter, we can still assess the relative sizes and number of TADs per chromosome. **Please refer to Figures S2D.** Across nearly every chromosome after B-type lamin degradation, there is a slight increase in TADs (more self-interaction).

Importantly, we can also pair the sequencing-based methods such as Hi-C, RNA-sequencing, and DamID to obtain information about which differentially expressed genes are found within/ outside of LADs as well as within/ outside of TADs. **Please refer to Figures S7E.** While visualizing TADs in relation to LADs using the chromatin imaging methods utilized in this paper is outside of the scope of this research, integrating these sequencing-based methods can at least confirm that LADs and TADs often overlap as other groups have previously reported (Merkenschlager and Nora et al., 2016 for example). However, we find from these results that there are also many instances in which they do not overlap, which has been reported by several groups as well (Dixon et al., 2012; Nora et al., 2012).

**Point 19:** Page 10 lines 247/248. Fic 2C is referenced after 2D in the text.

**Response:** We have made this correction in the text accordingly. Throughout the manuscript, we have now ensured that all figures are referred to in sequential order, including supplementary figures and tables. **For example, please refer to lines 259-275.**

**Point 20:** Page 11 lines 260-265. Fig 3E is referred to before 3B-D.

**Response:** We have made this correction in the text accordingly. Throughout the manuscript, we have now ensured that all figures are referred to in sequential order, including supplementary figures and tables. **For example, please refer to lines 281-299.**

**Point 21:** Fig 3E. Has statistical analysis been performed on this? What is the result?

**Response:** We would like to clarify that we did a paired t-test to calculate significance. It has since been added to both the figure panel and figure legend. **Please refer to Figure 3D.**

**Point 22:** Fig4 – The y axis label of D looks like panel labels – needs to tidy up the figure for clarity.

**Response:** We have made this correction in the figure panel. **Please refer to Figures 6B and 6F.**

**Point 23:** Page 13 – lines 324-330. This section needs to be clarified to make it easier to follow to a non-specialist what the underlying biology you are looking at is here.

**Response:** We have since modified the text to give more biological meaning to this statement. **Please refer to lines 417-423.**

Taken together, our temporal analysis indicates that B-type lamin degradation results in overall greater magnitude of chromatin chain fluctuations with a slower rate of motion. Such a phenotype would be observed with the dissociation of large structures from a confined state in a crowded environment. Although Dual-PWS is not a molecularly specific technique, this result could be explained by the weaking of chromatin and nuclear interactions at the nuclear periphery. These findings therefore provide further evidence that increased chromatin mobility due to a loss of B-type lamins at the periphery induces conformational changes, even in the nuclear interior.

**Point 24:** Page 14 – lines 340-342. ‘Taken together, our temporal analysis indicates that B-type lamin degradation results in overall greater magnitude of chromatin turnover with a slower rate of motion’. What does this actually mean for chromatin in the nucleus; turnover of what?

**Response:** We would like to clarify that by chromatin turnover, we were referring to the increase in average fluctuations of the chromatin chain, represented by the fractional moving mass measurement. The slower rate of motion refers to the diffusion coefficient measurement. We have made the following changes to the text. **Please refer to lines 446-452.**

Overall, these findings further suggest a mechanistic role of lamin B1 and lamin B2 in the confinement of chromatin mobility throughout the cell nucleus. Our interpretation of these results is that when lamin B1 and lamin B2 are degraded, chromatin domains slowly move away from the nuclear periphery with a resulting shift towards the nuclear interior. We reasoned that if LMNB1 and LMNB2 depletion leads to the detachment of chromatin domains from the nuclear periphery, as indicated by our dual-mode live-cell regional PWS results, this internal shift would be accompanied by heterochromatin redistribution.

**Point 25:** Fig 5D – I think the y label should read foci not loci.

**Response:** We have made this correction in the figure panel. Please refer to **Figure 7G**.

**2^nd^ round**

**Reviewer 1**

The authors have done a phenomenal job in revising their manuscript, with supplemental experiments, analyses and more focused and pertinent interpretations and conclusions. After some minor editorial / syntax / typo corrections, the manuscript should be in my opinion acceptable for publication.

**Reviewer 2**

The authors have conducted extensive revision which have my concerns well adhessed.

**Reviewer 3**

I have reviewed the authors responses to my comments, and to a lesser degree the responses to the other reviewers, and feel that they have sufficiently addressed our concerns. There have been substantial alterations, clarifications and additions that strengthen the manuscript. This is highlighted in the abstract which is substantially clearer than the initial submission. I recommend the article for publication subject to the qualification I have noted to the editorial team.
